# Synthesis of Benzocycloalkanone-Based Michael Acceptors and Biological Activities as Antimalarial and Antitrypanosomal Agents

**DOI:** 10.3390/molecules28145569

**Published:** 2023-07-21

**Authors:** Ali Mijoba, Esteban Fernandez-Moreira, Nereida Parra-Giménez, Sandra Espinosa-Tapia, Zuleyma Blanco, Hegira Ramírez, Jaime E. Charris

**Affiliations:** 1 Organic Synthesis Laboratory, Faculty of Pharmacy, Central University of Venezuela, Los Chaguaramos 1041-A, Caracas 47206, Venezuela; alimijoba@gmail.com (A.M.); blancomzule@gmail.com (Z.B.); 2 Laboratory of Parasites Physiology, Biophysics and Biochemistry Center, Instituto Venezolano de Invest Gaciones Científicas, Altos de Pipe 1020-A, Caracas 21827, Venezuela; bionereida@gmail.com; 3 Escuela de Medicina, Universidad Espíritu Santo, Samborondón 092301, Ecuador; estebanfernandez@uees.edu.ec; 4 Departamento de Química, Universidad Técnica Particular de Loja, Loja 1101608, Ecuador; sandraespinosa100@hotmail.com; 5 Facultad de Ciencias de la Salud y Desarrollo Humano, Univesidad Ecotec, Km. 13.5 Samborondón, Samborondón 092302, Ecuador

**Keywords:** malaria, *Plasmodium berghei*, *Trypanosoma cruzi*, benzocycloalkanone, Michael Acceptors

## Abstract

A series of benzocycloalkanone derivatives have been prepared and evaluated as antimalarial and antitrypanosomal agents. The compounds were obtained by direct coupling of preformed 4-substituted benzaldehyde and indanone or tetralone substitutes through aldol condensation of Claisen-Schmidt using sodium hydroxide as a catalyst in ethanol at room temperature. Although designed to inhibit the formation of β-hematin in vitro, only three compounds, **10**, **11**, and **12**, showed activities greater than 50% (75.16%, 63.02%, and 56.17%, respectively). The results of the in vivo antimalarial evaluation show that **10**, **11**, and **12** reduced parasitemia marginally, and an insignificant increase in the days of survival of the mice was observed. As trypanocidals, all compounds showed marginal activity as inhibitors of the proliferation of *T. cruzi* epimastigotes, except compound **33**, with an activity of 51.08 ± 3.4% compared to the activity shown by the reference compound benznidazole 59.99 ± 2.9%. The compounds appear to have little cytotoxic effect against VERO cells in vitro; this new class of Michael acceptor agents clearly warrants further investigation.

## 1. Introduction

A parasite is an organism that lives on or in a host organism and obtains its food from or at the expense of its host. There are three main classes of parasites that can cause disease in humans and animals: protozoa, helminths, and ectoparasites. Protozoa, together with helminths, represent the main cause of parasitic disease in humans and animals [1]. Despite great advances in modern medicine, parasitic infections continue to affect a large number of humans as well as the economies of the affected countries, either directly or indirectly. The prevalence of major human protozoan parasitic diseases (PPDs) is estimated at approximately 790 million individual cases, with an annual death toll of 810,000. Most PPDs are widely regarded as poverty-related and neglected tropical diseases that have been largely ignored for many years [2].

Chagas disease (CD), also known as American trypanosomiasis, caused by the protozoan *Trypanosoma cruzi*, is a neglected tropical disease (NTD) with high prevalence and significant morbidity and mortality. It is endemic in 21 countries; the World Health Organization (WHO) estimates the prevalence of CD in about 7 million cases, resulting in more than 8000 deaths per year. It is mostly transmitted when humans come into contact with feces and/or urine of infected blood-sucking triatomine bugs (vector-borne transmission). Other routes of transmission have been identified and include blood transfusion and congenital infections. There are even reported cases of oral infection through ingestion of contaminated foods [3,4]. Due to immigration from South and Central America, hundreds of thousands of people in countries such as Canada and the United States of America, as well as in many European and some African, Eastern Mediterranean, and Western Pacific countries, can also carry the disease [5].

**Figure 1:**
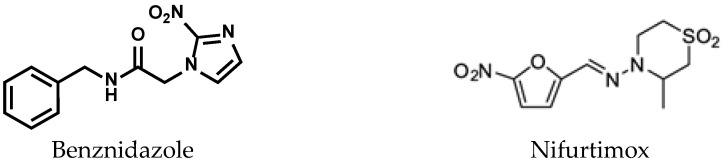
Structures of benznidazole and nifurtimox.

CD has two clinical phases: acute and chronic. The acute phase is relatively rare, with no specific symptoms detected, but it can be fatal in children. During the chronic phase that succeeds the acute phase, up to 30% of patients suffer from cardiac disorders and up to 10% experience digestive, neurological, or mixed disorders. To date, there is no perspective on an efficacious vaccine against trypanosomiasis, and the alternative is the development of safe and efficient chemotherapies to treat this disease. CD can be treated with two antiparasitic medicines: benznidazole and nifurtimox (Figure 1). Both medicines are nearly 100% effective in curing the disease if given soon after infection, including cases of congenital transmission. However, the efficacy of both diminishes the longer a person has been infected and the adverse reactions are more frequent at older age [4].

Malaria, although not included in the WHO list of NTDs, is a life-threatening disease caused by parasites that are transmitted to people through the bites of infected female *Anopheles* mosquitoes. Six parasite species cause malaria in humans, and two of these species, *P. falciparum* and *P. vivax*, pose the greatest threat. According to the world malaria report, there were an estimated 247 million malaria cases in 2021, and 619,000 estimated deaths [6].

**Figure 2:**
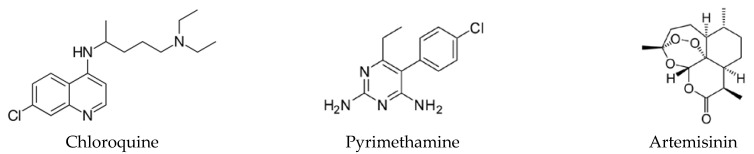
Structures of chloroquine, pyrimethamine, and artemisinin.

**Figure 3:**
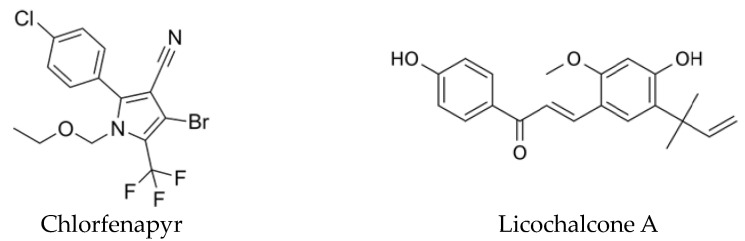
Structures of chlorfenapyr and licochalcone A.

Among the factors that have precipitated the resurgence of malaria, climate change and environmental problems are mentioned, which have degenerated into changes in the life cycle, the duration of activity, and the proliferation of *Anopheles* [7]. The other two factors to take into consideration in the resurgence of malaria are an increase in the resistance of *Anopheles* strains to residual insecticides and the resistance to standard drugs, such as chloroquine (CQ), pyrimethamine, and artemisinin, and its derivatives (Figure 2) [8,9]. However, new scientific advancements are providing new tools for malaria control, including the first effective malaria vaccine, RTS,S/AS01 (RTS,S), which was approved by the WHO in October 2022 [10], and a new chlorfenapyr long-lasting insecticidal net that could mitigate the effects of insecticide resistance among mosquitoes has been tested in a clinical trial (Figure 3) [11].

Despite the difference in epidemiology and visibility, these diseases share a similar history of strategies for their treatment and control. Various aspects of the discovery and development of drugs against these diseases have been reviewed previously [12,13,14,15]. In addition, it has been described how the existence of strains resistant to drugs in clinical use for both pathologies does not facilitate their eradication, which emphasizes the need for new drug options for their treatment.

**Figure 4:**
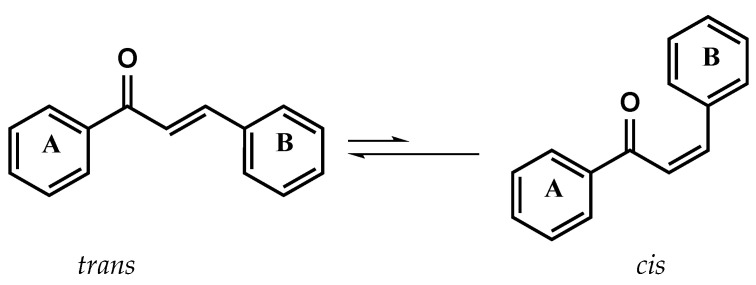
Structures of chalcone *trans* and *cis* isomers.

Since the antimalarial activity of licochalcone A (Figure 3), a natural product isolated from *Chinese licorice* roots, has been reported, several research groups around the world have isolated from natural sources and synthesized a wide variety of chalcone derivatives to study their chemotherapeutic activities and modify their pharmacokinetic properties [16,17,18,19,20,21,22,23,24,25,26,27]. Chalcone compounds have a common chemical scaffold of 1,3-diaryl-2-propen-1-one, which exists as *trans* and *cis* isomers, with the *trans* isomer being thermodynamically more stable (Figure 4); it can act as a Michael acceptor and inhibit the active site due to its ability to form covalent bonds in some receptors containing a thiol group, such as cysteine proteases or trypanothione reductase.

**Figure 5:**
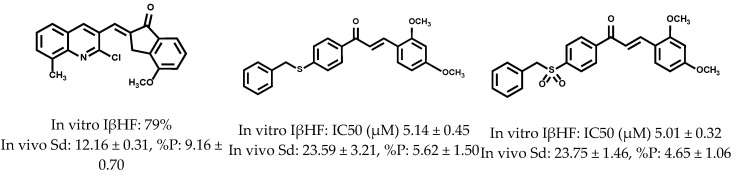
Structures of quino-linylbenzocycloalkanones, 4-benzylsulfanyl, and 4-benzylsulfonyl chalcones actives in vitro and in vivo as antimalarials.

Reports indicate that the chalcones can exert parasiticide activity through their action on several targets, namely: by inhibition of falcipain-2 [28,29,30], sorbitol-induced hemolysis [31], protein kinases (Pfmrk and PfPK5) [32], topoisomerase-II [33], β-hematin [21,22,34], plasmepsin-II, [35] cruzaine, [36] TcGAPDH, [37,38], and Trypanothione reductase [36,37,38]. As part of our program focused on the discovery of new molecules with potential antiparasitic activity, we have recently designed and synthesized a series of quinolinylbenzocycloalkanones, 4-benzylsulfanyl, and 4-benzylsulfonyl chalcones (Figure 5), where some of them were excellent candidates as antimalarials [21,22,39].

Based on these results, we infer that the rigidization of the carbonyl group in these structures favors a cooperative effect necessary to improve the activity during the inhibition of the formation of β-hematin, as well as in the decrease in the parasitemia of infected mice in the rodent malaria model. In addition, disubstitution with methoxyl groups in the indanone ring appears to play an important role in both antiparasitic activity and selectivity. Inspired by these encouraging results, and because of their simple synthetic procedure, we report the synthesis of 4-benzylsulfanyl and 4-benzyloxybenzocycloalkanone derivatives, their antimalarial evaluation in vitro as inhibitors of β-hematin formation and in vivo against a CQ sensitive strain of *P. berghei*, and trypanocidal activity in vitro against epimastigotes of *T. cruzi*.

## 2. Results and Discussion

### 2.1. Synthesis of 4-Benzylsulfanyl and 4-Benzyloxybenzocycloalcanone Derivatives ***7***–***39***

**Scheme 1:**
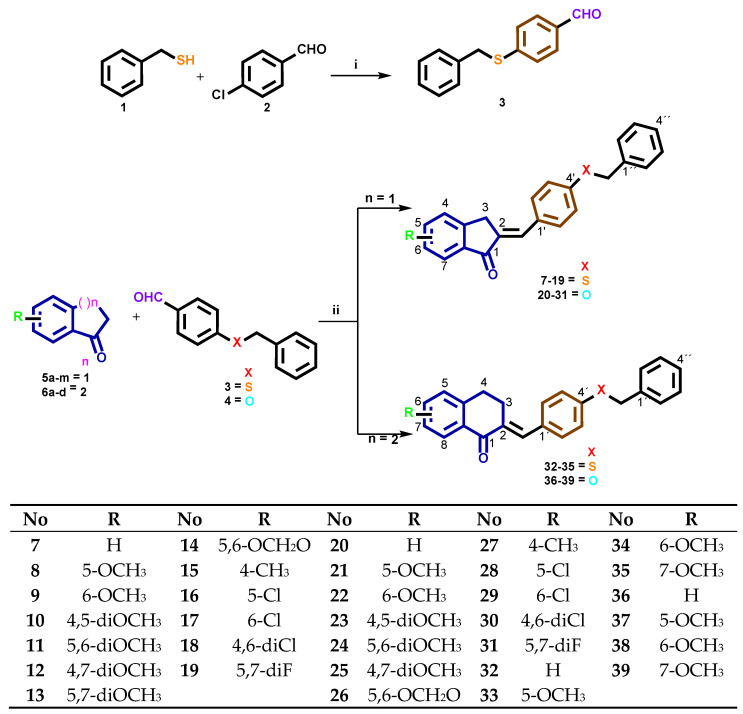
Benzylsulfanyl and 4-benzyloxybenzocycloalcanone derivatives **7**–**39**. **i**: Ethanol, KOH, Δ, 24 h; **ii**: Ethanol, NaOH, rt, 24 h.

The synthesis of derivatives **7**–**39** is mentioned in (Scheme 1) through a facile synthesis in one and two steps. Compound **3** was prepared according to the published procedures [39]. The most important change in the design of these derivatives is the addition of benzyl mercaptan or benzyloxy moieties to position 4 of benzaldehyde. We designed this inclusion because we wanted to evaluate the antimalarial and trypanocidal activities of these compounds with sulfur or oxygen atoms. The final compounds **7**–**39** were synthesized through aldol condensation of Claisen-Schmidt between **3** or **4** and several substituted 1-indanones **5a**–**m** and 1-tetralones **6a**–**d**, using sodium hydroxide as a catalyst in ethanol at room temperature (rt). These conditions were found to be satisfactory for the synthesis in good yields. Only (*E*) isomers were obtained, which were confirmed by singlets (*s*) or triplets (*t*) in the ^1^H-NMR spectra for indanone core between 7.37 and 7.69 or between 7.76 and 7.90 ppm for tetralone core with coupling constants (*J*) around 1.5–1.7 Hz, and confirmed by the COSY experiment. Singlets or doublets (*d*) were around 3.80 and 4.06 ppm *J* = 1.5 Hz for H3 at the indanone core and *t* around 2.90 and 3.12 ppm *J* = 6.5 Hz for H3 at the tetralone core, whereas protons of the –SCH_2_– or –OCH_2_– groups appear as an *s* between 4.12 and 5.19 ppm in each compound, respectively. Protons of the –OCH_3_ group appear as a *s* between 3.83 and 3.91 ppm in each compound; the remaining aromatic protons are reported according to the substitution pattern. The ^13^C NMR spectrum of the same compounds exhibits signals between 135–140 ppm for Cβ and 186–194 ppm for C=O, which were also confirmed by DEPT 135° and 2D NMR experiments as HETCOR, HMQC, or HMBC (Supplementary Materials). The infrared (IR) spectra of the compounds show one characteristic intense stretching band between 1698 and 1679 cm^−1^ for carbonyl present in the 1-indanone ring and between 1648 and 1667 cm^−1^ for carbonyl in 1-tetralone, confirming the presence of α,β unsaturated C=O. For compounds with a sulfanyl group, a characteristic signal is observed for these functional groups between 1310 and 1460 cm^−1^. The analytical data for all compounds are summarized in the experimental section.

### 2.2. Antiprotozoal Activity

The novel synthesized compounds **7**–**39** were tested in vitro as inhibitors of β-hematin formation, and in vivo in a murine model (see Table 1) [39,40]. Most of the compounds evaluated (**7**–**9** and **13**–**39**) exhibited inhibition percentages of less than 50%, with a concentration of 10 μM. There were three compounds **10**, **11**, and **12** that reduced heme crystallization to 75.16%, 63.02%, and 56.17%, respectively, with an IC_50_ 33.1 ± 2.5, 0.82 ± 0.04, and 0.66 ± 0.12 µM. The values are comparable to CQ 95.34% with an IC_50_ value of 0.18 ± 0.03 µM. Compounds with a percentage greater than 50% inhibition of β-hematin formation in vitro were tested in vivo in mice infected with *P. berghei* ANKA, a chloroquine-susceptible strain of murine malaria. The antimalarial potential of these compounds was assessed by their ability to reduce parasitemia and increase survival on the fourth day post-infection compared to the untreated control group.

**Figure 6:**
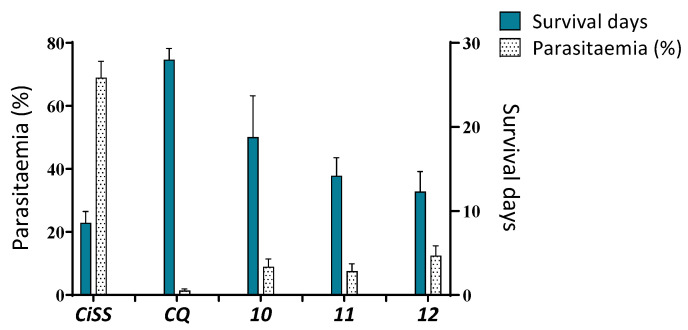
Effects of compounds **10**, **11**, and **12** on parasitemia and survival in male Balb-C mice experimentally infected with *P. berghei*. Right Y axis: % of parasitemia on the fourth-day post-infection. Left Y axis: Survival time post-treatment (days). CiSS = Control infected and treated with saline solution. CQ = Chloroquine.

Mice were treated ip once daily with the test compounds (20 mg kg^−^^1^) or CQ (20 mg kg^−^^1^) for consecutive days (days 1–4 post-infection). Figure 6 shows the survival times and percentage of parasitemia on day four compared with those of control mice receiving only saline solution [41]. The Institute of Immunology Bioethical Committee approved the study according to universal guidelines of the National Research Council’s Institute for Laboratory Animal Research (ILAR) and the ethical principles for medical research by the World Medical Association Declaration of Helsinki. Structures **10**, **11**, and **12** used as a single therapy extended the average survival time of infected mice to 18.8 ± 4.91, 14.2 ± 2.16, and 12.3 ± 2.40 days, respectively; however, they were not able to decrease or delay the evolution of malaria (9.1 ± 2.44, 7.6 ± 2.3, and 12.5 ± 3.1%), respectively. CQ prolonged mouse survival time to 28 ± 1.34 days and decreased the development of malaria to 1.4 ± 0.54%.

**Figure 7:**
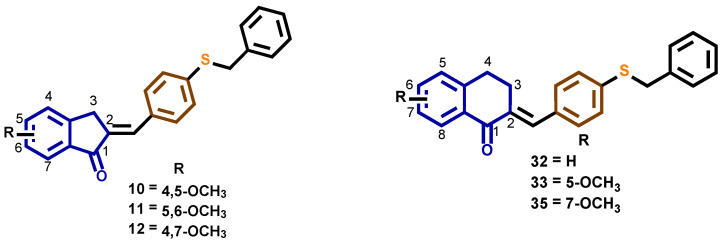
Structures of compounds **10**, **11**, **12**, **32**, **33**, and **35**.

As can be seen in Table 1 and Figure 7, the benzylthio compounds **10**, **11**, and **12** had the best in vitro and in vivo activity as potential antimalarial agents, while the benzyloxy derivatives showed weak activity in both tests. As an antimalarial, the incorporation of a 1-tetralone group decreased activity when compared with 1-indanone. The presence of a withdrawing electron substituent group -Cl, -F, or the mono substitution with -CH_3_, -OCH_3_ at 1-indanone core, also resulted in decreased activity. When these results are compared with those previously reported by our group, no significant changes in antimalarial activity are observed [21,22,39].

In the other assay, compounds **7**–**39** were tested in vitro against epimastigotes of *T. cruzi* (Y strain). Anti-*T. cruzi* properties were expressed in terms of % inhibition of the proliferation of *T. cruzi* epimastigotes for each compound at concentrations of 50 µM after 96 h of parasite exposition. Six compounds, **10**, **11**, **12**, **32**, **33**, and **35**, showed the best results (Table 2). The results described above represent the first report that benzocycloalcanone derivatives had in vitro activity against the parasitic protozoan *T. cruzi*; however, none of the new analogs showed improved activity compared to benznidazole used as a reference drug, except compound (E)-2-[4-(benzylthio)benzylidene]-3,4-dihydro-5-methoxynaphthalen-1(2H)-one **33**, which showed a % of inhibition of proliferation of 51.08 ± 3.4, compared with benznidazole 59.99 ± 2.9%. A low cytotoxicity against VERO cells was observed for **10**, **11**, **19**, **32**, **33**, and **35** (IC_50_ > 150 μM), while benznidazole presents a IC_50_ > 200 μM. In general terms, compounds with sulfur in position 4 of the benzylidene ring, accompanied by a fragment of tetralone, are better candidates as inhibitors of protozoan *T. cruzi* proliferation.

## 3. Materials and Methods

Melting points were determined on a Thomas micro hot stage apparatus and are uncorrected. Thin-layer chromatography (TLC) was carried out on Merck^TM^ silica F254 0.255-mm plates, and spots were visualized by UV fluorescence at 254 nm. IR spectra were determined by a Perkin-Elmer^TM^ Spectrum two spectrophotometer and are expressed in cm**^−^**^1^. The ^1^H and ^13^C NMR spectra were performed using a spectrometer JEOL Eclipse^TM^ 270 (at 270 MHz for ^1^H and 67.9 MHz for ^13^C spectra) or on a Bruker ^TM^ DRX-500 Avance spectrometer (at 500 MHz for ^1^H and 125 MHz for ^13^C spectra), using CDCl_3_ as the solvent, and are reported in ppm downfield from the residual CHCl_3_ (δ 7.25 for ^1^H NMR and 77.0 for ^13^C NMR, respectively). Elemental analyses were achieved using a Perkin Elmer^TM^ 2400 CHN elemental analyzer, and the results were within ± 0.4% of the predicted values. Chemical reagents were obtained from Aldrich Chemical Co^TM^, St. Louis, MO, USA. All solvents were distilled and dried in the usual manner. The 4-(Benzyloxy)benzaldehyde 4 was purchased from Merck^TM^.

### 3.1. Synthesis of 4-(Benzylthio)benzaldehyde 3

A mixture of benzylmercaptan 1 (2.73 g, 22 mmol) and potassium hydroxide (1.23 g, 22 mmol) in 95% ethanol (10 mL) was heated to reflux until KOH had completely dissolved and was cooled to room temperature. 4-chlorobenzaldehyde 2 (2.81 g, 20 mmol) dissolved in 95% ethanol was then added dropwise. The solution was heated to reflux for 24 h. When cooled, a yellow solid precipitate formed, which was filtered and washed with ethanol and water. The solid was dissolved in ether, washed with water and 1N NaOH aqueous solution, dried with MgSO_4_, and the solvent removed to give a white solid. Yield: 76%; Mp: 62–64 °C; IR (ZnSe) cm**^−^**^1^: 2800, 1683, 1574; ^1^H NMR (CDCl_3_, 270 MHz) δ ppm: 4.23 (s, 2H, CH_2_), 7.24–7.38 (m, 7H, Ar), 7.73(d, 2H, H_2,6_, *J* = 8.7 Hz), 9.90 (s, 1H, CHO); ^13^C NMR (CDCl_3_, 67.9 MHz) δ ppm: 37.0, 126.9, 127.7, 128.8, 130.1, 133.6, 136.0, 146.4, 191.3. Anal. Calcd for C_14_H_12_OS: C, 73.65; H, 5.30; S, 14.04. Found: C, 73.66; H, 5.29; S, 14.10.

### 3.2. General Procedure for the Synthesis of (E)-2-[4-(Benzylthio)benzylidene or (E)-2-[4-(Benzyloxy)benzylidene]-Benzocycloalkanone Derivatives ***7***–***39***

A mixture of 4-(benzylthio)benzaldehyde 3 or 4-(benzyloxy)benzaldehyde 4 (0.43 mmol), 1-indanone or 1-tetralone respective (0.43 mmol), and sodium hydroxide one pellet in ethanol 95% (5 mL) was stirred at room temperature for 24 h. The resulting precipitate was collected by filtration, washed with cold water, sodium bisulfite 10% aqueous solution, diethyl ether, and recrystallized from ethanol-water (9:1).

#### 3.2.1. (E)-2-[4-(Benzylthio)benzylidene]-2,3-dihydroinden-1-one **7**

Yield 82%; m.p. 155–157 °C; IR (ZnSe) cm^−1^: 1682, 1629, 1571, 1495, 1327, 1273, 1229; ^1^H NMR (CDCl_3_, 270 MHz) δ ppm: 4.00 (d, 2H, H_3_, *J* = 1.7 Hz), 4.19 (s, 2H, PhCH_2_), 7.26–7.44 (m, 7H, Art), 7.52–7.64 (m, 6H, are, Hv), 7.88 (d, 1H, H_7_, *J* = 7.7 Hz); ^13^C NMR (CDCl_3_, 67.9 MHz) *δ* ppm: 32.6, 37.9, 124.5, 126.2, 127.5, 127.8, 128.4, 128.7, 128.8, 131.2, 132.9, 133.4, 134.3, 134.7, 136.7, 138.1, 139.7, 149.5, 194.4. Anal. Calca for C_23_H_18_OS: C, 80.67; H, 5.30; S, 9.36. Found: C, 80.89; H, 5.28; S, 9.30.

#### 3.2.2. (E)-2-[4-(Benzylthio)benzylidene]-2,3-dihydro-5-methoxyinden-1-one **8**

Yield 79%; m.p. 166–168 °C; IR (ZnSe) cm^−1^: 1682, 1622, 1602, 1580, 1487, 1337, 1254; ^1^H NMR (CDCl_3_, 270 MHz) δ ppm: 3.89 (s, 3H, OCH_3_), 3.94 (d, 2H, H_3_, *J* = 1.5 Hz), 4.18 (s, 2H, PhCH_2_), 6.93 (dd, 1H, H_6_, *J* = 8.3, 2.2 Hz), 7.26–7.34 (m, 8H, Ar), 7.51 (d, 2H, H_2′,6′_, *J* = 8.2 Hz), 7.53 (s, 1H, Hv), 7.82 (d, 1H, H_7_, *J* = 8.4 Hz); ^13^C NMR (CDCl_3_, 67.9 MHz) δ ppm: 32.6, 38.0, 55.8, 109.8, 115.3, 126.2, 127.7, 128.4, 128.7, 128.9, 131.0, 131.5, 132.1, 133.1, 134.9, 136.8, 139.3, 152.4, 165.3, 192.8. Anal. Calce for C_24_H_20_O_2_S: C, 77.39; H, 5.41; S, 8.61. Found: C, 77.43; H, 5.45; S, 8.72.

#### 3.2.3. (E)-2-[4-(Benzylthio)benzylidene]-2,3-dihydro-6-methoxyinden-1-one **9**

Yield 72%; m.p. 175–177 °C; IR (ZnSe) cm^−1^: 1684, 1617, 1584, 1489, 1282, 1257; ^1^H NMR (CDCl_3_, 270 MHz) δ ppm: 3.85 (s, 3H, OCH_3_), 3.92 (d, 2H, H_3_, *J* = 1.5 Hz), 4.19 (s, 2H, PhCH_2_), 7.18 (dd, 1H, H_5_, *J* = 8.4, 2.5 Hz), 7.26–7.32 (m, 8H, Ar), 7.42 (d, 1H, H_4_, *J* = 8.4 Hz), 7.53 (d, 2H, H_2′, 6′_, *J* = 8.5 Hz), 7.57 (t, 1H, Hv, *J* = 1.5 Hz); ^13^C NMR (CDCl_3_, 67.9 MHz) δ ppm: 31.9, 37.9, 55.7, 105.8, 124.0, 126.9, 127.5, 128.3, 128.8, 128.9, 131.1, 132.9, 133.3, 135.2, 136.7, 139.4, 139.7, 142.4, 159.7, 194.3. Anal. Calca for C_24_H_20_O_2_S: C, 77.39; H, 5.41; S, 8.61. Found: C, 77.41; H, 5.42; S, 8.47.

#### 3.2.4. (E)-2-[4-(Benzylthio)benzylidene]-2,3-dihydro-4,5-dimethoxyinden-1-one **10**

Yield 79%; m.p. 173–175 °C; IR (ZnSe) cm^−1^: 1685, 1617, 1584, 1493, 1340, 1280, 1269; ^1^H NMR (CDCl_3_, 270 MHz) δ ppm: 3.96 (s, 5H, OCH_3_, H_3_), 3.97 (s, 3H, OCH_3_), 4.19 (s, 2H, PhCH_2_), 7.01 (d, 1H, H_6_, *J* = 8.4 Hz), 7.26–7.37 (m, 7H, Ar), 7.54 (s, 1H, Hv), 7.56 (d, 2H, H_2′,6′_, *J* = 8.6 HZ), 7.67 (d, 1H, H_7_, *J* = 8.4 Hz); ^13^C NMR (CDCl_3_, 67.9 MHz) δ ppm: 29.5, 37.9, 56.3, 60.7, 112.6, 121.2, 127.5, 128.4, 128.7, 128.8, 131.1, 132.2, 132.7, 132.9, 134.7, 136.8, 139.5, 142.4, 145.2, 157.7, 192.9. Anal. Calce for C_25_H_22_O_3_S: C, 74.60; H, 5.51; S, 7.97. Found: C, 74.65; H, 5.53; S, 8.05.

#### 3.2.5. (E)-2-[4-(Benzylthio)benzylidene]-2,3-dihydro-5,6-dimethoxyinden-1-one **11**

Yield 81%; m.p. 160–164 °C; IR (ZnSe) cm^−1^: 1681, 1625, 1585, 1496, 1303, 1275, 1224; ^1^H NMR (CDCl_3_, 270 MHz) δ ppm: 3.91 (d, 2H, H_3_, *J* = 1.7 Hz), 3.93 (s, 3H, OCH_3_), 3.98 (s, 3H, OCH_3_), 4.18 (s, 2H, PhCH_2_), 6.96 (s, 1H, H_4_), 7.25–7.33 (m, 7H, Ar), 7.50–754 (m, 4H, Ar, Hv); ^13^C NMR (CDCl_3_, 67.9 MHz) δ ppm: 32.3, 37.9, 56.3, 56.4, 105.1, 107.2, 127.5, 128.5, 128.7, 128.9, 130.9, 131.2, 131.9, 133.1, 135.1, 136.8, 139.2, 144.8, 149.8, 155.5, 193.1. Anal. Calca for C_25_H_22_O_3_S: C, 74.60; H, 5.51; S, 7.97. Found: C, 74.61; H, 5.56; S, 8.12.

#### 3.2.6. (E)-2-[4-(Benzylthio)benzylidene]-2,3-dihydro-4,7-dimethoxyinden-1-one **12**

Yield 68%; m.p. 205–207 °C; IR (ZnSe) cm^−1^: 1694, 1628, 1587, 1492, 1283, 1265; ^1^H NMR (CDCl_3_, 270 MHz) δ ppm: 3.83 (d, 2H, H_3_, *J* = 1.3 Hz), 3.87 (s, 3H, OCH_3_), 3.92 (s, 1H, OCH_3_), 4.17 (s, 2H, PhCH_2_), 6.76 (d, 1H, H_5_, *J* = 8.9 Hz), 7.01 (d, 1H, H_6_, *J* = 8.9 Hz), 7.26–7.33 (m, 7H, Ar), 7.52 (s, 1H, Hv), 7.54 (d, 2H, H_2′,6′_, *J* = 8.4 Hz); ^13^C NMR (CDCl_3_, 67.9 MHz) δ ppm: 29.3, 37.9, 55.9, 56.2, 110.1, 116.7, 127.3, 127.5, 128.5, 128.7, 128.9, 131.1, 132.5, 133.0, 134.4, 136.8, 139.2, 139.8, 150.0, 152.5, 192.4. Anal. Calce for C_25_H_22_O_3_S: C, 74.60; H, 5.51; S, 7.97. Found: C, 74.71; H, 5.51; S, 7.85.

#### 3.2.7. (E)-2-[4-(Benzylthio)benzylidene]-2,3-dihydro-5,7-dimethoxyinden-1-one **13**

Yield 87%; m.p.176–178 °C; IR (ZnSe) cm^−1^: 1683, 1629, 1585, 1487, 1324, 1275, 1229; ^1^H NMR (CDCl_3_, 270 MHz) δ ppm: 3.87 (s, 5H, OCH_3_, H_3_), 3.92 (s, 3H, OCH_3_), 4.17 (s, 2H, PhCH_2_), 6.31 (s, 1H, H_6_), 6.53 (s, 1H, H_4_), 7.23–7.34 (m, 7H, Ar), 7.44 (t, 1H, Hv, *J* = 1.7 Hz), 7.47 (d, 2H, H_2′,6′_, *J* = 8.4 Hz); ^13^C NMR (CDCl_3_, 67.9 MHz) δ ppm: 32.8, 38.1, 55.9, 56.0, 97.7, 101.6, 120.7, 127.5, 128.6, 128.7, 128.9, 130.8, 133.4, 135.2, 136.9, 138.7, 154.4, 160.2, 167.0, 190.7. Anal. Calca for C_25_H_22_O_3_S: C, 74.60; H, 5.51; S, 7.97. Found: C, 74.60; H, 5.54; S, 8.09.

#### 3.2.8. (E)-2-[4-(Benzylthio)benzylidene]-2,3-dihydro-5,6-methylendioxyinden-1-one **14**

Yield 80%; m.p.194–196 °C; IR (ZnSe) cm^−1^: 1687, 1631, 1593, 1513, 1465, 1294, 1041; ^1^H NMR (CDCl_3_, 270 MHz) δ ppm: 3.86 (s, 2H, H_3_), 4.17 (s, 2H, PhCH_2_), 6.06 (s, 2H, OCH_2_O), 6.89 (s, 1H, H_4_), 7.23–7.35 (m, 8H, Ar), 7.49 (m, 3H, Ar, Hv); ^13^C NMR (CDCl_3_, 67.9 MHz) δ ppm: 32.5, 38.0, 102.3, 103.3, 103.4, 105.5, 127.5, 128.5, 128.7, 128.8, 131.0, 132.0, 133.0, 134.9, 136.8, 139.4, 146.9, 147.2, 154.1, 192.5. Anal. Calce for C_24_H_18_O_3_S: C, 74.59; H, 4.69; S, 8.30. Found: C, 74.62; H, 4.73; S, 8.43.

#### 3.2.9. (E)-2-[4-(Benzylthio)benzylidene]-2,3-dihydro-4-methylinden-1-one **15**

Yield 87; m.p. 167–169 °C; IR (ZnSe) cm^−1^: 1670, 1637, 1576, 1478, 1247, 830; ^1^H NMR (CDCl_3_, 270 MHz) δ ppm: 2.42 (s, 3H, CH_3_), 3.84 (s, 2H, H_3_), 4.19 (s, 2H, PhCH_2_), 7.25–7.42 (m, 9H, Ar), 7.55 (d, 2H, H_2′,6′_, *J* = 8.4 Hz), 7.59 (t, 1H, Hv, *J* = 1.5 Hz), 7.73 (d, 1H, H_7_, *J* = 7.5 Hz); ^13^C NMR (CDCl_3_, 67.9 MHz) δ ppm: 18.1, 31.3, 37.9, 121.9, 127.5, 127.9, 128.4, 128.7, 128.8, 131.1, 132.9, 133.3, 134.4, 135.3, 135.4, 136.7, 137.9, 139.7, 148.5, 194.7. Anal. Calca for C_24_H_20_OS: C, 80.86; H, 5.65; S, 8.99. Found: C, 80.90; H, 5.67; S, 8.95.

#### 3.2.10. (E)-2-[4-(Benzylthio)benzylidene]-5-chloro-2,3-dihydroinden-1-one **16**

Yield 80%; m.p. 140–142 °C; IR (ZnSe) cm^−1^: 1692, 1627, 1600, 1469, 1320, 1262, 1068, 810; ^1^H NMR (CDCl_3_, 500 MHz) δ ppm: 4.06 (d, 2H, H_3_, *J* = 1.8 Hz), 4.26 (s, 2H, PhCH_2_), 7.05 (s, 1H, H_4_), 7.14–7.34 (m, 7H, Ar), 7.56 (dd, 1H, H_6_, *J* = 8.0, 2.1 Hz), 7.68 (d, 2H, H_2′,6′_, *J* = 8.2 Hz), 7.69 (s, 1H, Hv), 7.89 (d, 1H, H_7_, *J* = 8.0 Hz); ^13^C NMR (CDCl_3_, 125.7 MHz) δ ppm: 33.2, 38.1, 124.7, 126.6, 126.8, 127.8, 128.6, 128.7, 128.8, 130.6, 131.9, 137.0, 137.1, 138.4, 139.2, 147.8, 192.4. Anal. Calce for C_23_H_17_ClOS: C, 73.29; H, 4.55; S, 8.51. Found: C, 73.15; H, 4.58; S, 8.33.

#### 3.2.11. (E)-2-[4-(Benzylthio)benzylidene]-6-chloro-2,3-dihydroinden-1-one **17**

Yield 67%; m.p. 204 °C; IR (ZnSe) cm^−1^: 1687, 1620, 1586, 1494, 1257, 1092, 812; ^1^H NMR (CDCl_3_, 500 MHz) δ ppm: 4.02 (d, 2H, H_3_, *J* = 1.5 Hz), 4.27 (s, 2H, PhCH_2_), 7.22–7.25 (m, 2H, H_4,4″_), 7.33–7.44 (m, 7H, Ar), 7.56–7.67 (m, 3H, H_v,_ Ar), 7.91 (d, 1H, H_7_, *J* = 2.4 Hz); ^13^C NMR (CDCl_3_, 125.7 MHz) δ: 31.9, 38.1, 124.9, 127.8, 128.1, 128.6, 128.7, 128.9, 130.7, 131.3, 132.0, 132.9, 137.0, 136.8, 138.1, 138.2, 139.7, 145.4, 196.3; Anal. Calca for C_23_H_17_ClOS: C, 73.29; H, 4.55; S, 8.51. Found: C, 73.38; H, 4.52; S, 8.60.

#### 3.2.12. (E)-2-[4-(Benzylthio)benzylidene]-4,6-dichloro-2,3-dihydroinden-1-one **18**

Yield 73%; m.p. 181–183 °C; IR (ZnSe) cm^−1^: 1695, 1614, 1584, 1495, 1312, 1269, 1093; ^1^H NMR (CDCl_3_, 500 MHz) δ ppm: 3.99 (d, 2H, H_3_, *J* = 2.0 Hz), 4.28 (s, 2H, PhCH_2_), 7.15 (m, 1H, H_4″_), 7.32–7.46 (m, 7H, Ar), 7.58–7.67 (m, 2H, Ar), 7.68 (s, 1H, Hv), 7.66 (d, 1H, H_7_, *J* = 2.0 Hz); ^13^C NMR (CDCl_3_, 125.7 MHz) δ ppm: 32.4, 38.7, 124.7, 127.8, 128.6, 128.7, 128.8, 130.1, 131.2, 132.3, 132.5, 132.8, 133.1, 137.1, 137.6, 138.3, 138.9, 143.0, 193.4; Anal. Calce for C_23_H_16_Cl_2_OS: C, 67.16; H, 3.92; S, 7.80. Found: C, 66.97; H, 4.11; S, 8.03.

#### 3.2.13. (E)-2-[4-(Benzylthio)benzylidene]-5,7-difluoro-2,3-dihydroinden-1-one **19**

Yield 77%; m.p. 170–172 °C; IR (ZnSe) cm^−1^: 1693, 1618, 1592, 1431, 1327, 1256, 847; ^1^H NMR (CDCl_3_, 270 MHz) δ ppm: 3.99 (d, 2H, H_3_, *J* = 1.5 Hz), 4.20 (s, 2H, PhCH_2_), 6.79 (t, 1H, H_6_, *J* = 7.9 Hz), 7.01 (d, 1H, H_4_, *J* = 7.9 Hz), 7.27–7.36 (m, 7H, Ar), 7.50 (d, 2H, H_2′,6′_, *J* = 8.4 Hz), 7.57 (t, 1H, Hv, *J* = 1.5 Hz); ^13^C NMR (CDCl_3_, 67.9 MHz) δ ppm: 32.8, 37.8, 104.0 (q, *J*: 24 Hz), 109.3 (d, *J*: 25 Hz), 127.5, 128.3, 128.8, 128.9, 131.1, 132.3, 133.1, 133.8, 136.6, 140.3, 153.3 (d, *J*: 12 Hz), 189.3. Anal. Calca for C_23_H_16_F_2_OS: C, 73.00; H, 4.26; S, 8.47. Found: C, 72.97; H, 4.23; S, 8.24.

#### 3.2.14. (E)-2-[4-(Benzyloxy)benzylidene]-2,3-dihydroinden-1-one **20**

Yield 79%; m.p. 170 °C; IR (ZnSe) cm^−1^: 1679, 1628, 1596, 1509, 1424, 1251, 955, 821; ^1^H NMR (CDCl_3_, 500 MHz) δ ppm: 3.97 (s, 2H, H_3_), 5.11 (s, 2H, PhCH_2_), 7.04 (d, 2H, H_2′,6′_, *J* = 8.5 Hz), 7.34 (t, 1H, H_4″_, *J* = 5.0 Hz), 7.39–7.45 (m, 5H, Ar), 7.53 (d, 1H, H_4_, *J* = 7.5 Hz), 7.59 (t, 1H, H_5_, *J* = 7.5 Hz), 7.62 (d, 2H, H_3′,5′_, *J* = 8.5 Hz), 7.37 (t, 1H, Hv, *J* = 1.7 Hz), 7.88 (d, 1H, H_7_, *J* = 7.5 Hz); ^13^C NMR (CDCl_3_, 125.7 MHz) δ ppm: 32.5, 70.2, 115.4, 124.3, 126.1, 127.4, 127.6, 128.2, 128.5, 128.7, 132.5, 132.6, 133.7, 134.3, 136.5, 138.3, 160.1, 194.3. Anal. Calcd for C_23_H_18_O_2_: C, 84.64; H, 5.56; O, 9.80. Found: C, 84.69; H, 5.59; O, 9.57.

#### 3.2.15. (E)-2-[4-(Benzyloxy)benzylidene]-2,3-dihydro-5-methoxyinden-1-one **21**

Yield 91%; m.p. 141–142 °C; IR (ZnSe) cm^−1^: 1682, 1627, 1598, 1509, 1291, 1247, 821, ^1^H NMR (CDCl_3_, 500 MHz) δ ppm: 4.05 (s, 5H, OCH_3_, H_3_), 5.10 (s, 2H, PhCH_2_), 7.05 (s, 1H, H_4_), 7.26–7.34 (m, 8H, Ar), 7.53 (d, 2H, H_2′,6′_, *J* = 8.2 Hz), 7.75 (s, 1H, Hv), 7.84 (d, 1H, H_7_, *J* = 8.4 Hz); ^13^C NMR (CDCl_3_, 125.7 MHz) δ ppm: 32.6, 37.3, 69.9, 107.4, 115.2, 122.4, 126.7, 127.1, 128.3, 128.7, 129.1, 131.9, 133.7, 133.9, 136.3, 140.3, 142.4, 159.0, 161.1, 194.2. Anal. Calcd for C_24_H_20_O_3_: C, 80.88; H, 5.66; O, 13.47. Found: C, 81.01; H, 5.65; O, 13.53.

#### 3.2.16. (E)-2-[4-(Benzyloxy)benzylidene]-2,3-dihydro-6-methoxyinden-1-one **22**

Yield 87%; m.p. 178–179 °C; IR (ZnSe) cm^−1^: 3078, 1680, 1620, 1598, 1566, 1508, 1489, 1277, 819; ^1^H NMR (CDCl_3_, 500 MHz) δ ppm: 3.85 (s, 3H, OCH_3_), 3.90 (s, 2H, H_3_), 5.12 (s, 2H, PhCH_2_), 7.03 (d, 2H, H_2′,6′_, *J* = 8.0 Hz), 7.17 (d, 1H, H_4_, *J* = 8.0 Hz), 7.34–7.45 (m, 8H, Ar), 7.60–7.62 (m, 2H, H_v,7_); ^13^C NMR (CDCl_3_, 125.7 MHz) δ ppm: 31.8, 35.6, 70.1, 105.8, 115.4, 123.6, 126.8, 127.5, 128.2, 128.5, 128.7, 132.5, 133.5, 133.6, 136.5, 139.5, 142.3, 159.6, 160.0, 194.3. Anal. Calcd for C_24_H_20_O_3_: C, 80.88; H, 5.66; O, 13.47. Found: C, 80.87; H, 5.67; O, 13.51.

#### 3.2.17. (E)-2-[4-(Benzyloxy)benzylidene]-2,3-dihydro-4,5-dimethoxyinden-1-one **23**

Yield 89%; m.p. 167–168 °C; IR (ZnSe) cm^−1^: 1688, 1628, 1598, 1508, 1494, 1338, 1275, 1046; ^1^H NMR (CDCl_3_, 500 MHz) δ ppm: 3.93 (d, 2H, CH_2_, *J* = 1.5 Hz), 3.95 (s, 3H, OCH_3_), 3.97 (s, 3H, OCH_3_), 5.10 (s, 2H, PhCH_2_), 6.99 (d, 1H, H_6_, *J* = 8.5 Hz), 7.04 (d, 2H, H_2′,6′_, *J* = 8.5 Hz), 7.33 (t, 1H, H_4″_, *J* = 7.5 Hz), 7.38–7.44 (m, 4H, Ar), 7.56 (t, 1H, Hv, *J* = 1.5 Hz), 7.62 (d, 2H, H_3′,5′_, *J* = 8.5 Hz), 7.65 (d, 1H, H_7_, *J* = 8.5 Hz); ^13^C NMR (CDCl_3_, 125.7 MHz) δ ppm: 29.4, 56.2, 60,5, 70.1, 112.5, 115.4, 120.9, 127.5, 128.2, 128.5, 128.7, 132.4, 132.5, 132.9, 133.0, 136.5, 142.4, 145.2, 157.4, 159.9, 192.9. Anal. Calcd for C_25_H_22_O_4_: C, 77.70; H, 5.74; O, 16.56. Found: C, 77.68; H, 5.73; O, 16.73.

#### 3.2.18. (E)-2-[4-(Benzyloxy)benzylidene]-2,3-dihydro-5,6-dimethoxyinden-1-one **24**

Yield 73%; m.p. 207–208 °C; IR (ZnSe) cm^−1^: 2941, 1688, 1628, 1598, 1508, 1484, 1275, 1242, 813; ^1^H NMR (CDCl_3_, 500 MHz) δ ppm: 3.82 (s, 2H, H_3_,), 3.89 (s, 3H, OCH_3_), 3.94 (s, 3H, OCH_3_), 5.07 (s, 2H, PhCH_2_), 6.91 (d, 1H, H_4_), 6.99 (d, 2H, H_2′,6′_, *J* = 8.0 Hz), 7.32 (t, 1H, H_4″_, *J* = 7.0 Hz), 7.36–7.42 (m, 4H, Ar), 7.49 (s, 1H, Hv), 7.53 (m, 3H, H_3′,5′,7′_); ^13^C NMR (CDCl_3_, 125.7 MHz) δ ppm: 32.2, 56.1, 56.2, 70.1, 105.1, 107.2, 115.3, 127.5, 128.2, 128.6, 128.7, 131.3, 132.2, 132.3, 133.3, 136.5, 144.7, 149.6, 155.3, 159.8, 193.2. Anal. Calcd for C_25_H_22_O_4_: C, 77.70; H, 5.74; O, 16.56. Found: C, 77.74; H, 5.79; O, 16.69.

#### 3.2.19. (E)-2-[4-(Benzyloxy)benzylidene]-2,3-dihydro-4,7-dimethoxyinden-1-one **25**

Yield 83%; m.p. 209–210 °C; IR (ZnSe) cm^−1^: 1694, 1633, 1598, 1494, 1241, 1061, 1006, 981; ^1^H NMR (CDCl_3_, 500 MHz) δ ppm: 3.80 (d, 2H, H_3_, *J* = 1.5 Hz), 3.86 (s, 3H, OCH_3_), 3.92 (s, 3H, OCH_3_), 5.09 (s, 2H, PhCH_2_), 6.75 (d, 1H, H_5_, *J* = 8.5 Hz), 6.97 (d, 1H, H6, *J* = 8.5 Hz), 7.01 (d, 2H, H_2′,6′_, *J* = 8.5 Hz), 7.33 (t, 1H, H_4″_, *J* = 7.0 Hz), 7.37–7.43 (m, 4H, Ar), 7.54 (t, 1H, Hv, *J* = 1.5 Hz), 7.61 (d, 2H, H_3′,5′_); ^13^C NMR (CDCl_3_, 125.7 MHz) δ ppm: 29.2, 55.9, 56.1, 70.1, 110.1, 115.3, 116.5, 127.5, 128.1, 128.6, 128.7, 132.4, 132.7, 132.8, 136.5, 139.8, 150.1, 152.4, 159.8, 192.4. Anal. Calcd for C_25_H_22_O_4_: C, 77.70; H, 5.74; O, 16.56. Found: C, 77.81; H, 5.73; O, 16.80.

#### 3.2.20. (E)-2-[4-(Benzyloxy)benzylidene]-2,3-dihydro-5,6-methylendioxyinden-1-one **26**

Yield 79%; m.p. 184–185 °C; IR (ZnSe) cm^−1^: 3103, 1681, 1629, 1597, 1509, 1465, 1294, 1249, 825; ^1^H NMR (CDCl_3_, 500 MHz) δ ppm: 3.92 (d, 2H, H_3_, *J* = 1.2 Hz), 5.18 (s, 2H, PhCH_2_), 6.13 (s, 2H, OCH_2_O), 6.97 (s, 1H, H_4_), 7.02 (d, 2H, H_3′,5′_, *J* = 8.5 Hz), 7.32–7.53 (m, 7H, Ar), 7.61 (s, 1H, H_7_), 7.69 (s, 1H, Hv); ^13^C NMR (CDCl_3_, 125.7 MHz) δ ppm: 35.2, 69.8, 102.4, 106.1, 106.5, 115.1, 127.7, 127.9, 128.2, 128.3, 132.3, 132.4, 136.1, 137.0, 139.4, 141.1, 147.9, 150.2, 160.3, 193.0. Anal. Calcd for C_25_H_22_O_4_: C, 77.82; H, 4.90; O, 17.28. Found: C, 77.73; H, 5.07; O, 17.04.

#### 3.2.21. (E)-2-[4-(Benzyloxy)benzylidene]-2,3-dihydro-4-methylinden-1-one **27**

Yield 81%; m.p. 127–129 °C; IR (ZnSe) cm^−1^: 1688, 1630, 1598, 1508, 1278, 1249, 830, 744; ^1^H NMR (CDCl_3_, 500 MHz) δ ppm: 2.43 (s, 3H, CH_3_), 3.82 (s, 2H, H_3_), 5.12 (s, 2H, PhCH_2_), 7.05 (d, 2H, H_2′,6′_, *J* = 9.0 Hz), 7.30–7.36 (m, 3H, H_4″,5,6_), 7.39–7.35 (m, 4H, Ar), 7.62–7.64 (m, 3H, H_v,3′,5′_), 7.73 (d, 1H, H_7_, *J* = 7.5 Hz); ^13^C NMR (CDCl_3_, 125.7 MHz) δ ppm: 17.9, 31.2, 70.1, 115.4, 121.7, 127.5, 127.8, 128.2, 128.5, 128.7, 132.5, 132.7, 133.6, 134.9, 135.2, 136.5, 138.0, 148.5, 160.0, 194.7. Anal. Calcd for C_24_H_20_O_2_: C, 84.68; H, 5.92; O, 9.40. Found: C, 84.69; H, 5.97; O, 9.53.

#### 3.2.22. (E)-2-[4-(Benzyloxy)benzylidene]-5-chloro-2,3-dihydroinden-1-one **28**

Yield 85%; m.p. 189–190 °C; IR (ZnSe) cm^−1^: 1693, 1633, 1599, 1494, 1321, 1249, 1175, 999, 826; ^1^H NMR (CDCl_3_, 500 MHz) δ ppm: 4.04 (s, 2H, H_3_), 5.19 (s, 2H, PhCH_2_), 7.11 (d, 2H, H_3′,5′_, *J* = 8.0 Hz), 7.13 (s, 1H, H_4_), 7.38–7.50 (m, 7H, Ar), 7.58–7.72 (m, 2H, H_v,6_), 7.88 (d, 1H, H_7_, *J* = 7.5 Hz); ^13^C NMR (CDCl_3_, 125.7 MHz) δ ppm: 31.8, 69.8, 115.3, 124.5, 126.6, 126.8, 127.7, 127.9, 128.1, 128.5, 132.1, 136.2, 136.9, 137.6, 137.9, 139.7, 147.9, 160.1, 194.8. Anal. Calcd for C_23_H_17_ClO_2_: C, 76.56; H, 4.75; O, 8.87. Found: C, 76.59; H, 4.72; O, 9.04.

#### 3.2.23. (E)-2-[4-(Benzyloxy)benzylidene]-6-chloro-2,3-dihydroinden-1-one **29**

Yield 77%; m.p. 186–187 °C; IR (ZnSe) cm^−1^: 3065, 1690, 1622, 1596, 1509, 1463, 1254, 988; ^1^H NMR (CDCl_3_, 500 MHz) δ ppm: 3.97 (d, 2H, H_3_, *J* = 2.0 Hz), 5.08 (s, 2H, PhCH_2_), 7.26–7.36 (m, 8H, Ar), 7.53 (d, 2H, H_3′,5′_, *J* = 8.6 Hz), 7.56 (dd, 1H, H_5_, *J* = 8.0, 2.1 Hz), 7.62 (t, 1H, Hv, *J* = 2.0 Hz), 7.86 (d, 1H, H_7_, *J* = 2.1 Hz); ^13^C NMR (CDCl_3_, 125.7 MHz) δ ppm: 31.3, 70.2, 115.6, 122.7, 127.5, 128.4, 128.8, 128.7, 131.0, 132.9, 133.0, 133.5, 135.8, 136.3, 141.1, 145.5, 160.6, 192.0; Anal. Calce for C_23_H_17_ClO_2_: C, 76.56; H, 4.75; O, 8.87. Found: C, 76.43; H, 4.70; O, 8.97.

#### 3.2.24. (E)-2-[4-(Benzyloxy)benzylidene]-4,6-dichloro-2,3-dihydroinden-1-one **30**

Yield 67%; m.p. 202–203 °C; IR (ZnSe) cm^−1^: 1698, 1621, 1595, 1451, 1246, 833, 741; ^1^H NMR (CDCl_3_, 500 MHz) δ ppm: 3.92 (s, 2H, H_3_), 5.14 (s, 2H, PhCH_2_), 7.07 (d, 2H, H_2′,6′_, *J* = 8.5 Hz), 7.35 (t, 1H, H_4″_, *J* = 7.5 Hz), 7.39–7.45 (m, 4H, Ar), 7.58 (d, 1H, H_5_, *J* = 2.0 Hz), 7.64 (d, 2H, H_3′,5′_, *J* = 8.5 Hz), 7.68 (s, 1H, Hv), 7.66 (d, 1H, H_7_, *J* = 2.0 Hz); ^13^C NMR (CDCl_3_, 125.7 MHz) δ ppm: 31.3, 70.2, 115.6, 122.7, 127.5, 127.8, 128.2, 128.7, 131.0, 132.9, 133.0, 133.5, 134.8, 135.8, 136.3, 141.1, 145.5, 160.6, 192.0. Anal. Calcd for C_23_H_16_Cl_2_O_2_: C, 69.89; H, 4.08; O, 8.10. Found: C, 70.05; H, 4.11; O, 8.25.

#### 3.2.25. (E)-2-[4-(Benzyloxy)benzylidene]-5,7-difluoro-2,3-dihydroinden-1-one **31**

Yield 67%; m.p. 204–206 °C; IR (ZnSe) cm^−1^: 1690, 1621, 1594, 1256, 847, 827, 697; ^1^H NMR (CDCl_3_, 500 MHz) δ ppm: 4.05 (d, 2H, H_3,_ *J* = 1.5 Hz), 5.14 (s, 2H, PhCH_2_), 6.78 (td, 1H, H_4_, *J* = 9.0, 2.0 Hz), 7.01 (dd, 1H, H_6_, *J* = 9.0, 2.0 Hz), 7.05 (d, 2H, H_2′,6′_, *J* = 9.0 Hz), 7.36 (t, 1H, H_4_, *J* = 7.5 Hz), 7.39–7.45 (m, 4H, Ar), 7.59 (d, 2H, H_2′,6′_, *J* = 9.0 Hz), 7.61 (t, 1H, Hv, *J* = 1.5 Hz); ^13^C NMR (CDCl_3_, 125.7 MHz) δ ppm: 32.7, 70.2, 103.7 (q, *J* = 23 Hz), 109.1 (d, *J* = 17.6 Hz), 115.5, 127.5, 128.0, 128.2, 128.7, 131.4, 132.6, 134.1, 136.4, 136.6, 153.3, 160.3, 163.6, 189.4. Anal. Calcd for C_23_H_16_F_2_O_2_: C, 76.23; H, 4.45; O, 8.83. Found: C, 76.31; H, 4.47; O, 8.92.

#### 3.2.26. (E)-2-[4-(Benzylthio)benzylidene]-3,4-dihydronaphthalen-1(2H)-one **32**

Yield 82%; m.p. 99–100 °C; IR (ZnSe) cm^−1^: 3018, 1656, 1608, 1584, 1512, 1439, 1260, 893; ^1^H NMR (CDCl_3_, 270 MHz) δ ppm: 2.92 (t, 2H, H_4_, *J* = 6.7 Hz), 3.09 (t, 2H, H_3_, *J* = 6.5 Hz), 4.17 (s, 2H, PhCH_2_), 7.22–7.35 (m, 11H, Ar), 7.48 (t, 1H, Ar, *J* = 7.4 Hz), 7.79 (s, 1H, Hv), 8.11 (d, 1H, H_8_, *J* = 7.67 Hz); ^13^C NMR (CDCl_3_, 67.9 MHz) δ ppm: 27.4, 28.9, 38.3, 127.1, 127.5, 128.2, 128.3, 128.5, 128.7, 128.9, 130.5, 133.4, 133.5, 133.6, 135.3, 136.2, 137.0, 138.0, 143.2, 187.8. Anal. Calca for C_24_H_20_OS: C, 80.86; H, 5.65; S, 8.99. Found: C, 81.01; H, 5.66; S, 9.18.

#### 3.2.27. (E)-2-[4-(Benzylthio)benzylidene]-3,4-dihydro-5-methoxynaphthalen-1(2H)-one **33**

Yield 74%; m.p. 87–89 °C; IR (ZnSe) cm^−1^: 3119, 1661, 1612, 1563, 1490, 1265, 831; ^1^H NMR (CDCl_3_, 270 MHz) δ ppm: 2.90 (t, 2H, H_4_, *J* = 6.4 Hz), 3.05 (t, 2H, H_3_, *J* = 6.0 Hz), 3.85 (s, 3H, OCH_3_), 4.17 (s, 2H, PhCH_2_), 7.03 (dd, 1H, H_5_, *J* = 8.2, 1.2 Hz), 7.25–7.33 (m, 9H, Ar), 7.73 (dd, 1H, H_8_, *J* = 8.2, 1.2 Hz), 7.75 (s, 1H, Hv); ^13^C NMR (CDCl_3_, 67.9 MHz) δ ppm: 21.5, 26.7, 38.3, 55.8, 114.4, 120.0, 127.3, 127.5, 128.5, 128.7, 128.9, 130.5, 132.3, 133.6, 134.5, 135.3, 135.9, 137.0, 137.9, 156.4, 188.1. Anal. Calce for C_25_H_22_O_2_S: C, 77.69; H, 5.74; S, 8.30. Found: C, 77.73; H, 5.77; S, 8.26.

#### 3.2.28. (E)-2-[4-(Benzylthio)benzylidene]-3,4-dihydro-6-methoxynaphthalen-1(2H)-one **34**

Yield 68%; m.p. 122–124 °C; IR (ZnSe) cm^−1^: 3137, 1651, 1610, 1590, 1435, 1301, 1260; ^1^H NMR (CDCl_3_, 270 MHz) δ ppm: 2.89 (t, 2H, H_4_, *J* = 6.9 Hz), 3.07 (t, 2H, H_3_, *J* = 6.7 Hz), 3.86 (s, 3H, OCH_3_) 4.16 (s, 2H, PhCH_2_), 6.69 (d, 1H, H_5_, *J* = 2.5 Hz), 6.86 (dd, 1H, H_7_, *J* = 8.6, 2.5 Hz), 7.27–7.33 (m, 9H, Ar), 7.76 (t, 1H, Hv, 1.5 Hz), 8.09 (d, 1H, H_8_, *J* = 8.6 Hz); ^13^C NMR (CDCl_3_, 67.9 MHz) δ: 27.4, 29.3, 38.4, 55.6, 112.3, 113.4, 127.1, 127.4, 128.6, 128.7, 128.9, 130.4, 130.9, 133.7, 135.4, 135.5, 137.0, 137.7, 145.7, 163.7, 186.7. Anal. Calca for C_25_H_22_O_2_S: C, 77.69; H, 5.74; S, 8.30. Found: C, 77.70; H, 5.75; S, 8.41.

#### 3.2.29. (E)-2-[4-(Benzylthio)benzylidene]-3,4-dihydro-7-methoxynaphthalen-1(2H)-one **35**

Yield 76%; m.p. 112–115 °C; IR (ZnSe) cm^−1^: 3100, 1648, 1604, 1571, 1437, 1329, 1293, 825; ^1^H NMR (CDCl_3_, 270 MHz) δ ppm: 2.86 (t, 2H, H_4_, *J* = 7.0 Hz), 3.07 (t, 2H, H_3_, *J* = 6.8 Hz), 3.85 (s, 3H, OCH_3_) 4.16 (s, 2H, PhCH_2_), 7.04 (dd, 1H, H_6_, *J* = 8.4, 2.7 Hz), 7.14 (d, 1H, H_5_, *J* = 8.4 Hz), 7.24–7.35 (m, 9H, Ar), 7.59 (d, 1H, H_8_, *J* = 2.7 Hz), 7.77 (t, 1H, Hv, *J* = 1.5 Hz); ^13^C NMR (CDCl_3_, 67.9 MHz) δ ppm: 27.6, 28.0, 38.3, 55.7, 110.4, 121.6, 127.5, 128.5, 128.7, 128.9, 129.5, 130.5, 133.5, 134.3, 135.3, 135.9, 136.3, 137.0, 158.7, 187.8. Anal. Calce for C_25_H_22_O_2_S: C, 77.69; H, 5.74; S, 8.30. Found: C, 77.90; H, 5.79; S, 8.53.

#### 3.2.30. (E)-2-[4-(Benzyloxy)benzylidene]-3,4-dihydronaphthalen-1(2H)-one **36**

Yield 66%; oil; IR (ZnSe) cm^−1^: 3117, 1661, 1610, 1584, 1523, 1487, 1265, 841; ^1^H NMR (CDCl_3_, 270 MHz) δ ppm: 2.72 (t, 2H, H_4_, *J* = 6.5 Hz), 3.03 (t, 2H, H_3_, *J* = 6.5 Hz), 5.21 (s, 2H, PhCH_2_), 7.03 (d, 2H, H_2′6′_, *J* = 8.5 Hz), 7.14 (d, 1H, H_5_, *J* = 8.5 Hz), 7.32–7.53 (m, 9H, Ar), 7.81 (s, 1H, Hv), 8.14 (dd, 1H, H_8_, *J* = 8.5, 2.0 Hz); ^13^C NMR (CDCl_3_, 67.9 MHz) δ ppm: 27.2, 28.8, 70.1, 110.6, 115.0, 127.1, 127.2, 128.0, 128.2, 128.4, 128.5, 131.7, 133.1, 133.6, 136.2, 136.7, 139.0, 143.1, 160.1, 189.4. Anal. Calcd for C_24_H_20_O_2_: C, 84.68; H, 5.92; O, 9.40. Found: C, 84.90; H, 5.87; O, 9.63.

#### 3.2.31. (E)-2-[4-(Benzyloxy)benzylidene]-3,4-dihydro-5-methoxynaphthalen-1(2H)-one **37**

Yield 47%; m.p. 67–69 °C; IR (ZnSe) cm^−1^: 3087, 1667, 1608, 1577, 1512, 1493, 1267, 829; ^1^H NMR (CDCl_3_, 270 MHz) δ ppm: 2.70 (t, 2H, H_4_, *J* = 6.2 Hz), 2.97 (t, 2H, H_3_, *J* = 6.0 Hz), 3.93 (s, 3H, OCH_3_), 5.22 (s, 2H, PhCH_2_), 7.04–7.19 (m, 7H, Ar), 7.26–7.41 (m, 3H, Ar), 7.77 (dd, 1H, H_6_, *J* = 8.4, 2.0 Hz), 7.90 (m, 2H, H_8_, Hv); ^13^C NMR (CDCl_3_, 67.9 MHz) δ ppm: 27.4, 28.6, 55.4, 69.8, 110.7, 114.1, 120.6, 127.9, 128.0, 128.2, 128.5, 130.4, 131.7, 133.7, 136.1, 136.2, 138.2, 139.7, 157.2, 160.1, 189.3. Anal. Calcd for C_25_H_22_O_3_: C, 81.06; H, 5.99; O, 12.96: Found: C, 80.92; H, 5.97; O, 13.03.

#### 3.2.32. (E)-2-[4-(Benzyloxy)benzylidene]-3,4-dihydro-6-methoxynaphthalen-1(2H)-one **38**

Yield 59%; m.p. 130–131 °C; IR (ZnSe) cm^−1^: 3109, 1665, 1600, 1579, 1510, 1270, 1142, 830; ^1^H NMR (CDCl_3_, 500 MHz) δ ppm: 2.91 (t, 2H, H_4_, *J* = 6.5 Hz), 3.12 (t, 2H, H_3_, *J* = 6.5 Hz), 3.87 (s, 3H, OCH_3_), 5.11 (s, 2H, PhCH_2_), 6.70 (d, 1H, H_5_, *J* = 2.0 Hz), 6.87 (dd, 1H, H_7_, *J* = 8.5, 2.0 Hz), 7.01 (d, 2H, H_2′,6′_, *J* = 8.5 Hz), 7.37 (t, 1H, H_4″_, *J* = 7.5 Hz), 7.38–7.45 (m, 6H, Ar), 7.81 (s, 1H, Hv), 8.11 (d, 1H, H_8_, *J* = 8.5 Hz); ^13^C NMR (CDCl_3_, 125.7 MHz) δ ppm: 27.3, 29.2, 55.5, 70.1, 112.3, 113.3, 114.8, 127.2, 127.5, 128.1, 128.7, 128.8, 130.7, 131.7, 133.8, 135.9, 136.7, 145.6, 159.0, 163.5, 186.8. Anal. Calcd for C_25_H_22_O_3_: C, 81.06; H, 5.99; O, 12.96. Found: C, 80.93; H, 5.95; O, 13.16.

#### 3.2.33. (E)-2-[4-(Benzyloxy)benzylidene]-3,4-dihydro-7-methoxynaphthalen-1(2H)-one **39**

Yield 71%; m.p. 119–120 °C; IR (ZnSe) cm^−1^: 3121, 1662, 1603, 1582, 1508, 1495, 1254, 1286, 830; ^1^H NMR (CDCl_3_, 500 MHz) δ ppm: 2.89 (t, 2H, H_4_, *J* = 6.5 Hz), 3.12 (t, 2H, H_3_, *J* = 6.0 Hz), 3.87 (s, 3H, OCH_3_), 5.12 (s, 2H, PhCH_2_), 7.02 (d, 1H, H_5_, *J* = 8.5 Hz), 7.06 (dd, 2H, H_6_, *J* = 8.5, 2.0 Hz), 7.15 (d, 2H, H_2′,6′_, *J* = 8.0 Hz), 7.34 (t, 1H, H_4″_, *J* = 7.0 Hz), 7.39–7.45 (m, 6H, Ar), 7.62 (d, 1H, H_8_, *J* = 2.0 Hz), 7.83 (s, 1H, Hv); ^13^C NMR (CDCl_3_, 125.7 MHz) δ ppm: 27.4, 27.9, 55.6, 70.1, 110.4, 114.9, 121.3, 127.5, 128.1, 128.6, 128.7, 129.3, 131.7, 133.7, 134.5, 135.8, 136.7, 158.7, 159.2, 187.8. Anal. Calca for C_25_H_22_O_3_: C, 81.06; H, 5.99; O, 12.96. Found: C, 81.12; H, 6.05; O, 13.21.

### 3.3. Biology

#### 3.3.1. Inhibition of β-Haematin Formation In Vitro

The assay was performed according to previously reported protocols [39,40]. Hemin chloride (50 μL, 4 mM) in DMSO (5.2 mg mL^−1^) was pipetted into 96-well microplates. Different concentrations (0.1–100 mM) of the compounds in DMSO were added in triplicate (50 μL) with a final concentration of 0.025 mM–25 mM/well. Water (50 μL) or DMSO (50 μL) were used as controls. Acetate buffer (100 μL, 0.2 M, pH 4.4) initiated the formation of β-hematin. Next, the plates were incubated at 37 °C for 48 h and subsequently centrifuged (4000 rpm × 15 min). The supernatant was washed twice with DMSO (200 μL) and resuspended in NaOH (200 μL, 0.2 N). The solubilized products were diluted (1:2) with NaOH (0.1 N), and the plates were read in an ELISA reader at 405 nm (Microplate Reader, BIORAD-550). The results are expressed as % IβHF.

#### 3.3.2. Plasmodium Berghei and Experimental Host Maintenance

Male Balb-C mice with a weight of 18–22 g were fed with a commercial diet ad libitum under standard procedures of animal care following the aforementioned method approved by the Ethics Committee of the Institute of Immunology. The animals were infected with a rodent malaria strain of *Plasmodium berghei* ANKA. A million infected erythrocytes, in phosphate-buffered saline solution (PBS, 10 mM, pH 7.4, 0.1 mL), were inoculated ip to infect the animal. The parasitemia was inspected by continuous microscopic examination of Giemsa-stained smears [39].

#### 3.3.3. Four-Day Suppressive Test

One million *P. berghei*, injected in the caudal vein *i.v*, were used to infect the mice (n = 6). After two hours of inoculation, the compounds that were active in vitro were dissolved in DMSO (0.1 M) and subsequently diluted with Saline-Tween 20 solution (2%), and administered *ip* for 4 days (20 mg kg**^−^**^1^). The parasite load was evaluated on the fourth day by examining Giemsa-stained smears. Chloroquine (20 mg kg**^−^**^1^) was used as the positive control and saline solution as the negative control. Non-treated mice were used as a baseline control for survival times [39,41]. The survival days and percentage of parasitemia were used to express the results.

#### 3.3.4. Trypanocidal Activity

The trypanocidal activity of compounds 7–39 was evaluated on the viability of *T. cruzi* epimastigotes (Y Strain). Parasites were cultivated in LIT medium supplemented with 10% FBS. The MTT method was used with minor modifications [42]. Next, 2 × 10^6^ parasites/mL were seeded in a 96-well plate, adding 50 μM of each derivative dissolved in DMSO (final concentration remained below 1%). The plate was incubated for 96 h at 29 °C. Then, 5 mg mL**^−^**^1^ of 3-(4,5-dimethylthiazol-2-yl)-2,5-diphenyl tetrazolium bromide (MTT) was added and incubated in darkness for 4 h. After this time, acidic isopropanol (4N) was added and the plate was read at 540 nm in a spectrophotometer Synergy HT (Biotek). Benznidazole (50 µM) was used as a reference drug.

#### 3.3.5. Cytotoxicity Assay

The in vitro evaluations of compounds toxicity by MTT in VERO cells were performed as described in brief: VERO cells culture in DMEM, BSF 10%, were counted in suspension, seeded at 2 × 10^4^ cells/well (100 μL) into a 96-well microplate, and incubated at 37 °C in a humidified 5% CO_2_ incubator for 24 h. After the incubation period, compounds were added in different concentrations: 50, 100, 150, 200, and 500 μM. The plate was incubated for 72 h in the same conditions. Then, 5 mg mL**^−^**^1^ of 3-(4,5-dimethylthiazol-2-yl)-2,5-diphenyl tetrazolium bromide (MTT) was added and incubated in darkness for 4 h. After this time, DMSO was added and the plate was read at 540 nm in a spectrophotometer Synergy HT (Biotek). The test was carried out in triplicate including untreated cells and reference drug controls [43].

## 4. Conclusions

In summary, we synthesized 33 compounds derived from benzocycloalkanones with final yields ranging from moderate to very good through a synthesis strategy that was very useful and feasible. The compounds were characterized by spectroscopic techniques. Only three compounds, **10**, **11**, and **12**, showed an inhibitory effect greater than 50% on the formation of β-hematin in vitro. The results of the in vivo antimalarial evaluation show that **10**, **11**, and **12** reduced parasitemia marginally, and an insignificant increase in the days of survival of the mice was observed after the fourth day of infection. As trypanocidals, all benzocycloalkanones showed marginal activity as inhibitors of the proliferation of *T. cruzi* epimastigotes, except compound **33**, which had an activity of 51.08 ± 3.4% compared to the activity shown by the reference compound benznidazole 59.99 ± 2.9%. The compounds appear to have little cytotoxic effect on VERO cells when compared to the value presented by benznidazole against these mammalian cells. In general, we can infer that benzylthiobenzocycloalkanone compounds would be candidates as possible antiprotozoal agents, but the best activity could be achieved when tetralone rings are incorporated, which would make them ideal hits for further optimization.

## Figures and Tables

**Table 1:** The half maximal inhibitory concentrations (IC_50_ values) of compounds **7**–**39** to inhibit the formation of β-hematin (βHF), effects on *P. berghei*-infected mice (20 mg kg^−1^).

No	%IβHF (± SEM) ^a^	IC_50_(µM) (± SEM) ^b^	Sd(± SEM) ^c^	%P(± SEM) ^d^	Survival
**10**	75.16	33.1 ± 2.5	18.8 ± 4.9 *	9.1 ± 2.44	0/6
**11**	63.02	0.82 ± 0.1	14.2 ± 2.2 *	7.6 ± 2.3	0/6
**12**	56.17	0.66 ± 0.1	12.3 ± 2.4	12.5 ± 3.1	0/6
**CQ**	95.34	0.18 ± 0.1	28 ± 1.3	1.4 ± 0.54	5/6
**CiSS**	-	-	8.6 ± 1.3	69.9 ± 5.17	0/6

^a^ %IβHF: Percentage inhibition β-hematin formation, compounds **7**–**9**, **13**–**31, 34** and **36**–**39** IβHF < 50%; ^b^ IC_50_: Inhibitory concentration 50 (βHF) (n = 3). ^c^ Sd: Survival days. ^d^ %P: Percentage of parasitemia. SEM = Standard error of the mean. CQ = chloroquine. CiSS = Control infected and treated with saline solution. * *p* < 0.001 compared to CiSS. n = 6.

**Table 2:** Effect of compounds **5**–**37** on the proliferation of epimastigotes of *Trypanosoma cruzi*, and VERO cells.

No	%I*Tc*P (50 µM) ^a^	VERO IC_50_ µM
**10**	32.95 ± 5.6	>150
**11**	39.99 ± 8.2	>150
**12**	18.50 ± 2.9	>150
**32**	43.66 ± 6.7	>150
**33**	51.08 ± 3.4	>150
**35**	36.25 ± 6.1	>150
**Bnz**	59.99 ± 2.9	>200

^a^ *T. cruzi* (epimastigotes) % inhibition proliferation, compounds **5**–**7**, **11**–**29, 32** and **34**–**37** < 30%. Bnz = benznidazole.

## Data Availability

The data presented in this study are contained within the article and Supplementary Materials.

## Supplementary Materials

The following supporting information can be downloaded at: https://www.mdpi.com/article/10.3390/molecules28145569/s1. The following are available: ^1^H/^13^C NMR, DEPT 135°, COSY, HETCOR, HMQC, and HMBC.

## Sample Availability

Samples of the compounds are available from the authors.
