# Supplementary material for: Synthesis of Benzocycloalkanone-Based Michael Acceptors and Biological Activities as Antimalarial and Antitrypanosomal Agents
Source: Molecules. 2023 Jul 21;28(14):5569. doi: 10.3390/molecules28145569 (PMC10385825; doi:10.3390/molecules28145569)

Supplementary Material

Synthesis of Benzocycloalkanone-based Michael Acceptors and Biological Activities as Antimalarial and Antitrypanosomal Agents.

Ali Mijoba [1,2], Esteban Fernandez-Moreira [3], Nereida Parra-Giménez [2], Sandra Espinosa-Tapia [4], Zuleyma Blanco [1], Hegira Ramírez [5], Jaime E. Charris [1]

[1]Organic Synthesis Laboratory, Faculty of Pharmacy, Central University of Venezuela, 47206, Los Chaguara- mos 1041-A, Caracas, Venezuela; alimijoba@gmail.com (AM); blancomzule@gmail.com (ZB)

[2]Laboratory of Parasites Physiology. BioPhysics and Biochemistry Center. Instituto Venezolano de Invest gaciones Científicas. Altos de Pipe, Caracas, Venezuela. bionereida@gmail.com (NPG)

[3]Escuela de Medicina, Universidad Espíritu Santo, Samborondón 092301, Ecuador; estebanfernandez@uees.edu.ec (EFM)

[4]Departamento de Química, Universidad Técnica Particular de Loja, Loja 1101608, Ecuador; sandraespinosa100@hotmail.com (SE)

[5] Facultad de Ciencias de la Salud y Desarrollo Humano, Universidad Ecotec, Km. 13.5 Samborondón, Samborondón, EC092302, Ecuador

Table of Contents

		Page
**S1**	The ^{1}H NMR spectrum of 3	5
**S2**	The ^{13}C NMR spectrum of 3	5
**S3**	The ^{1}H NMR spectrum of 7	6
**S4**	The ^{13}C NMR spectrum of 7	6
**S5**	The ^{1}H NMR spectrum of 8	7
**S6**	The ^{1}H NMR spectrum of 9	8
**S7**	The ^{13}C NMR spectrum of 9	8
**S8**	The DEPT 135° spectrum of 9	9
**S9**	The HETCOR spectrum of 9	9
**S10**	The ^{1}H NMR spectrum of 10	10
**S11**	The ^{13}C NMR spectrum of 10	10
**S12**	The DEPT 135° spectrum of 10	11
**S13**	The HETCOR spectrum of 10	11
**S14**	The ^{1}H NMR spectrum of 11	12
**S15**	The ^{13}C NMR spectrum of 11	12
**S16**	The DEPT 135° spectrum of 11	13
**S17**	The HETCOR spectrum of 11	13
**S18**	The ^{1}H NMR spectrum of 12	14
**S19**	The ^{13}C NMR spectrum of 12	14
**S20**	The DEPT 135° spectrum of 12	15
**S21**	The HETCOR spectrum of 12	15
**S22**	The ^{1}H NMR spectrum of 13	16
**S23**	The ^{13}C NMR spectrum of 13	16
**S24**	The ^{1}H NMR spectrum of 14	17
**S25**	The ^{13}C NMR spectrum of 14	17
**S26**	The COSY spectrum of 14	18
**S27**	The HETCOR spectrum of 14	18
**S28**	The ^{1}H NMR spectrum of 15	19
**S29**	The ^{13}C NMR spectrum of 15	19
**S30**	The ^{1}H NMR spectrum of 16	20
**S31**	The ^{13}C NMR spectrum of 16	20
**S32**	The ^{1}H NMR spectrum 17	21
**S33**	The ^{13}C NMR spectrum of 17	21
**S34**	The ^{1}H NMR spectrum 18	22
**S35**	The ^{13}C NMR spectrum of 18	22
**S36**	The DEPT 135° spectrum of 18	23
**S37**	The ^{1}H NMR spectrum of 19	24
**S38**	The ^{13}C NMR spectrum of 19	24
**S39**	The DEPT 135° spectrum of 19	25
**S40**	The HETCOR spectrum of 19	25
**S41**	The ^{1}H NMR spectrum of 20	26

**S42**	The ^{13}C NMR spectrum of 20	26
**S43**	The COSY spectrum of 20	27
**S44**	The HMQC spectrum of 20	27
**S45**	The HMBC spectrum of 20	28
**S46**	The ^{1}H NMR spectrum of 22	29
**S47**	The ^{13}C NMR spectrum of 22	29
**S48**	The DEPT 135° spectrum of 22	30
**S49**	The COSY spectrum of 22	30
**S50**	The HMQC spectrum of 22	31
**S51**	The HMBC spectrum of 22	31
**S52**	The ^{1}H NMR spectrum of 23	32
**S53**	The ^{13}C NMR spectrum of 23	32
**S54**	The DEPT 135° spectrum of 23	33
**S55**	The COSY spectrum of 23	33
**S56**	The HMQC spectrum of 23	34
**S57**	The HMBC spectrum of 23	34
**S58**	The ^{1}H NMR spectrum of 24	35
**S59**	The ^{13}C NMR spectrum of 24	35
**S60**	The COSY spectrum of 24	36
**S61**	The DEPT 135° spectrum of 24	36
**S62**	The HMQC spectrum of 24	37
**S63**	The HMBC spectrum of 24	37
**S64**	The ^{1}H NMR spectrum of 25	38
**S65**	The ^{13}C NMR spectrum of 25	38
**S66**	The DEPT 135° spectrum of 25	39
**S67**	The COSY spectrum of 25	39
**S68**	The HMQC spectrum of 25	40
**S69**	The HMBC spectrum of 25	40
**S70**	The ^{1}H NMR spectrum of 27	41
**S71**	The ^{13}C NMR spectrum of 27	41
**S72**	The DEPT 135° spectrum of 27	42
**S73**	The COSY spectrum of 27	42
**S74**	The HMQC spectrum of 27	43
**S75**	The HMBC spectrum of 27	43
**S76**	The ^{1}H NMR spectrum of 30	44
**S77**	The ^{13}C NMR spectrum of 30	44
**S78**	The ^{1}H NMR spectrum of 31	45
**S79**	The ^{13}C NMR spectrum of 31	45
**S80**	The DEPT 135° spectrum of 31	46
**S81**	The COSY spectrum of 31	46
**S82**	The HMQC spectrum of 31	47
**S83**	The HMBC spectrum of 31	47
**S84**	The ^{1}H NMR spectrum of 32	48
**S85**	The ^{13}C NMR spectrum of 32	48
**S86**	The DEPT 135° spectrum of 32	49

**S87**	The COSY spectrum of 32	49
**S88**	The HETCOR spectrum of 32	50
**S89**	The ^{1}H NMR spectrum of 33	51
**S90**	The ^{13}C NMR spectrum of 33	51
**S91**	The ^{1}H NMR spectrum of 34	52
**S92**	The ^{13}C NMR spectrum of 34	52
**S93**	The DEPT 135° spectrum of 34	53
**S94**	The HETCOR spectrum of 34	53
**S95**	The ^{1}H NMR spectrum of 35	54
**S96**	The ^{13}C NMR spectrum of 35	54
**S97**	The DEPT 135° spectrum of 35	55
**S98**	The HETCOR spectrum of 35	55
**S99**	The ^{1}H NMR spectrum of 38	56
**S100**	The ^{13}C NMR spectrum of 38	56
**S101**	The DEPT 135° spectrum of 38	57
**S102**	The HMQC spectrum of 38	57
**S103**	The HMBC spectrum of 38	58
**S104**	The ^{1}H NMR spectrum of 39	59
**S105**	The ^{13}C NMR spectrum of 39	59
**S106**	The DEPT 135° spectrum of 39	60
**S107**	The COSY spectrum of 39	60
**S108**	The HMQC spectrum of 39	61
**S109**	The HMBC spectrum of 39	61

S1. Comp. 3

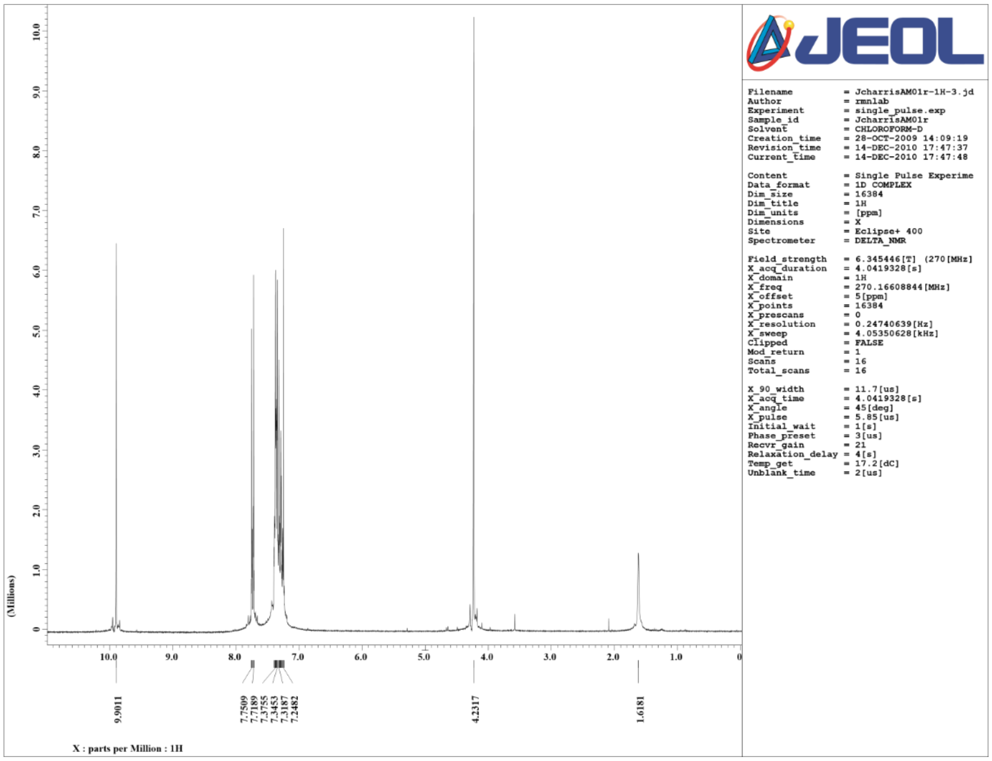

S2. Comp. 3

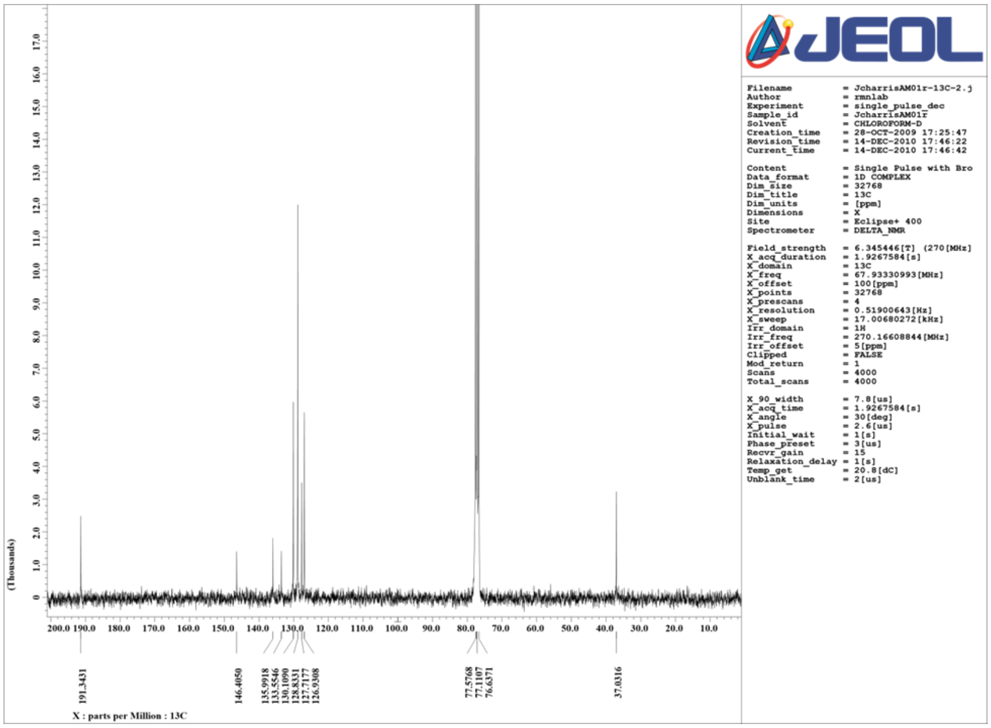

S3. Comp. 7

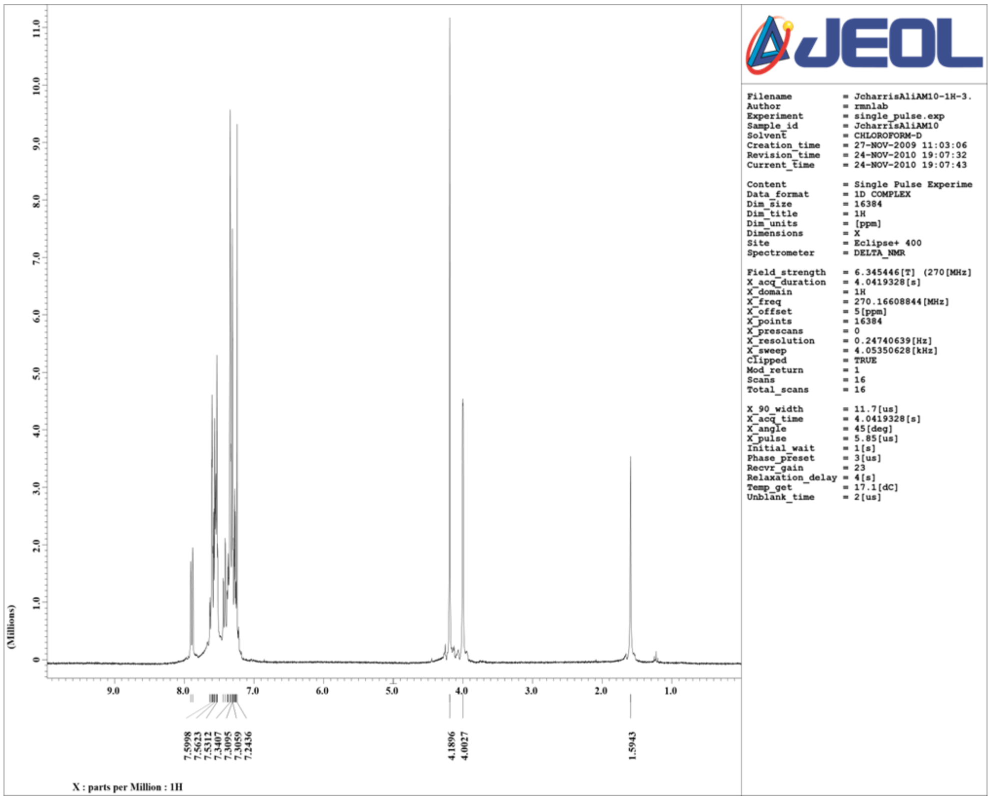

S4. Comp. 7

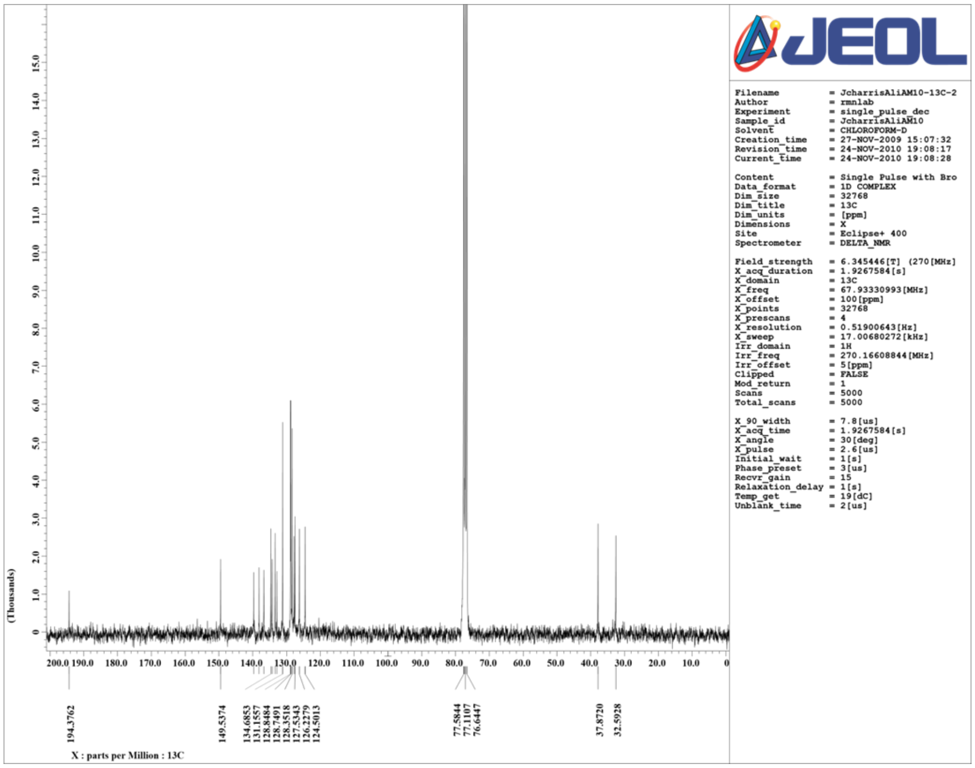

S5. Comp. 8

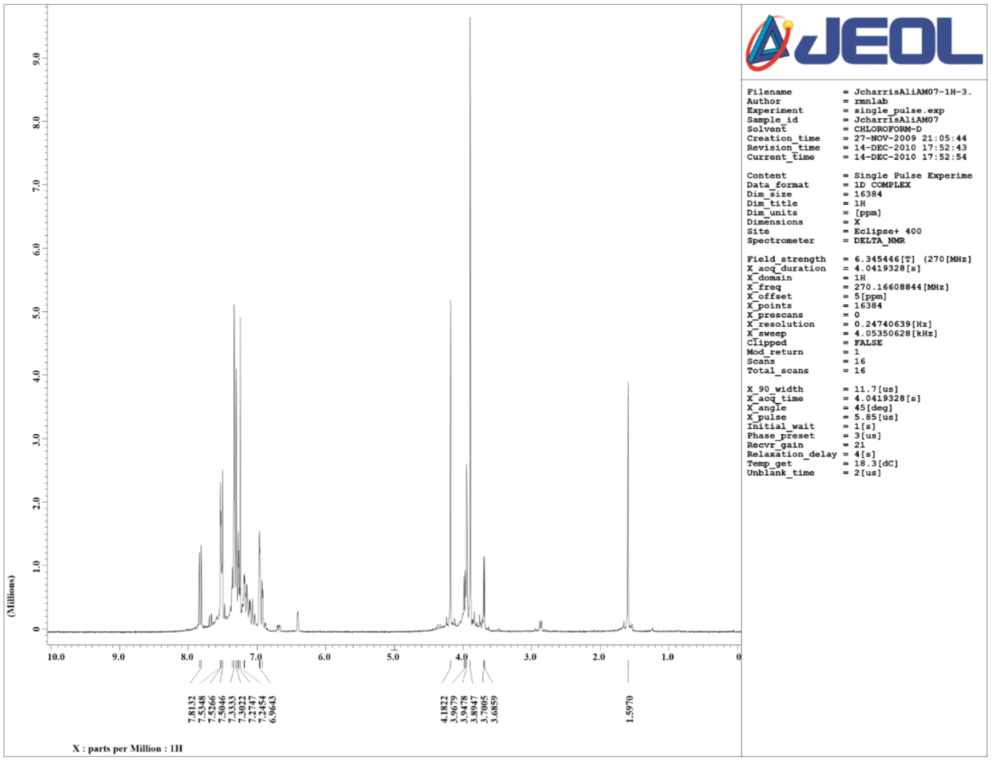
JEOL
Filename = JcharrisAliAM07-1H-3.
Author = rmnlab
Experiment = single_pulse.exp
Sample_id = JcharrisAliAM07
Solvent = CHLOROFORM-D
Creation_time = 27-NOV-2009 21:05:44
Revision_time = 14-DEC-2010 17:52:43
Current_time = 14-DEC-2010 17:52:54
Content = Single Pulse Experime
Data_format = 1D COMPLEX
Dim_size = 16384
Dim_title = 1H
Dim_units = [ppm]
Dimensions = X
Site = Eclipse+ 400
Spectrometer = DELTA_NMR
Field_strength = 6.345446[T] (270[MHz]
X_acq_duration = 4.0419328[s]
X_domain = 1H
X_freq = 270.16608844[MHz]
X_offset = 5[ppm]
X_points = 16384
X_prescans = 0
X_resolution = 0.24740639[Hz]
X_sweep = 4.05350628[kHz]
Clipped = FALSE
Mod_return = 1
Scans = 16
Total_scans = 16
X_90_width = 11.7[us]
X_acq_time = 4.0419328[s]
X_angle = 45[deg]
X_pulse = 5.85[us]
Initial_wait = 1[s]
Phase_preset = 3[us]
Recvr_gain = 21
Relaxation_delay = 4[s]
Temp_get = 18.3[dC]
Unblank_time = 2[us]
(Millions)
7.8132
7.5348
7.5266
7.5046
7.3333
7.3022
7.2747
7.2454
6.9643
4.1822
3.9679
3.9478
3.8947
3.7005
3.6859
1.5970
X : parts per Million : 1H

S6. Comp. 9

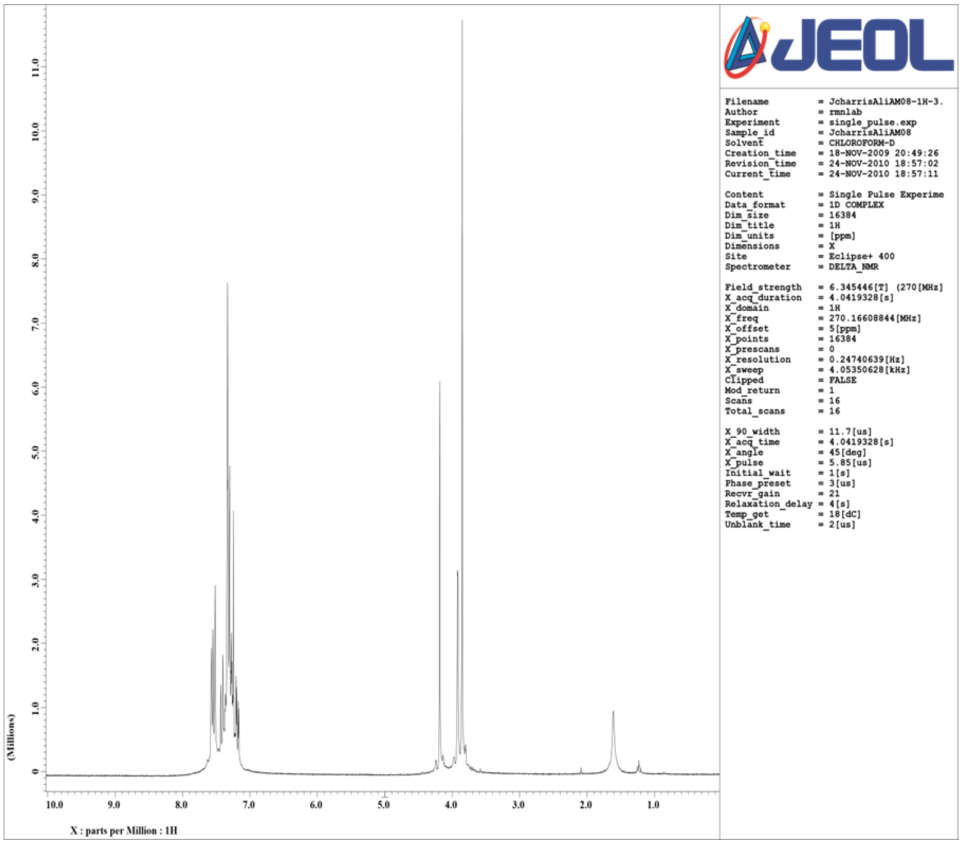

S7. Comp. 9

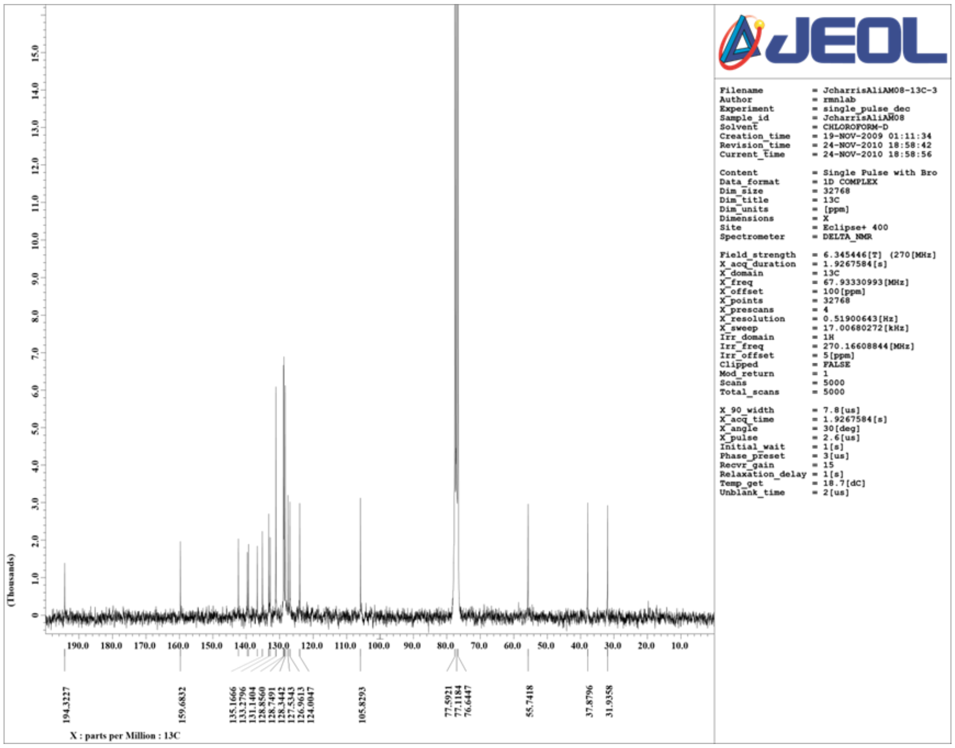

S8. Comp. 9

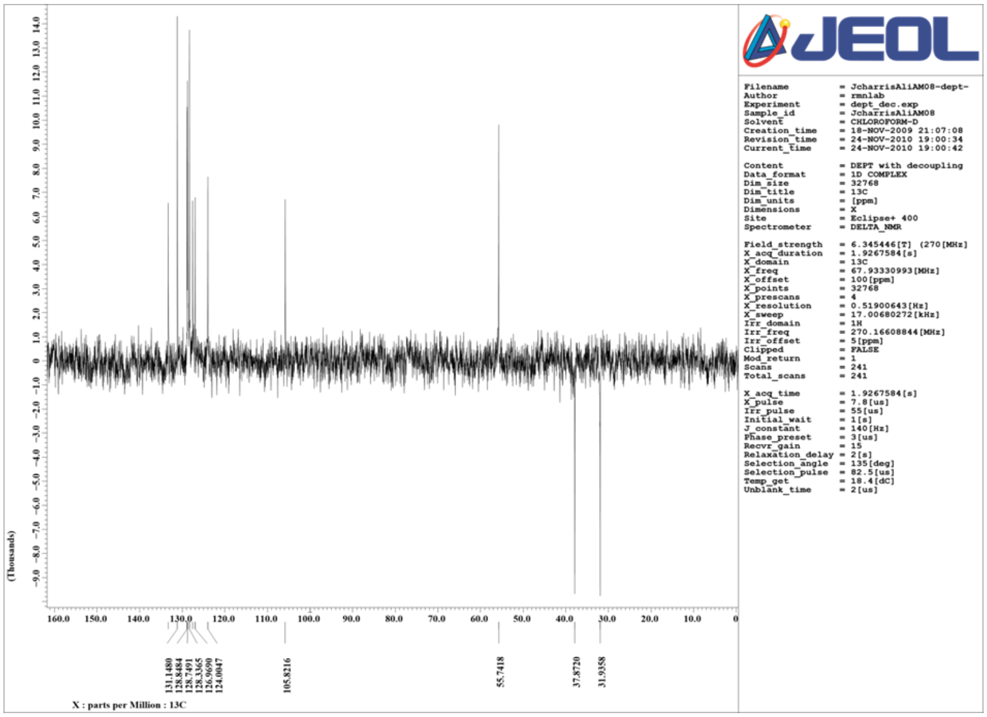

S9. Comp. 9.

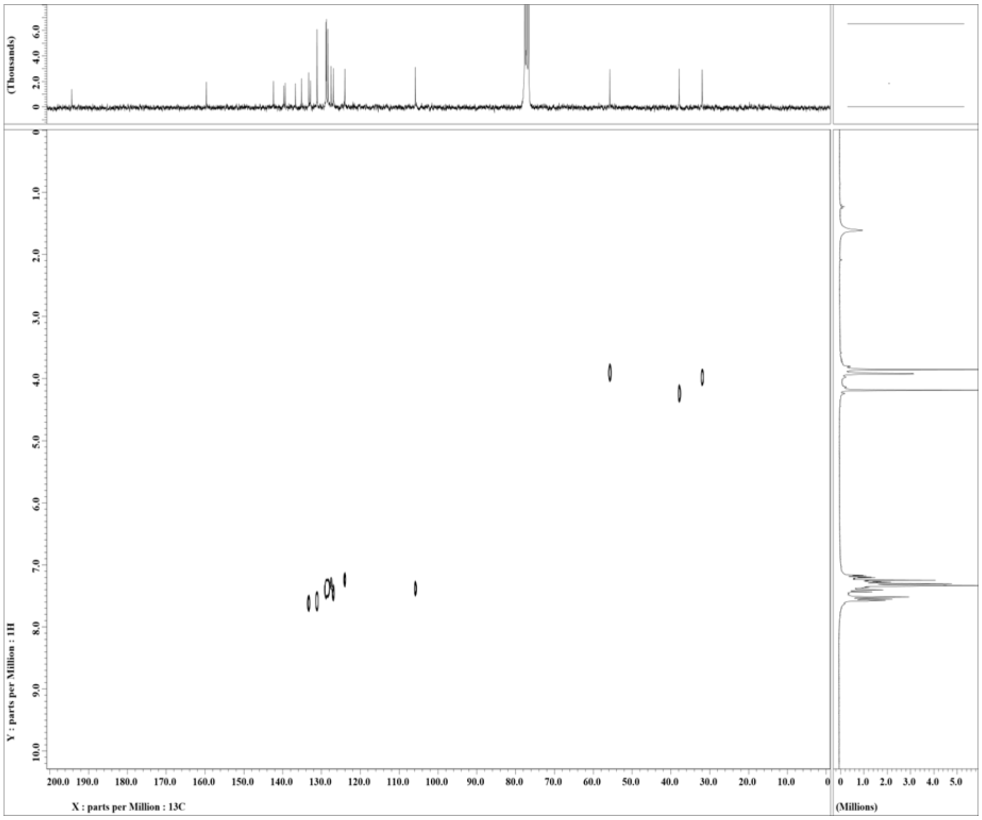

S10. Comp. 10

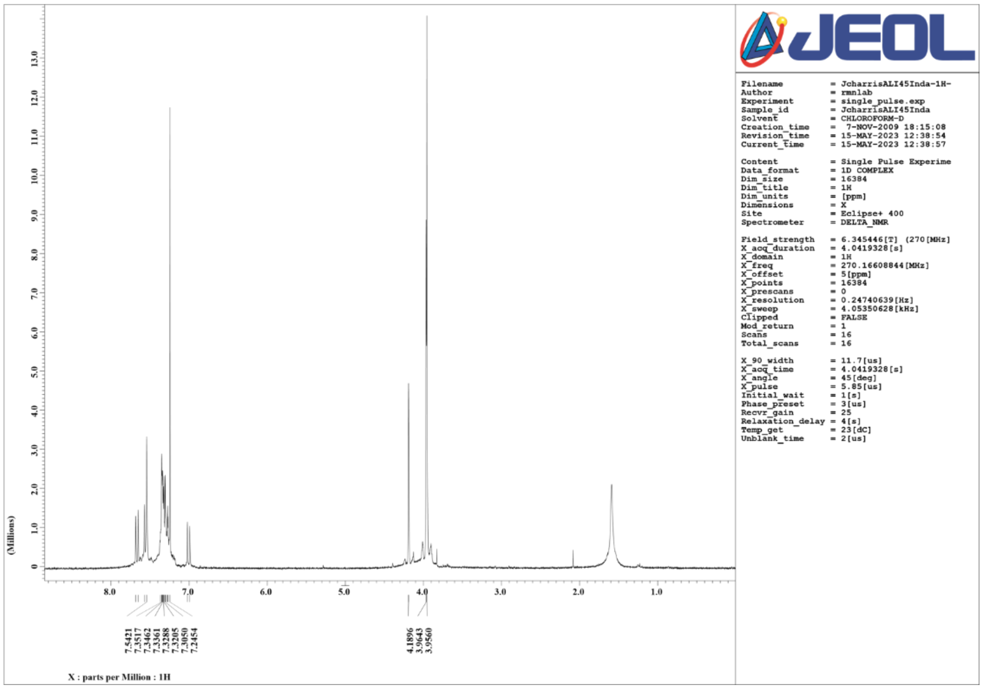

S11. Comp. 10

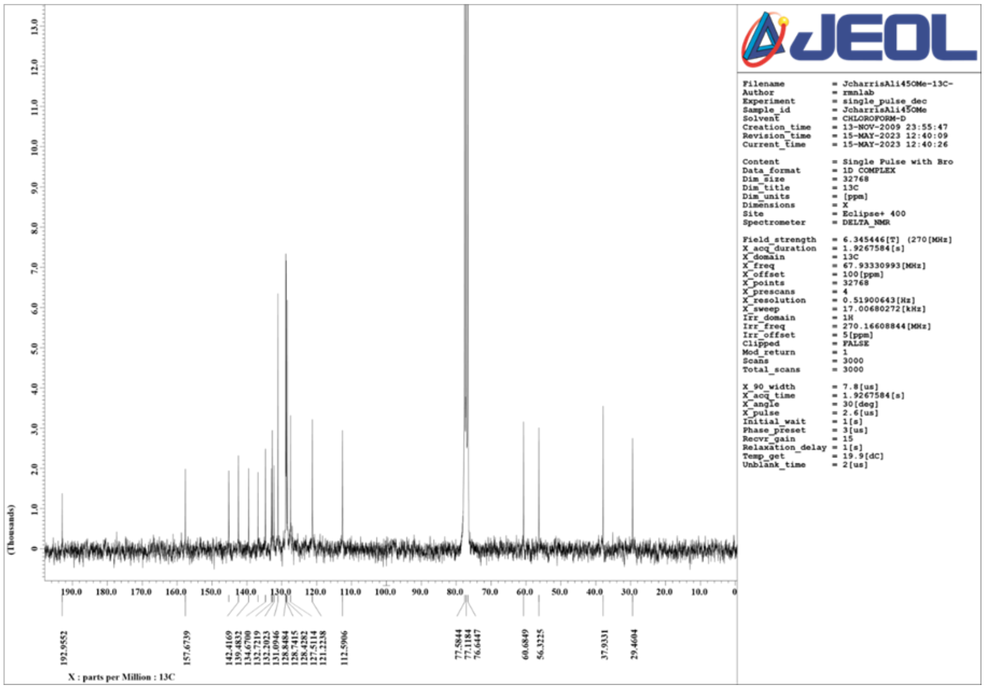

S12. Comp.10

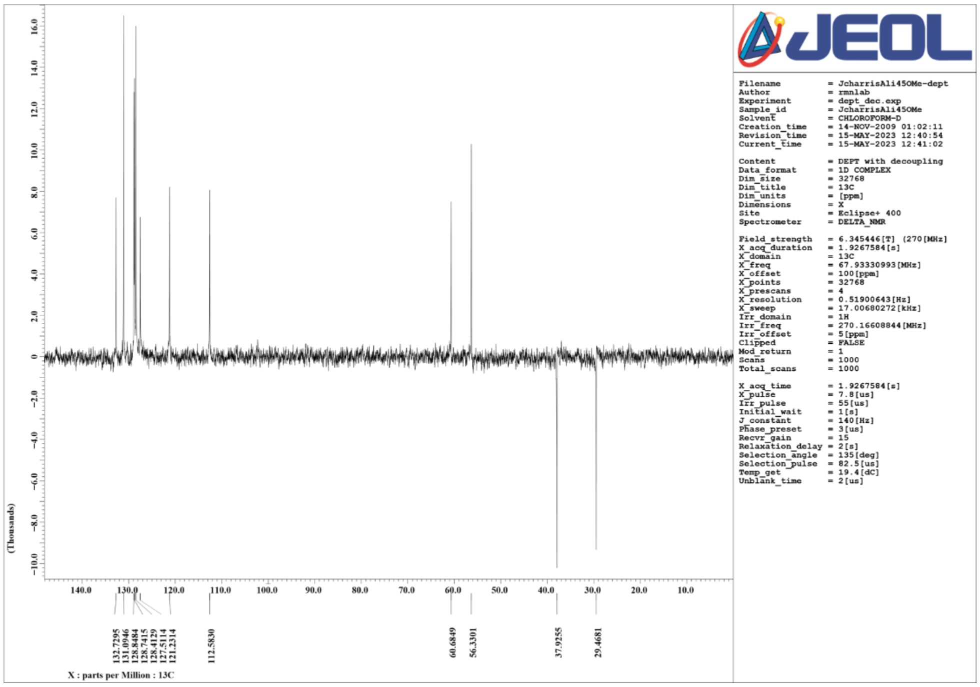

S13. Comp.10

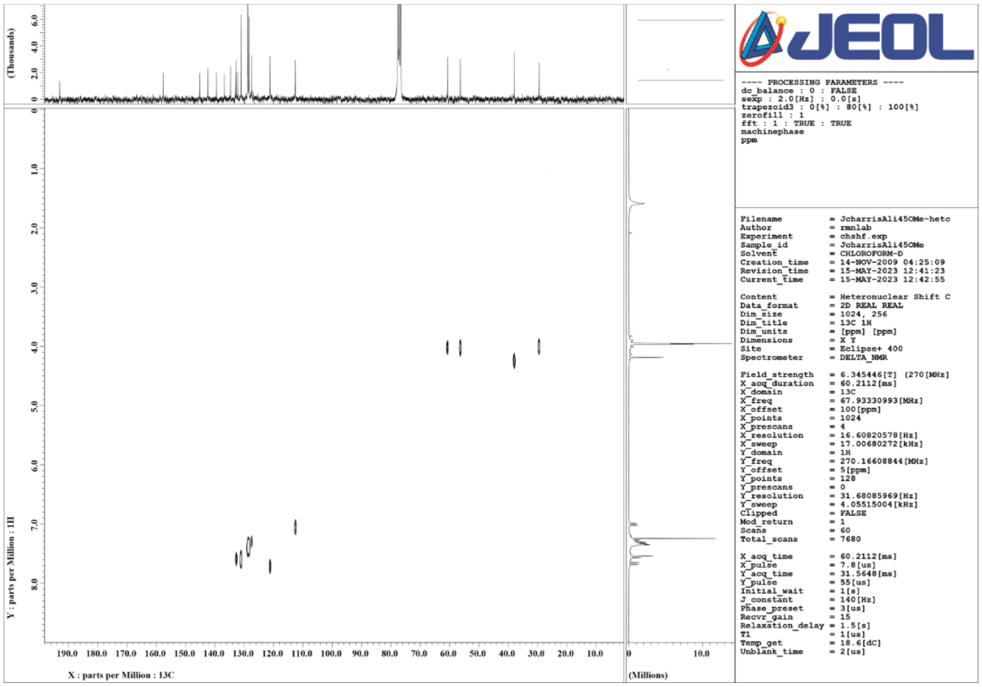

S14. Comp. 11

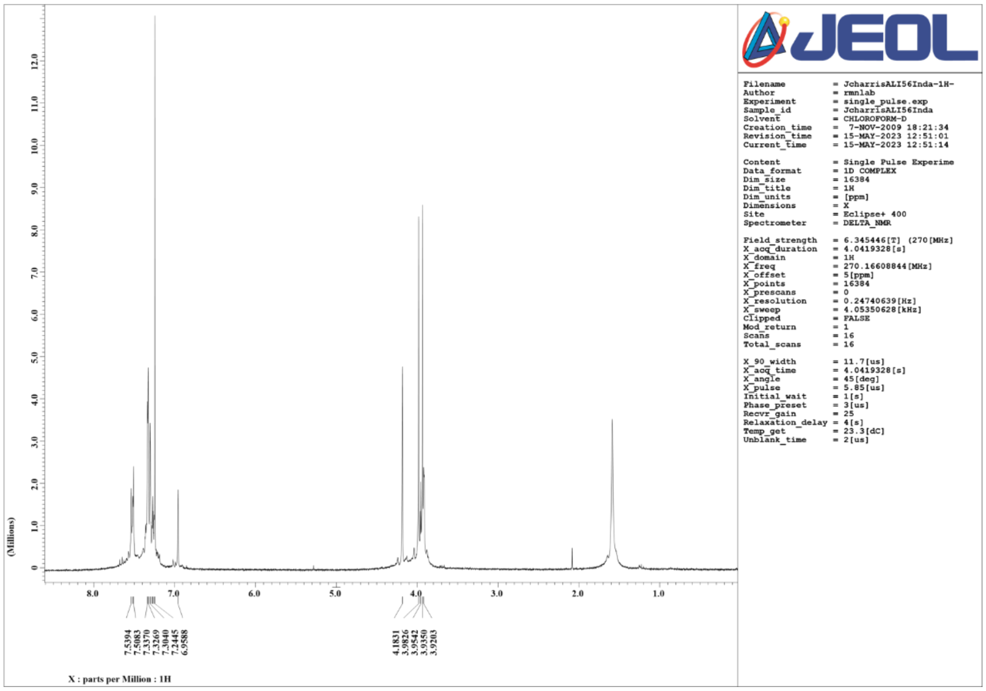

S15. Comp. 11

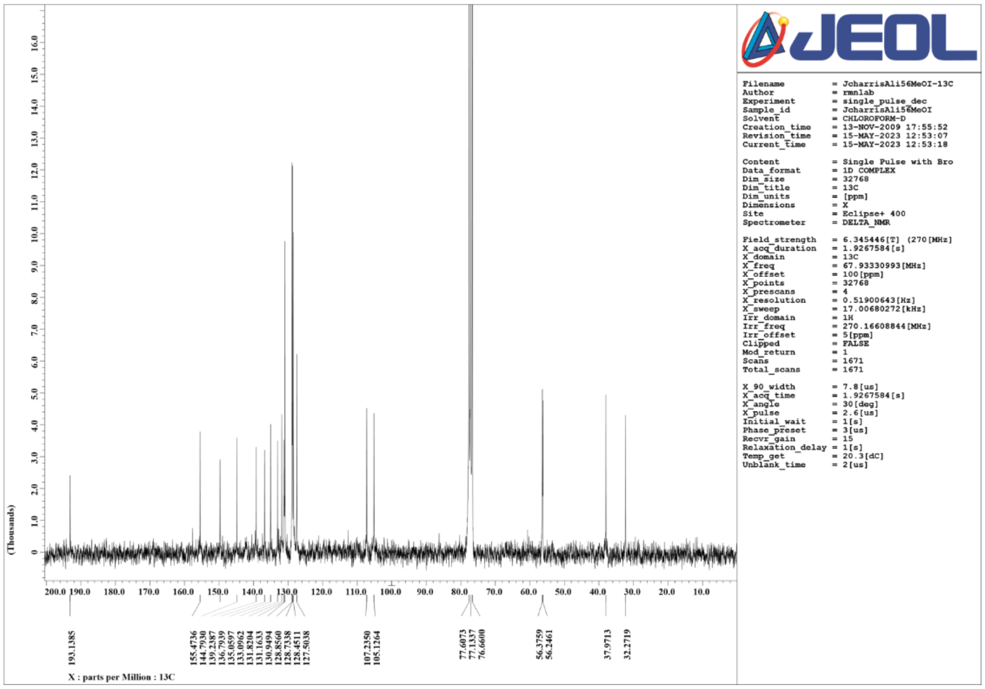

S16. Comp. 11

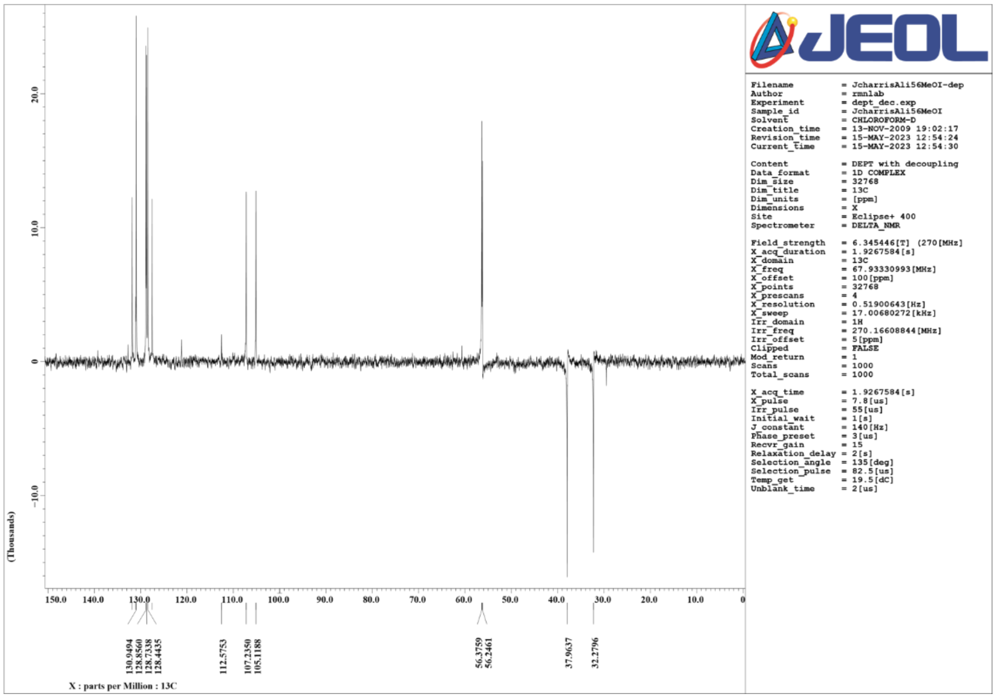

S17. Comp. 11

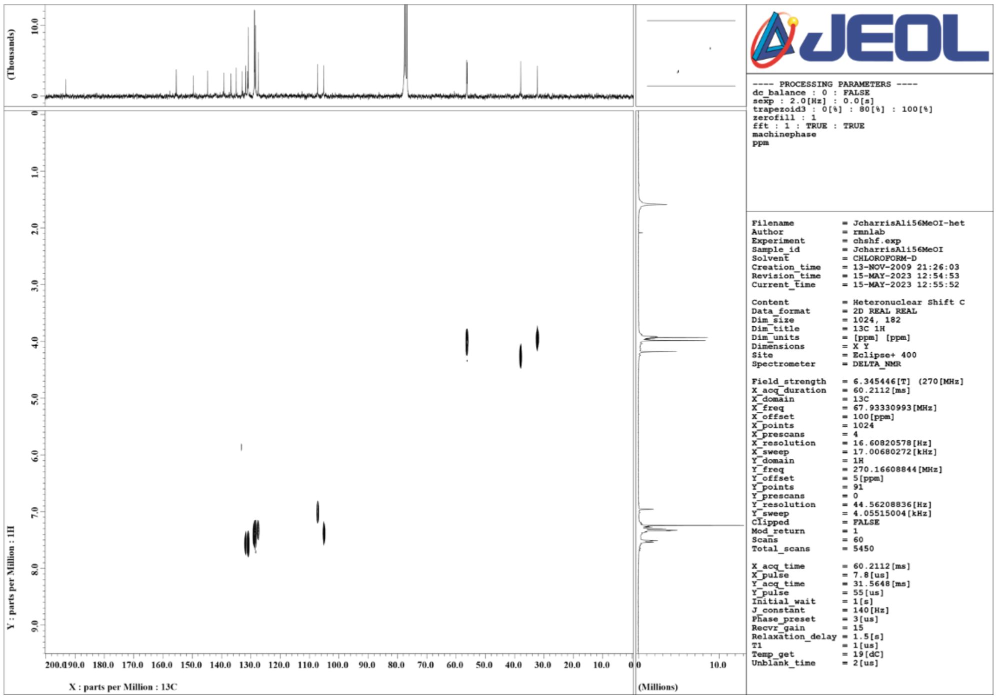

S18. Comp. 12

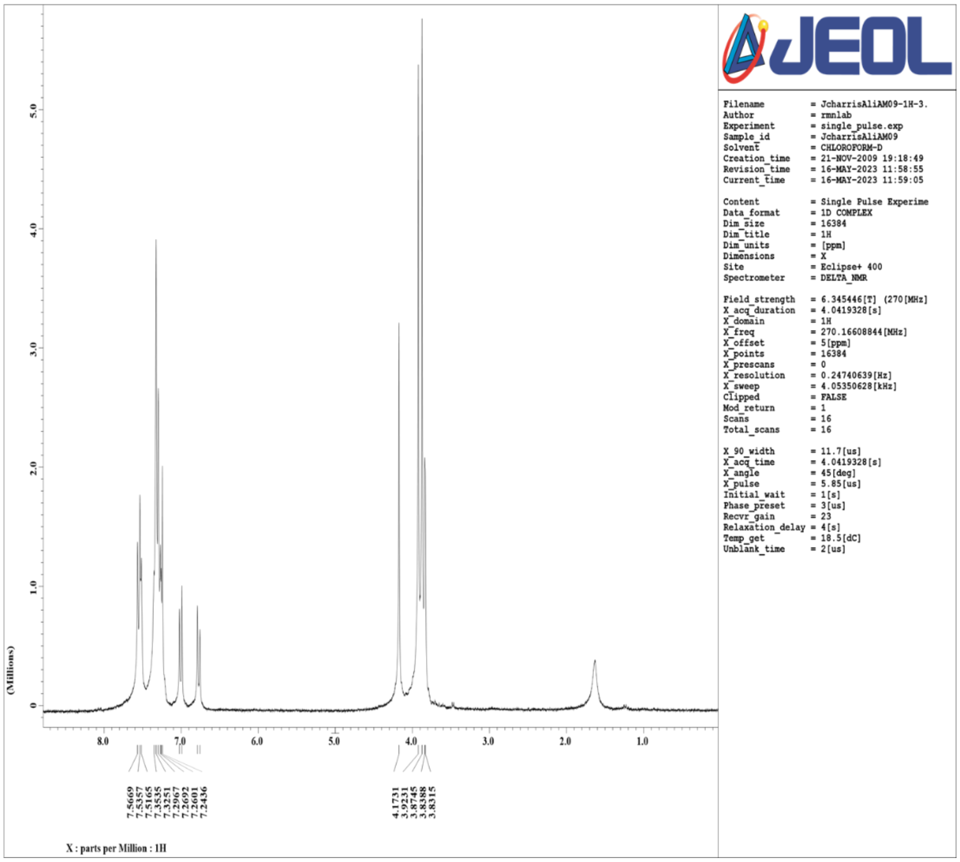

S19. Comp. 12

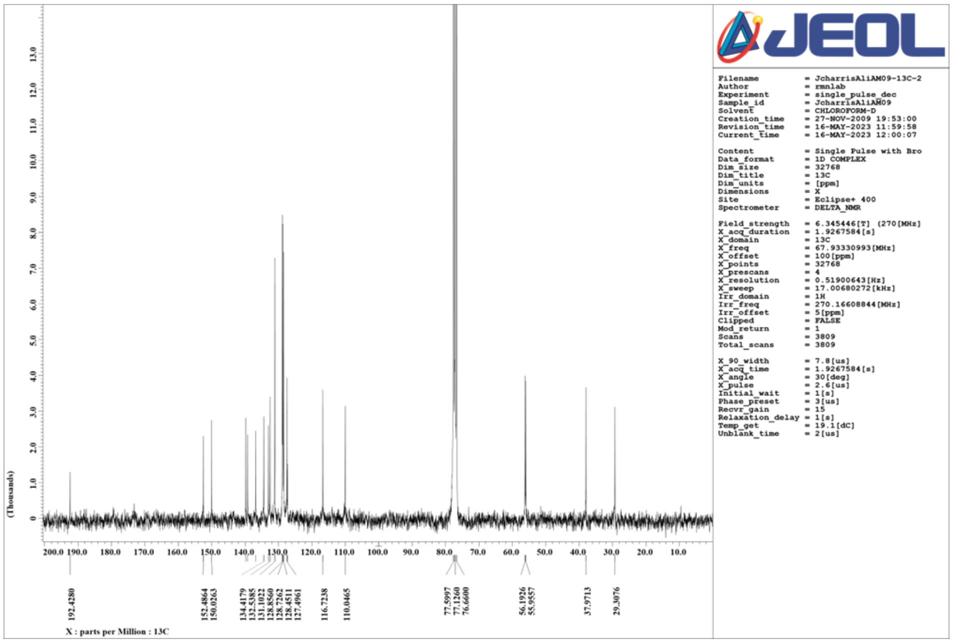

S20. Comp. 12

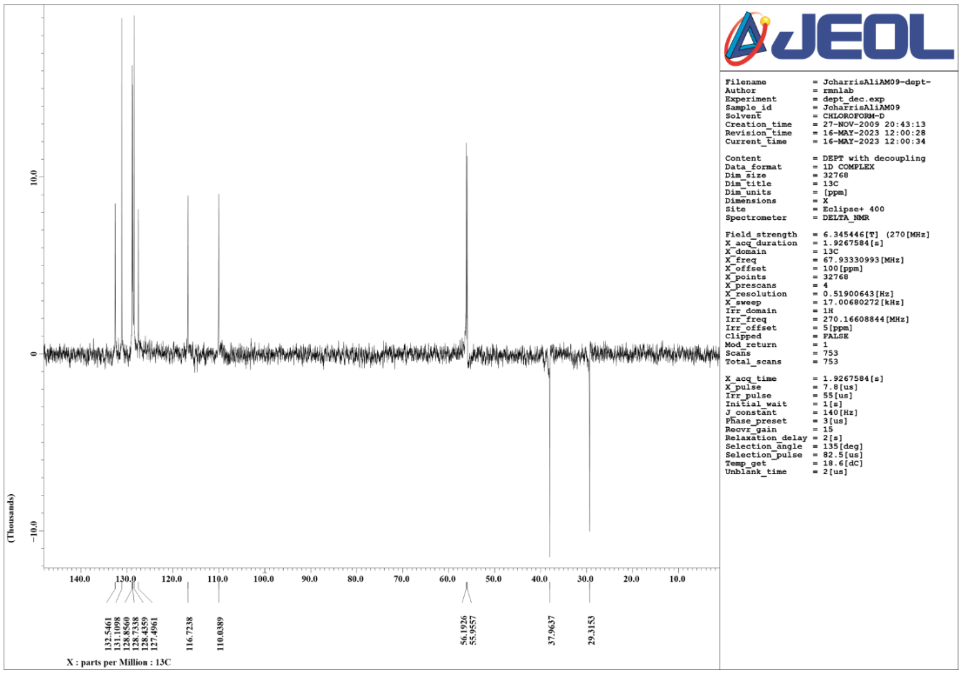

S21. Comp. 12

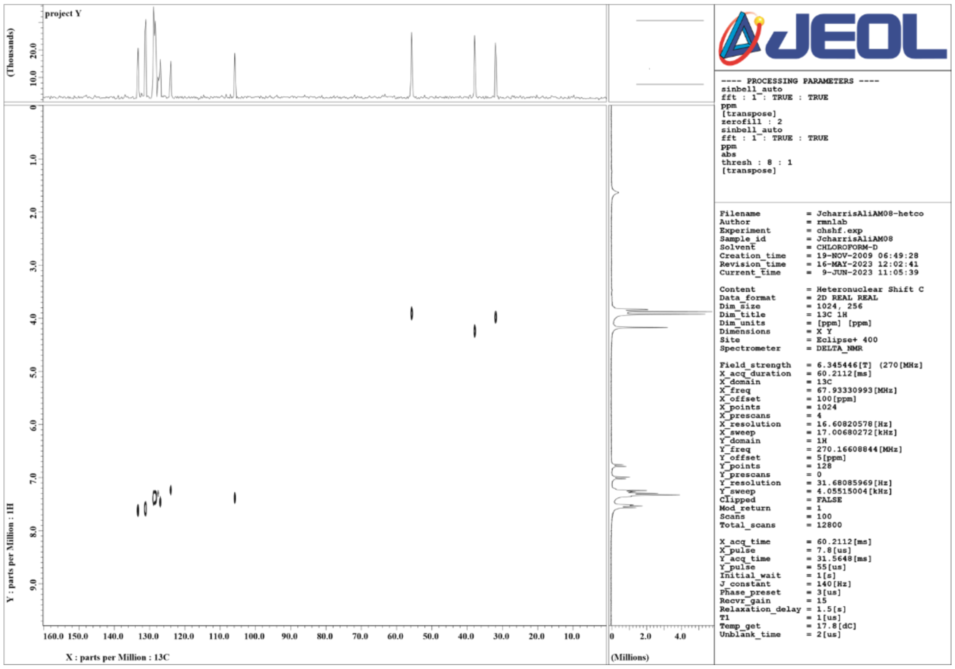

S22. Comp. 13

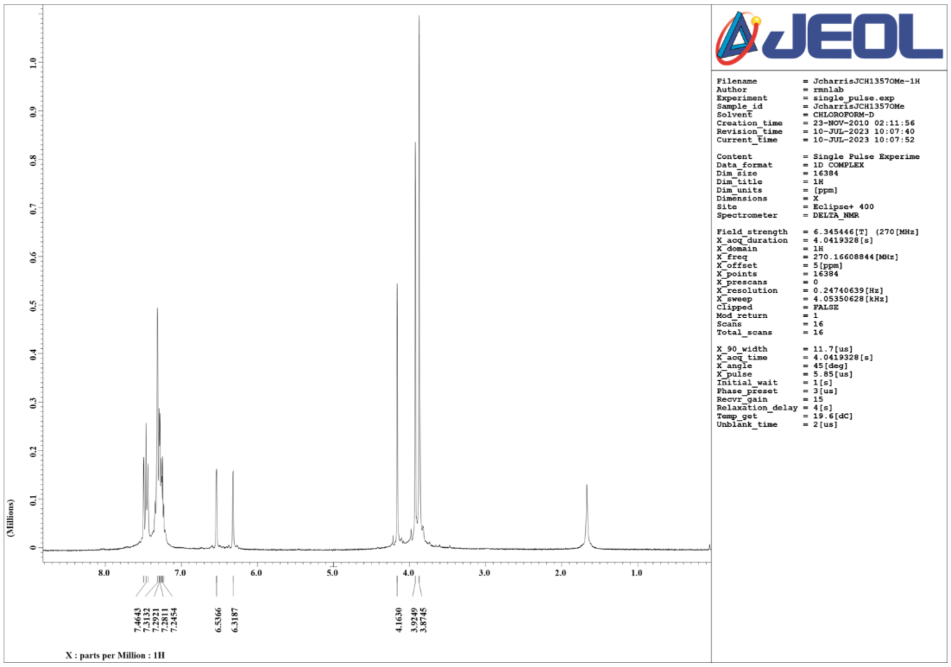

S23. Comp. 13

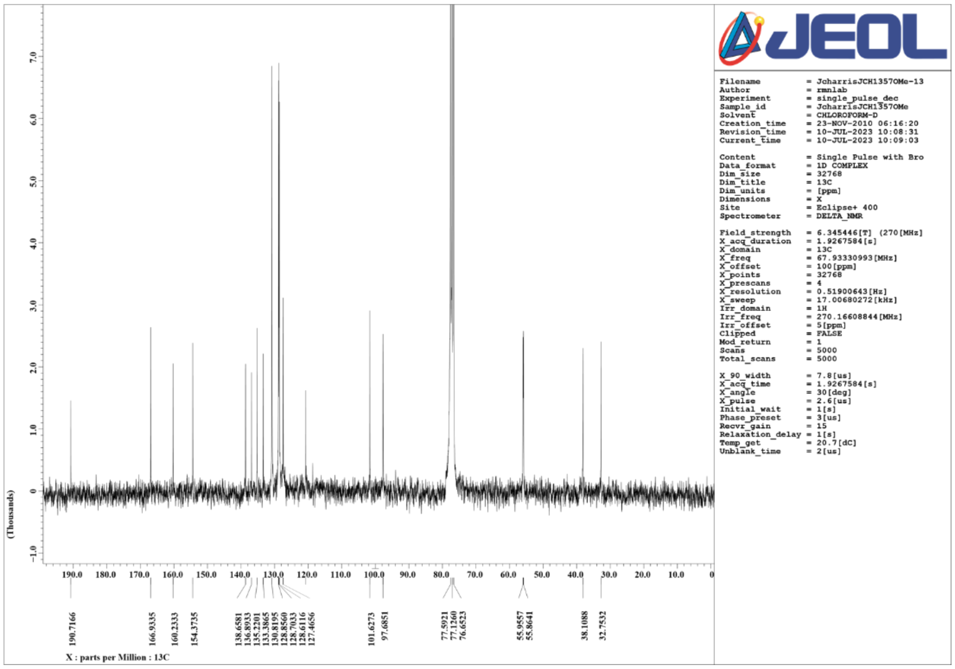

S24. Comp. 14

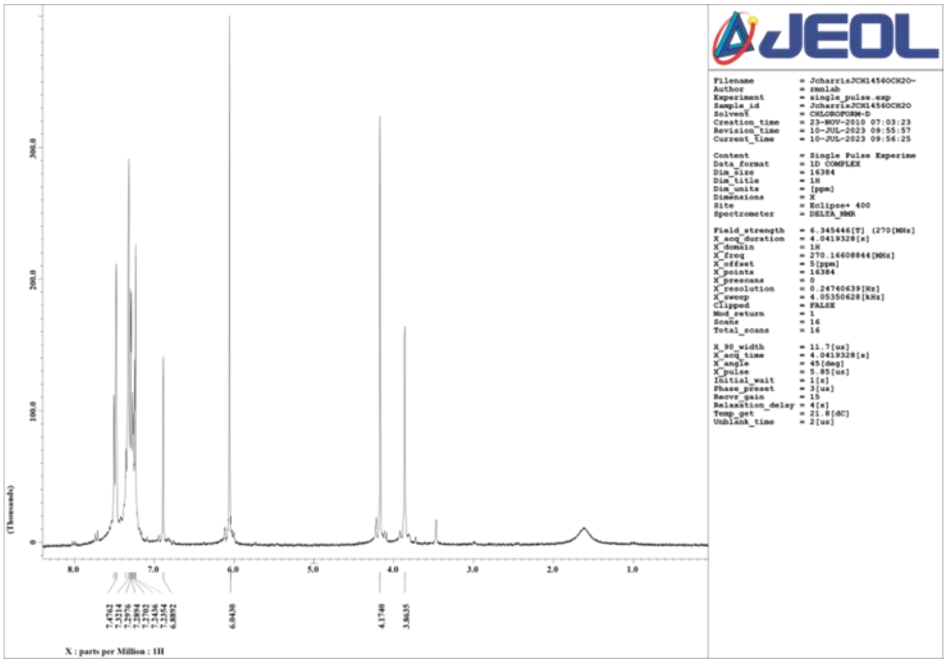

S25. Comp. 14

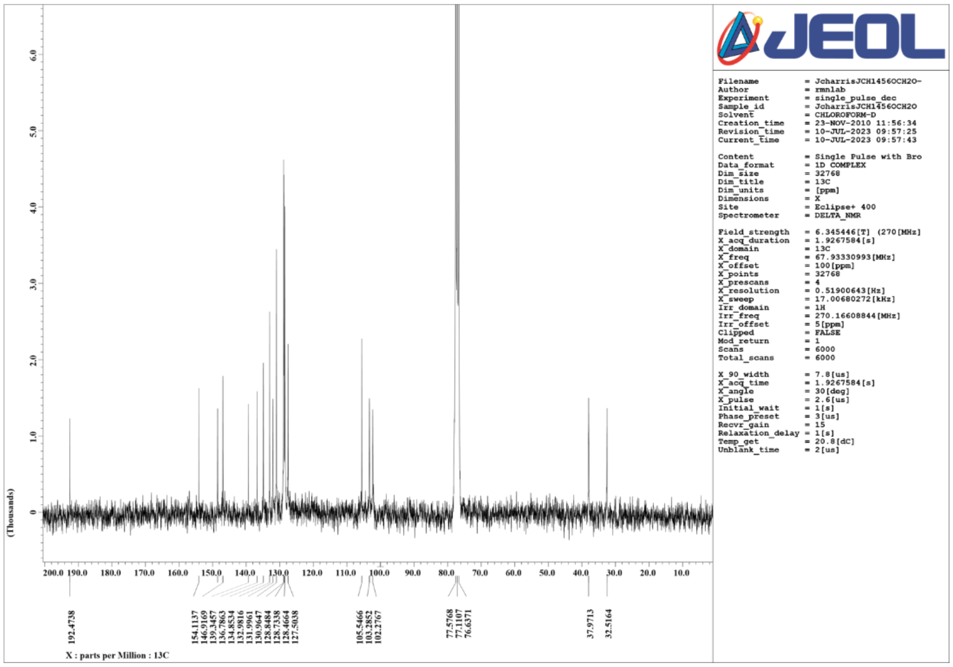

S26. Comp. 14

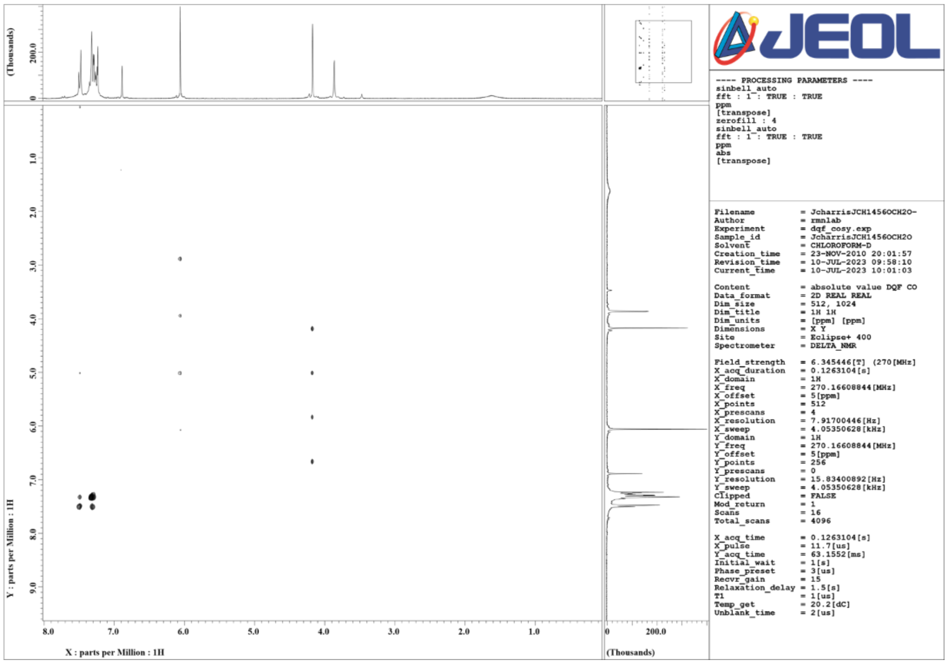

S27. Comp. 14

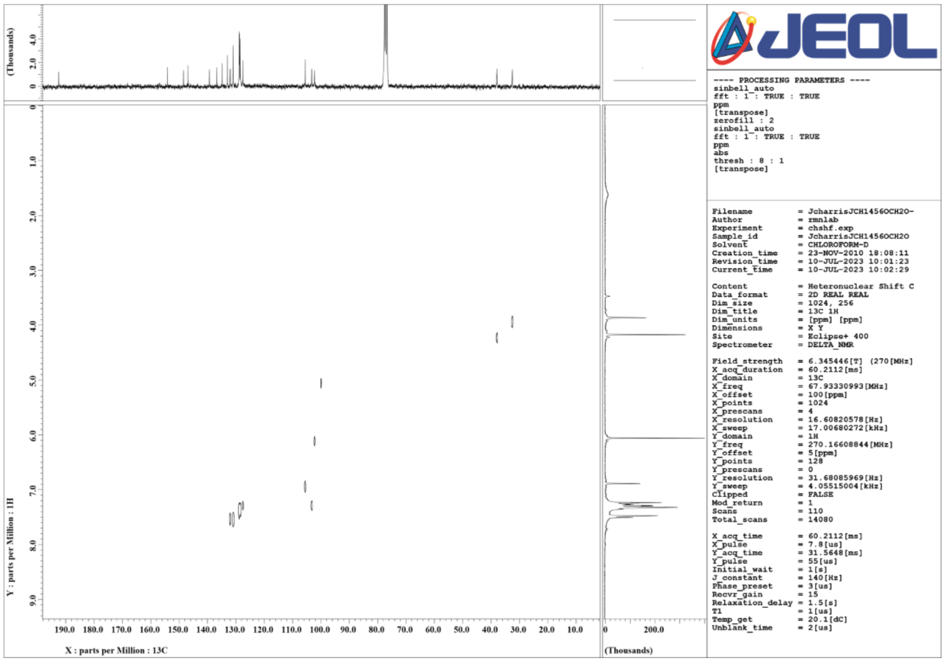

S28. Comp. 15

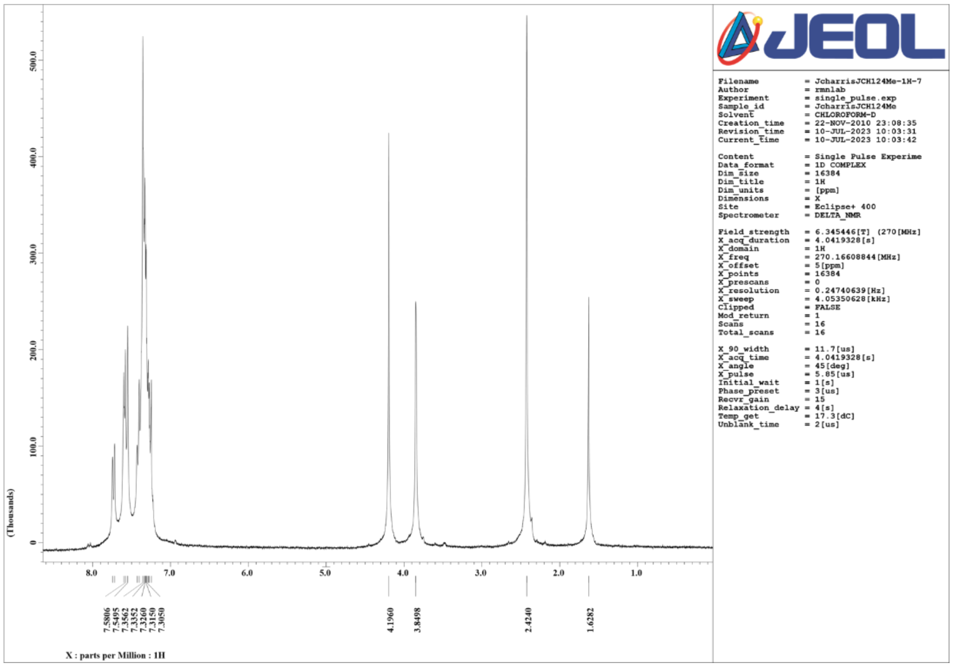

S29. Comp. 15

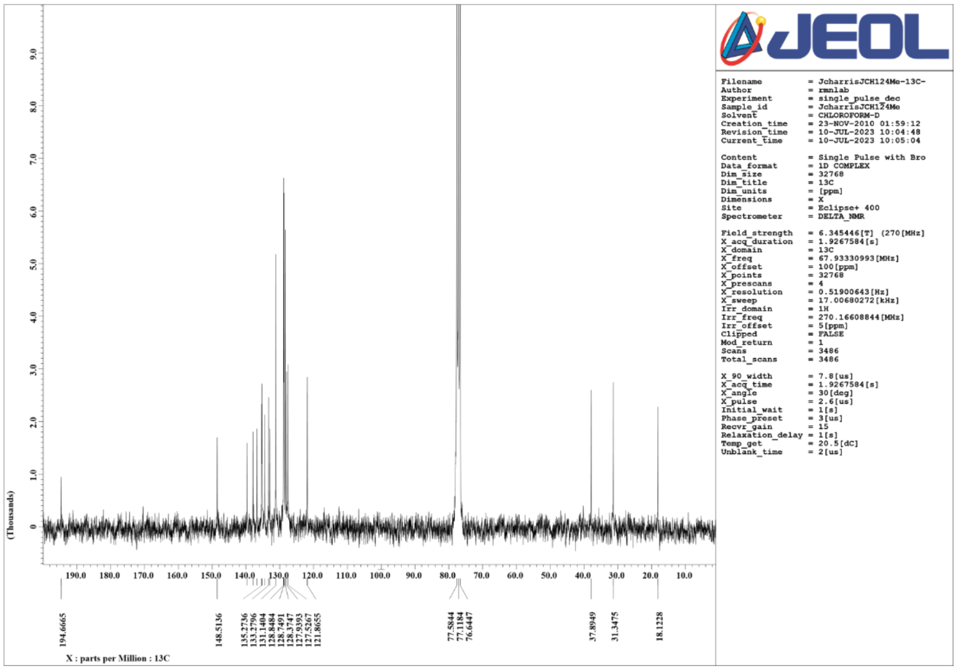

S30. Comp.16

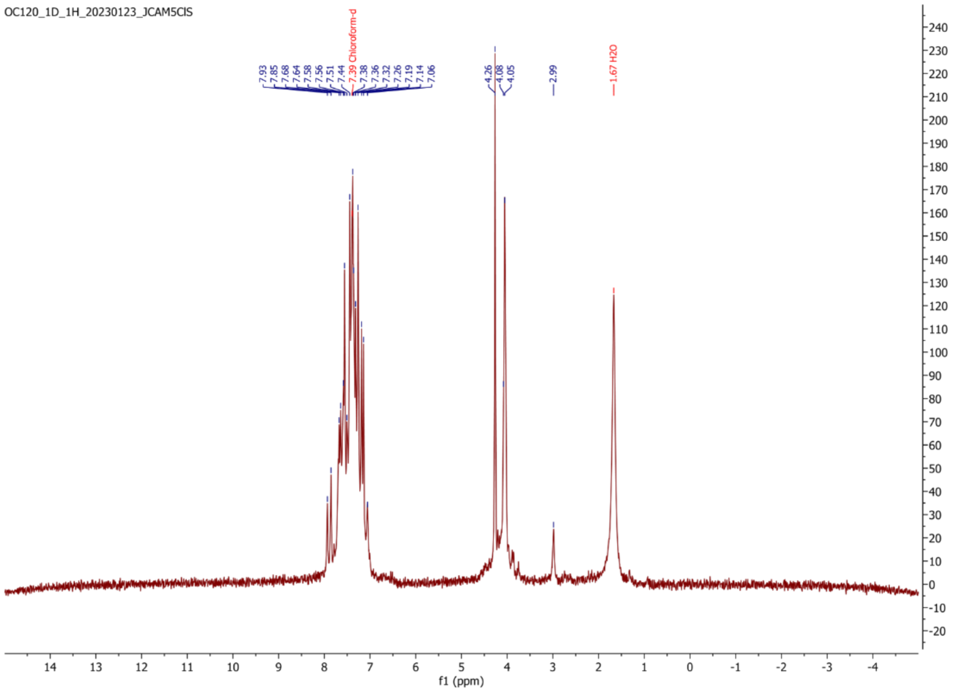

S31. Comp. 16

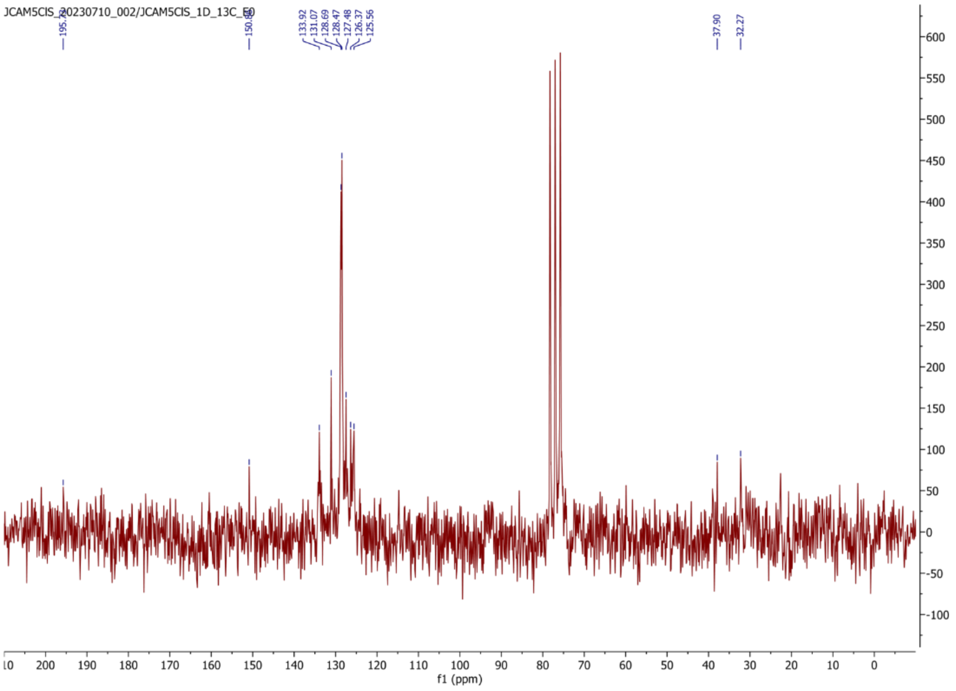

S32. Comp. 17

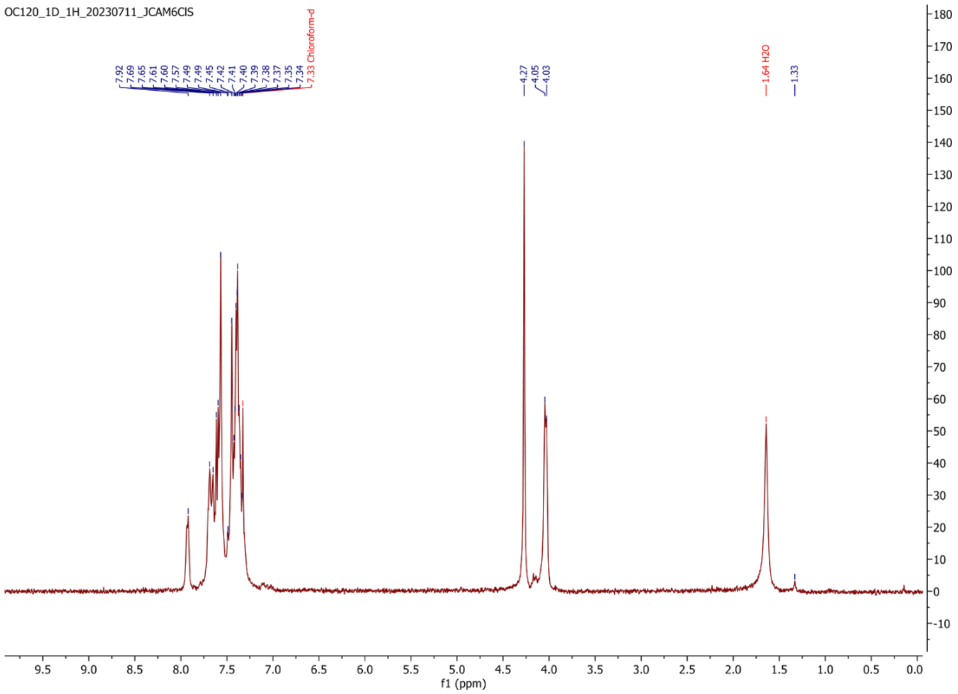

S33. Comp. 17

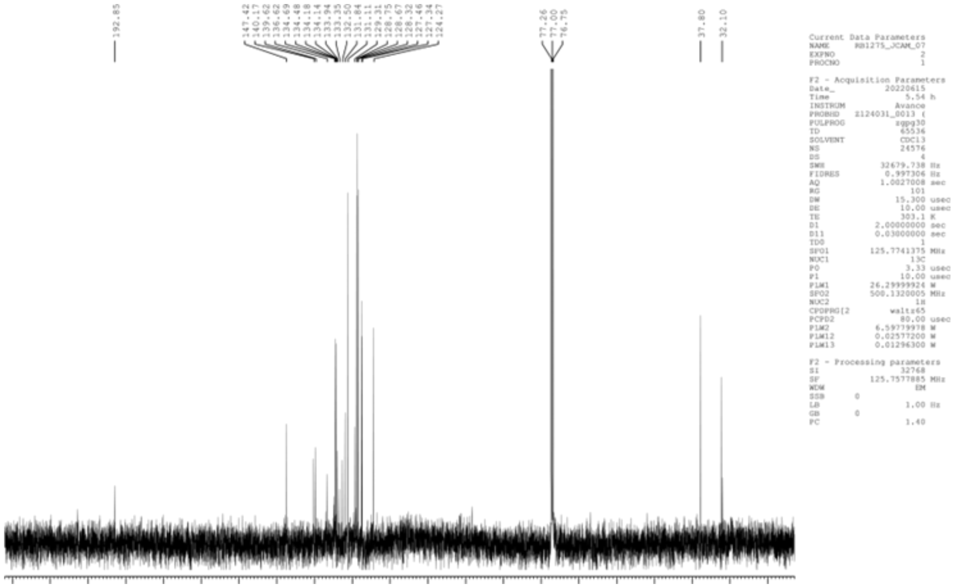

S34 Comp. 18

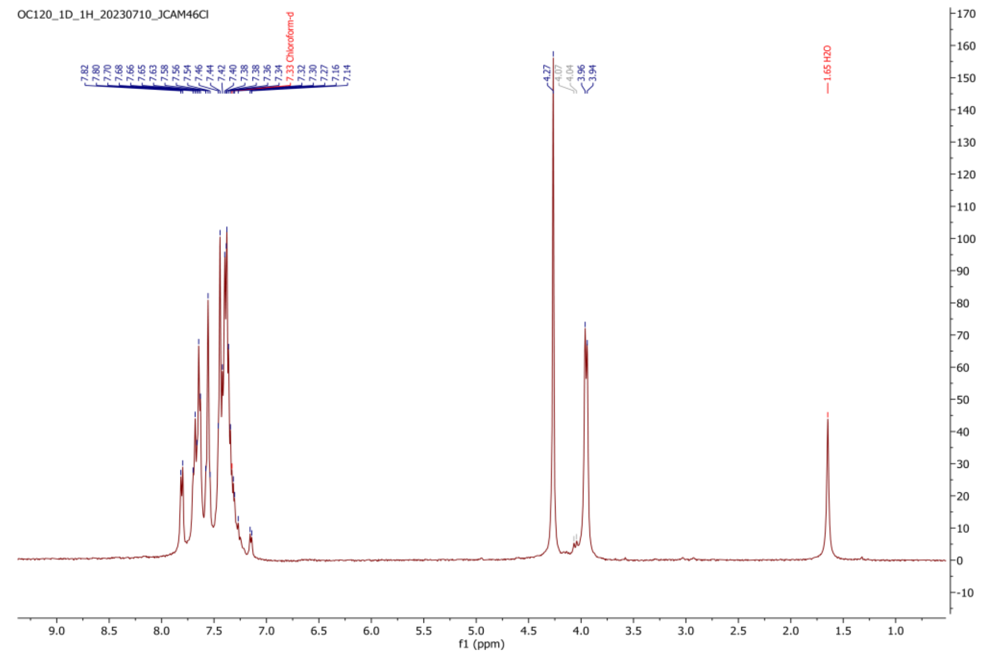

S35. Comp. 18

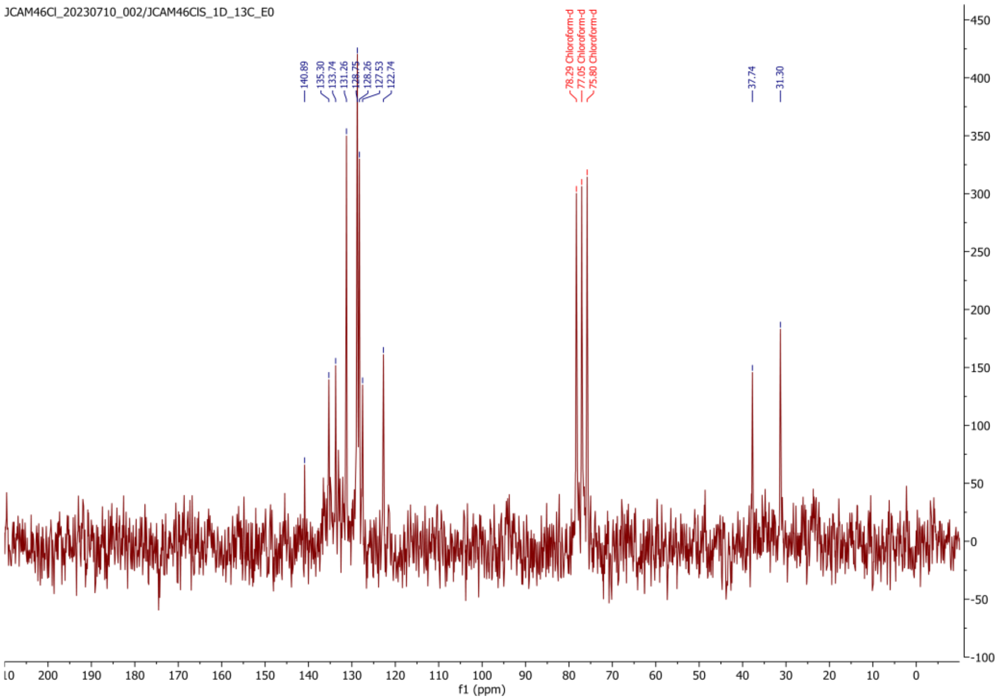

S36. Comp. 18

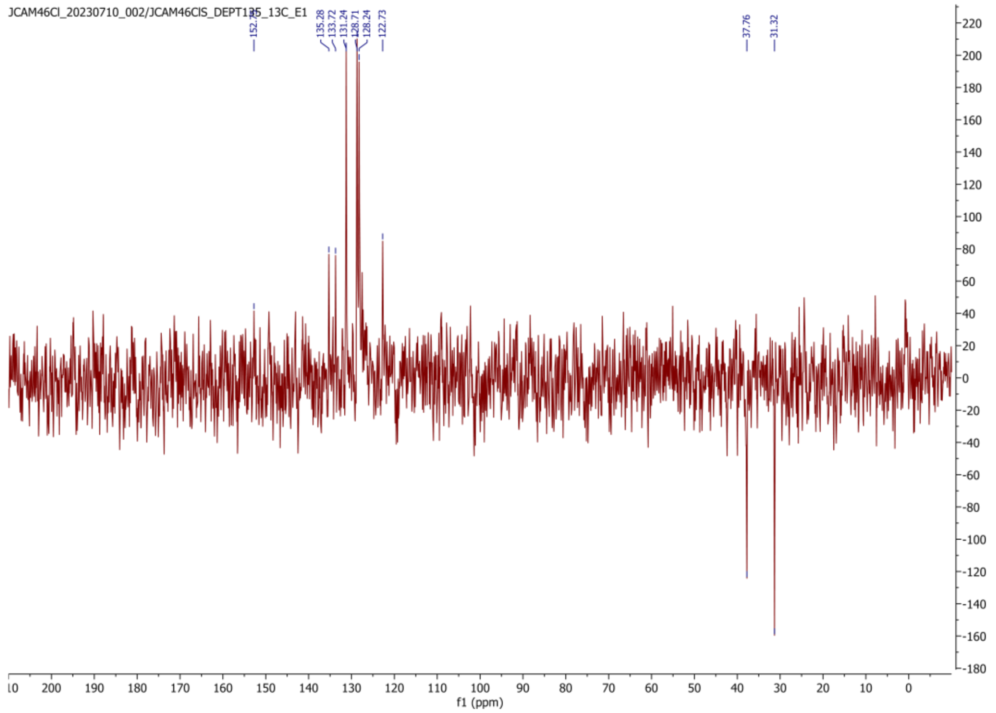
JCAM46Cl_20230710_002/JCAM46ClS_DEPT135_13C_E1
152.7
135.28
133.72
131.24
128.71
128.24
122.73
37.76
31.32
f1 (ppm)

S37. Comp. 19

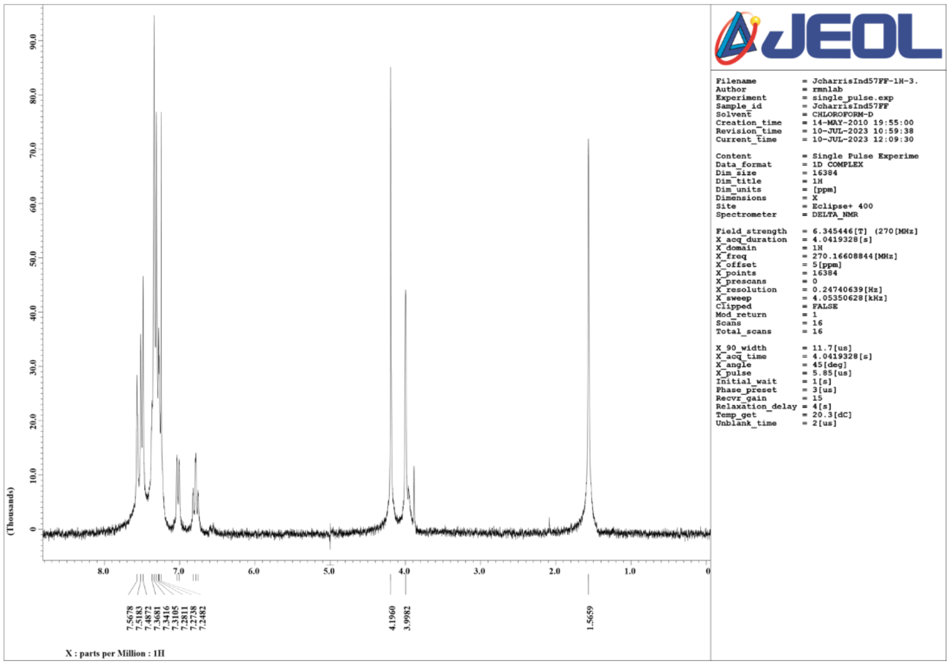

S38. Comp. 19

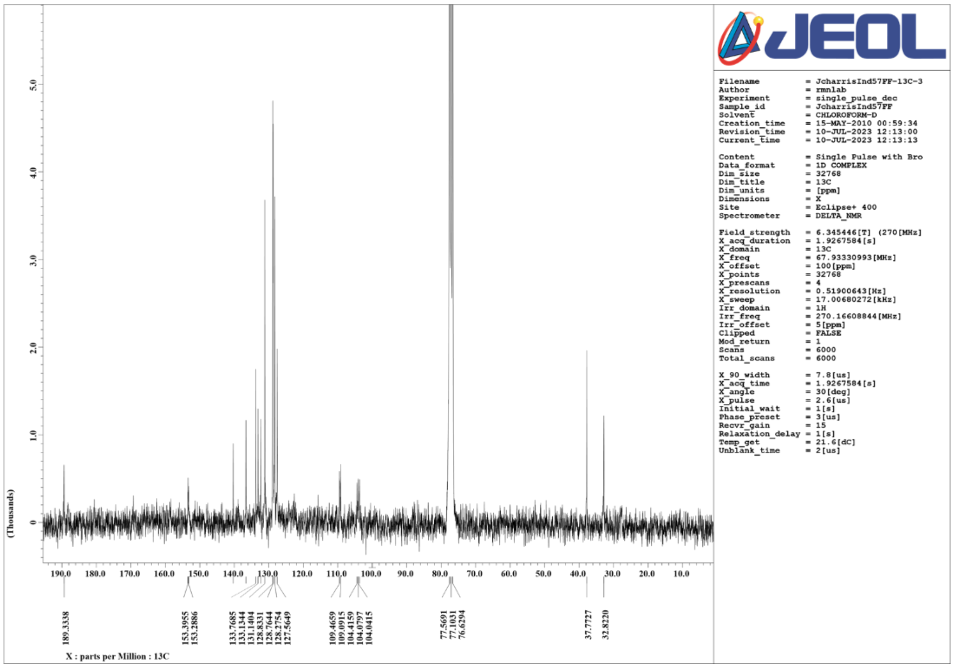

S39. Comp. 19

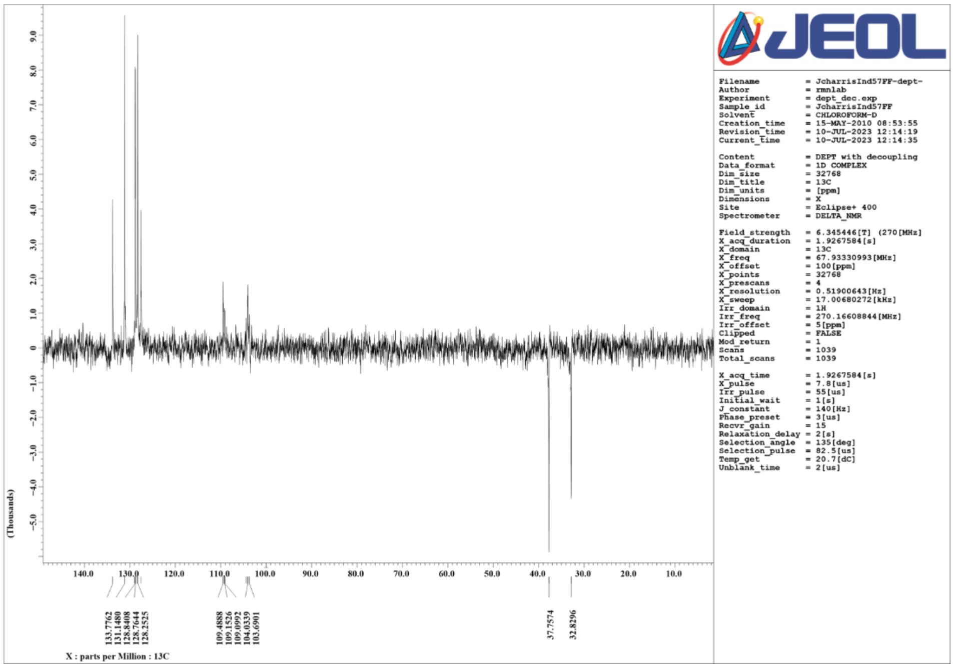

S40. Comp 19

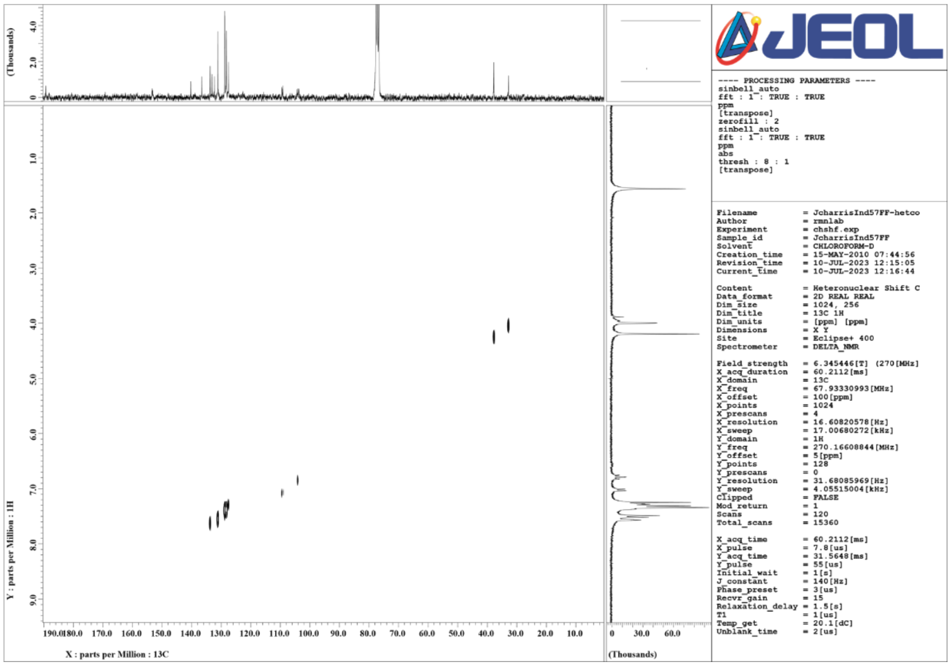

S41. Comp. 20

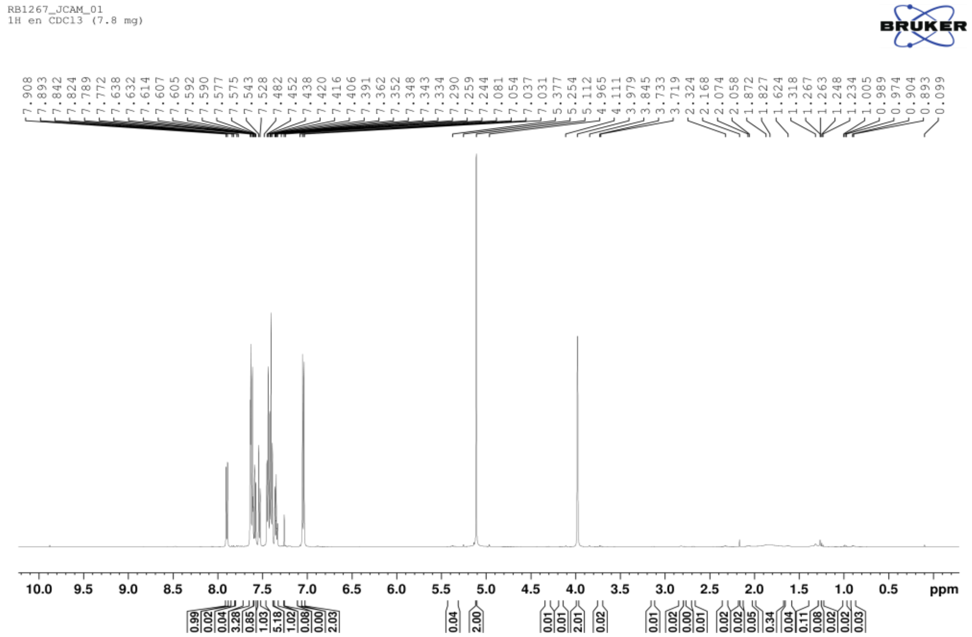

S42. Comp. 20

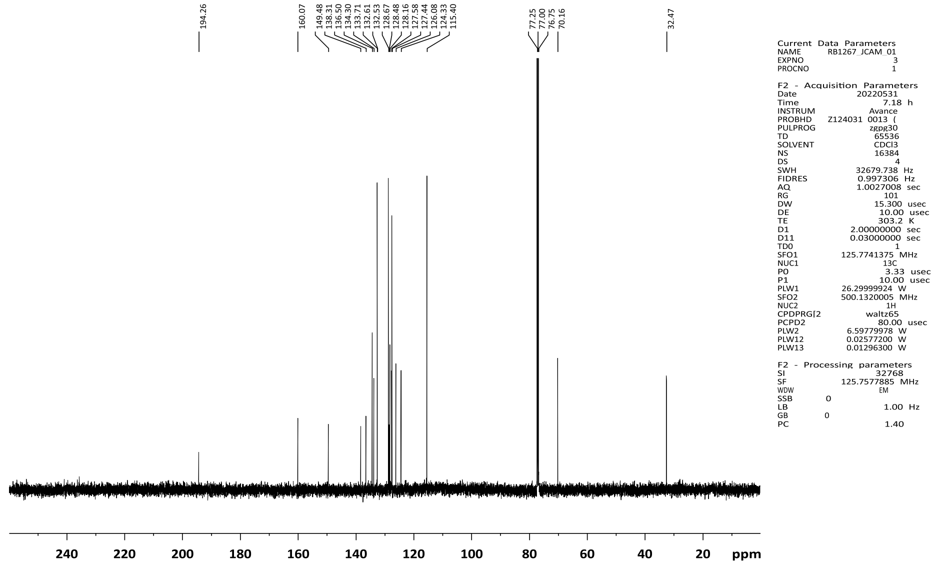

S43. Comp. 20

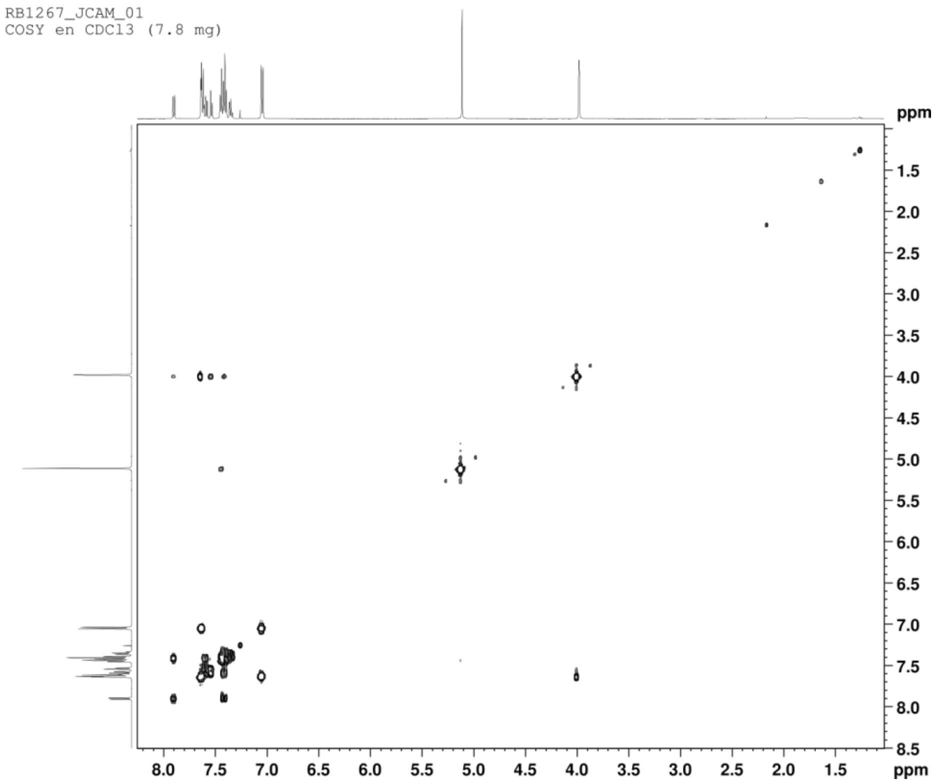

S44. Comp. 20

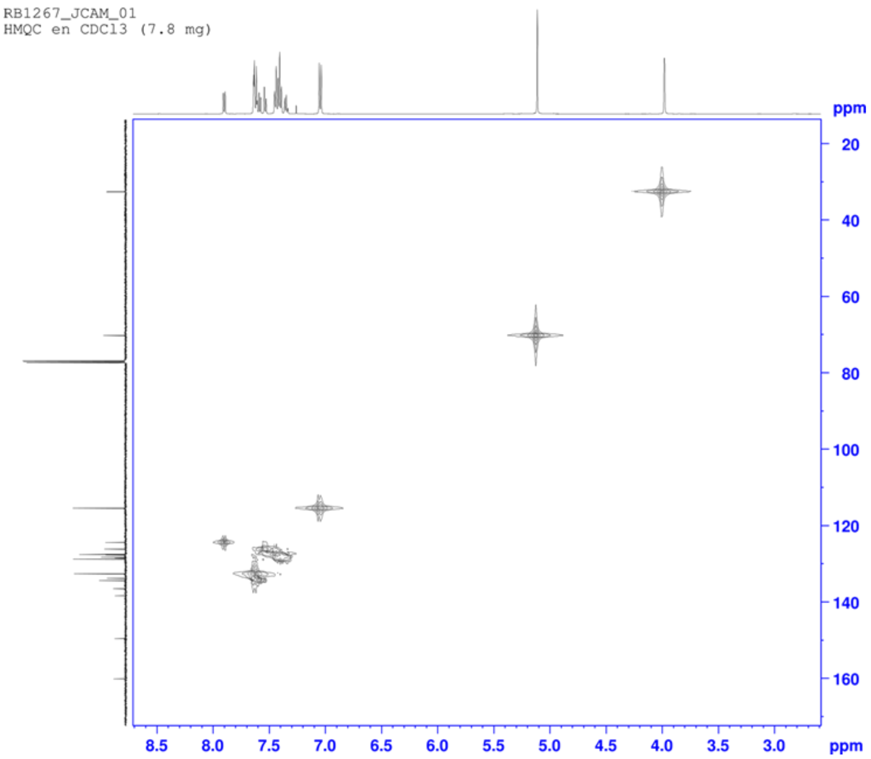

S45. Comp. 20

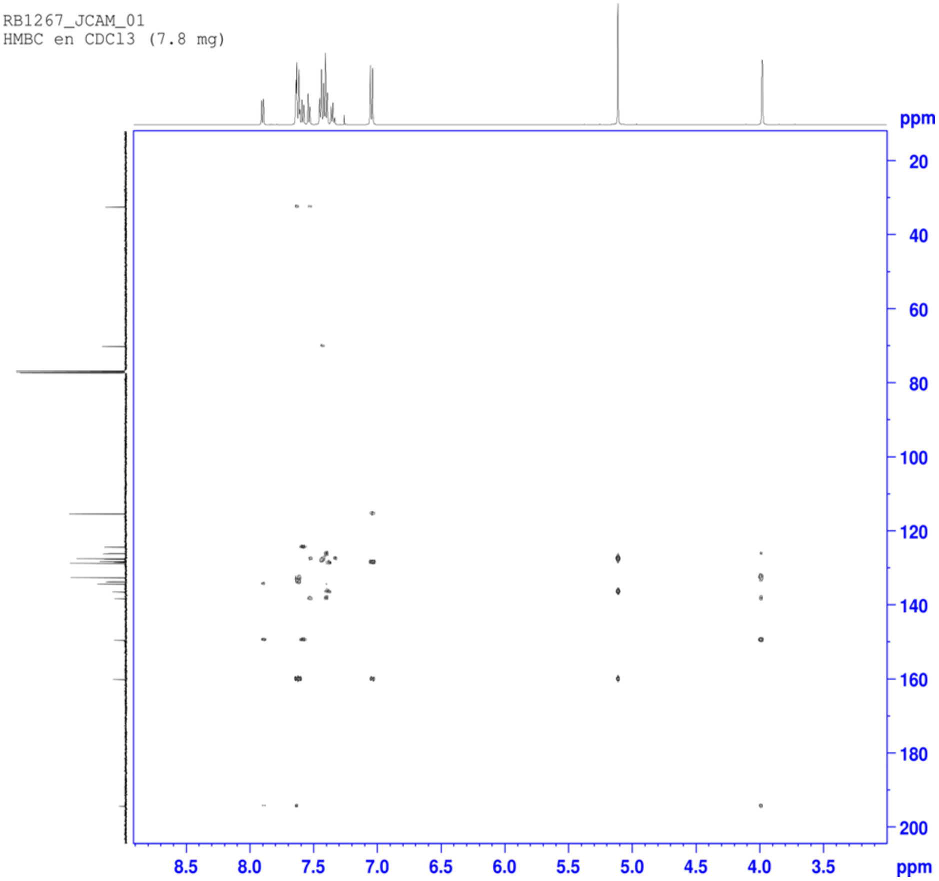
RB1267_JCAM_01
HMBC en CDCl3 (7.8 mg)
ppm
20
40
60
80
100
120
140
160
180
200
8.5
8.0
7.5
7.0
6.5
6.0
5.5
5.0
4.5
4.0
3.5
ppm

S46. Comp. 22

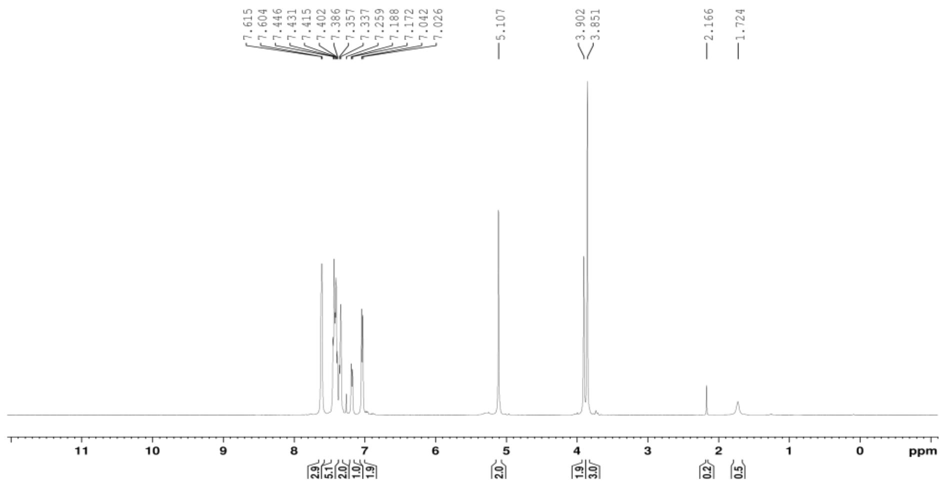

S47. Comp. 22

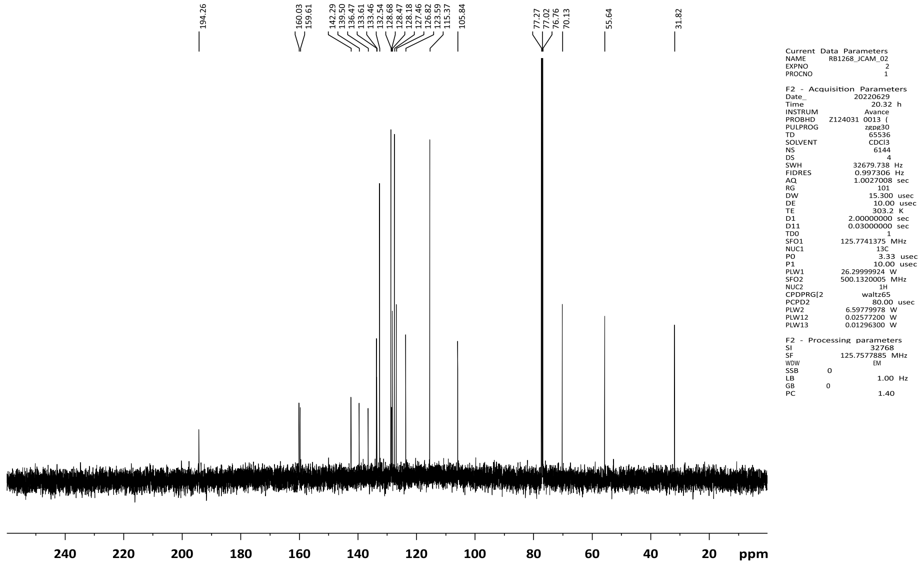

S48. Comp. 22

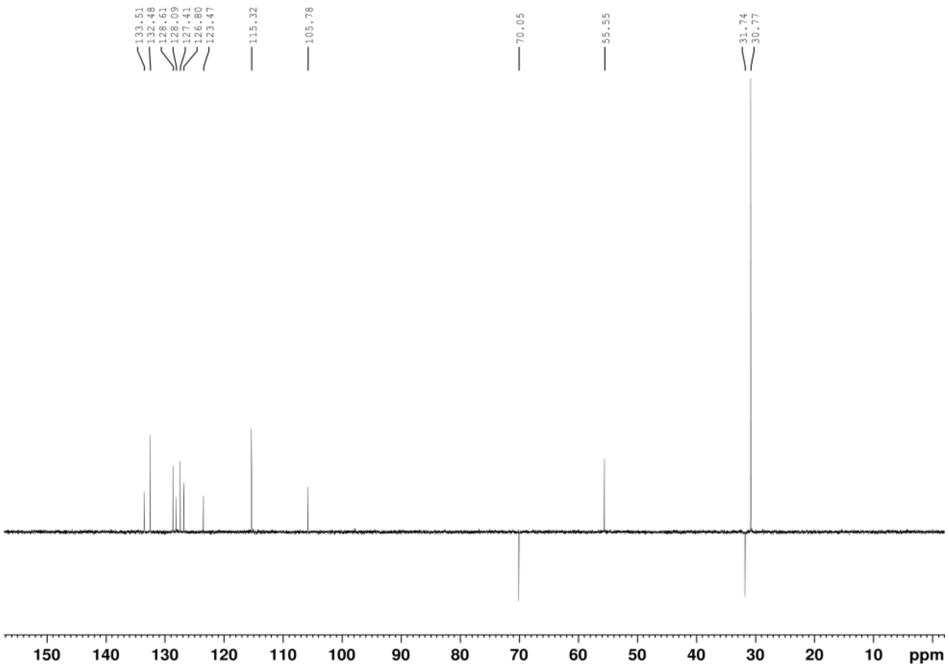

S49. Comp. 22

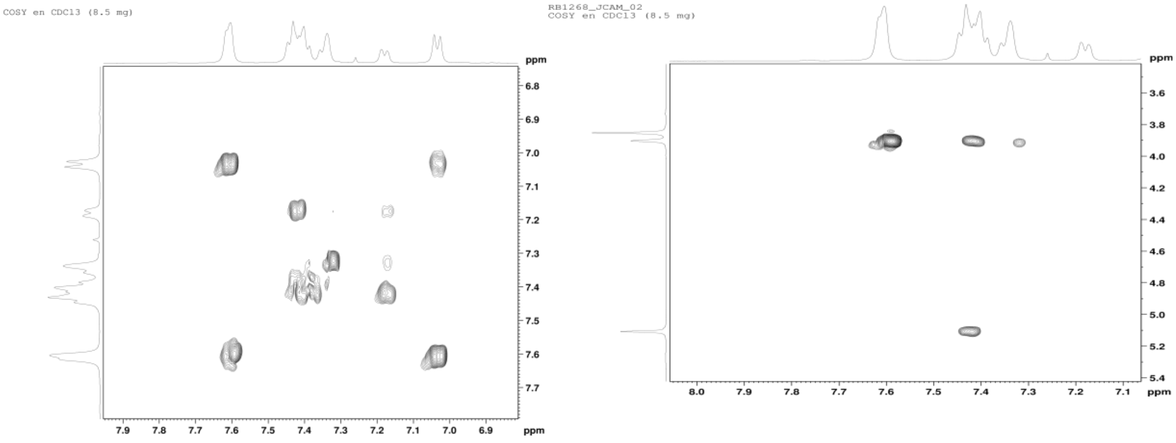

S50. Comp. 22

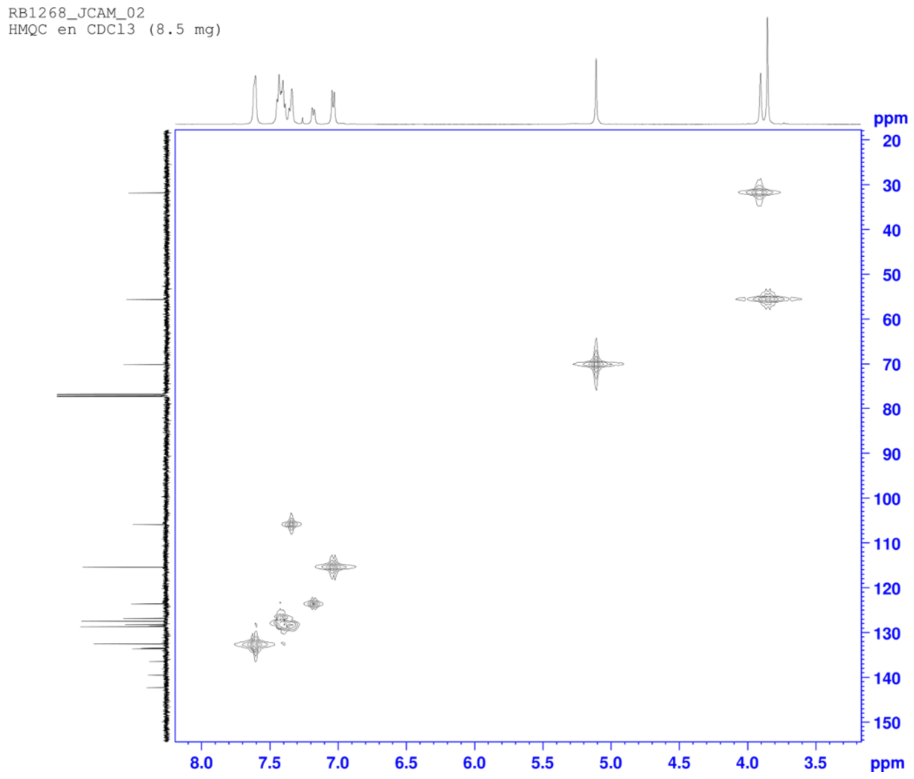

S51. Comp. 22

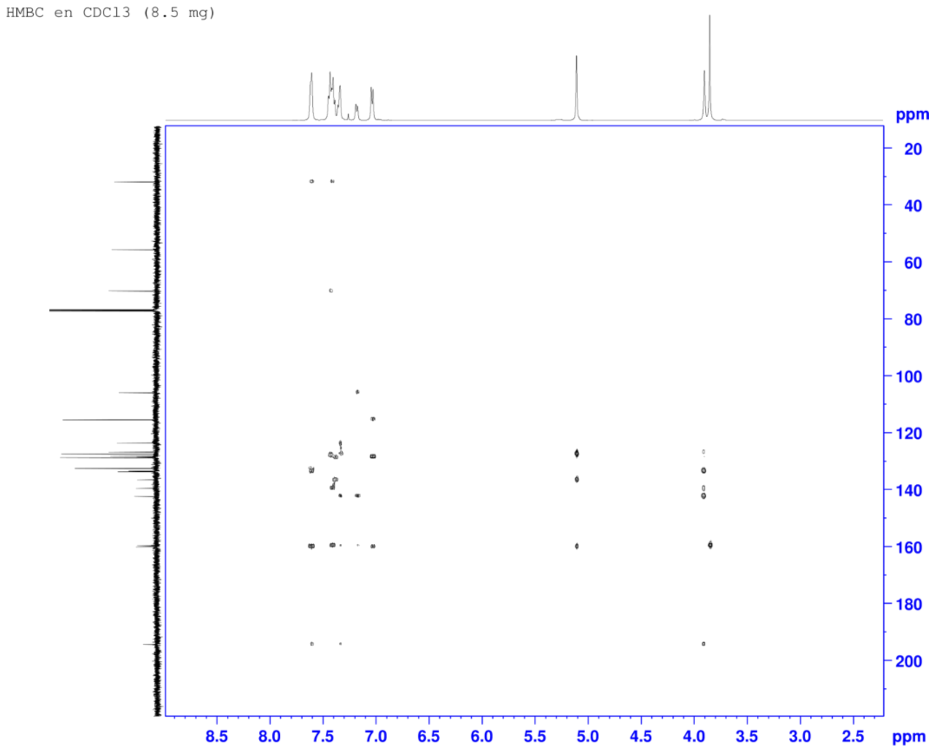

S52. Comp. 23

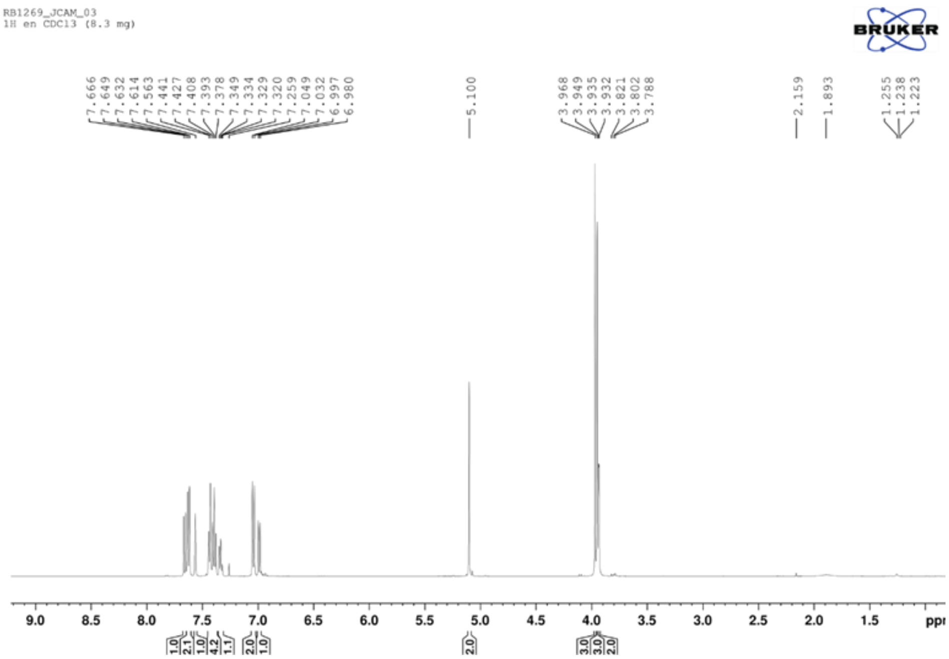

S53. Comp. 23

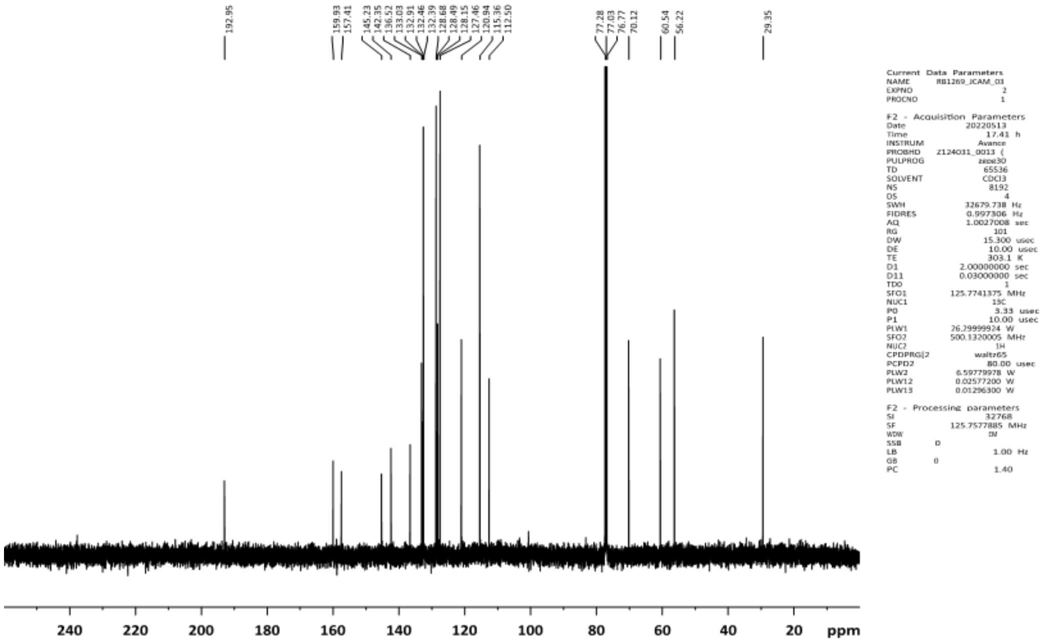

S54. Comp. 23

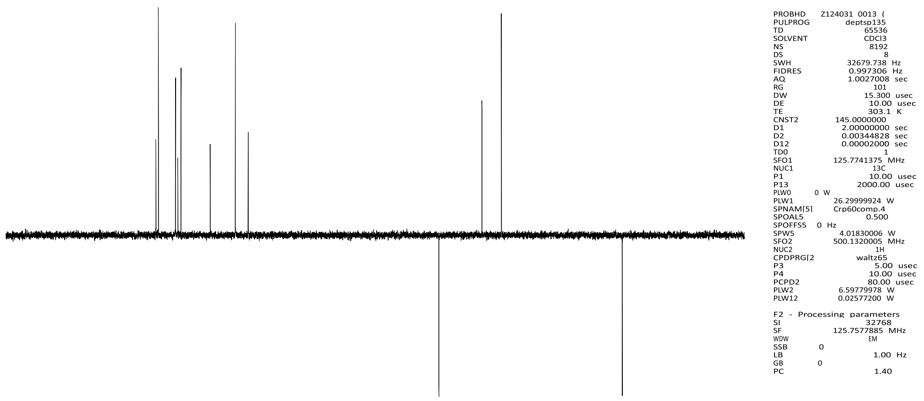

S55. Comp. 23

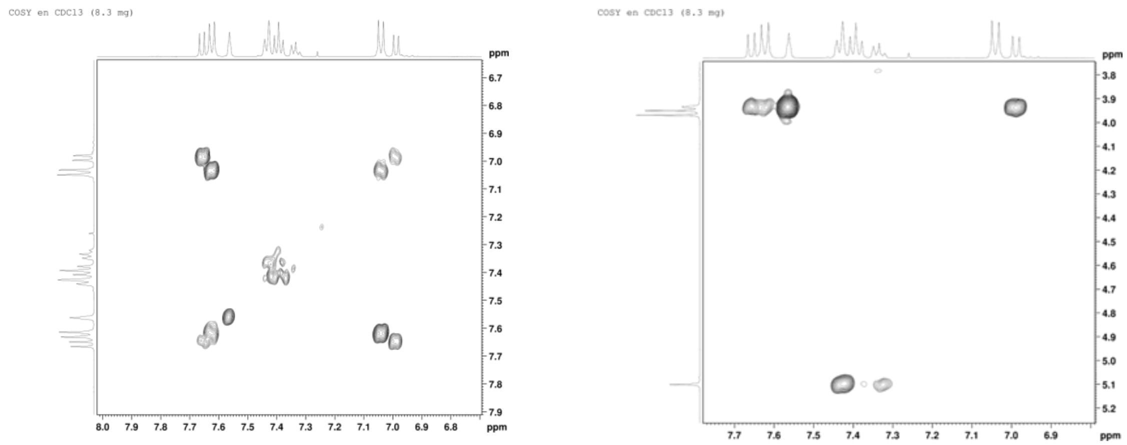

S56. Comp. 23

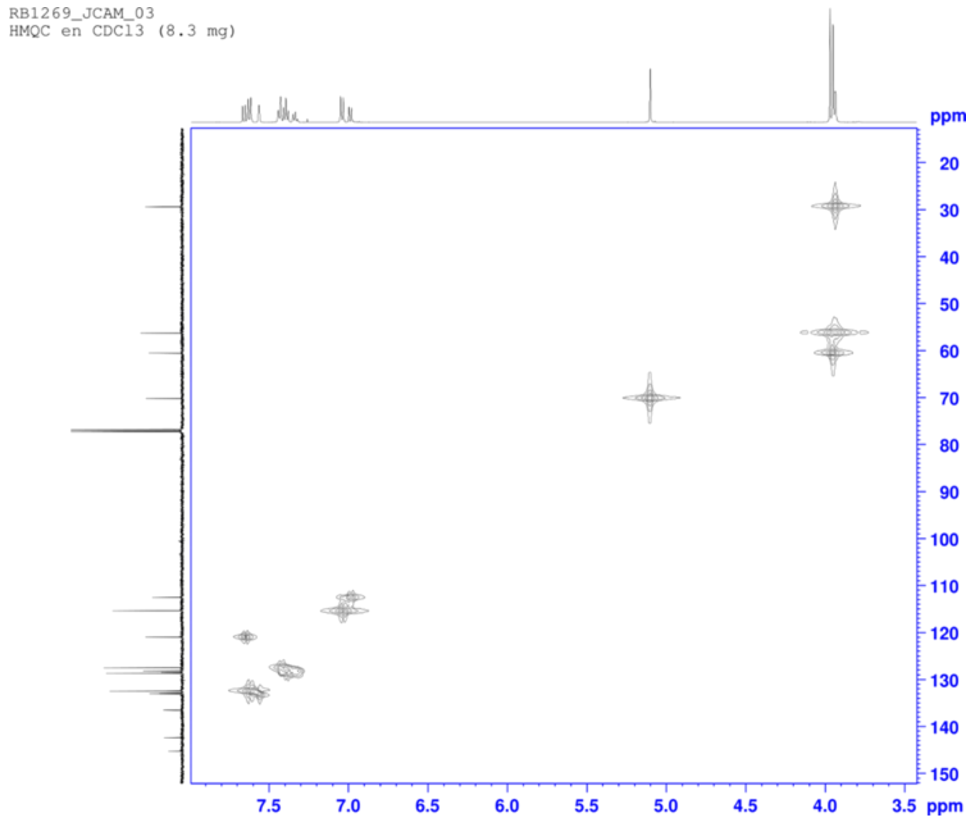

S57. Comp. 23

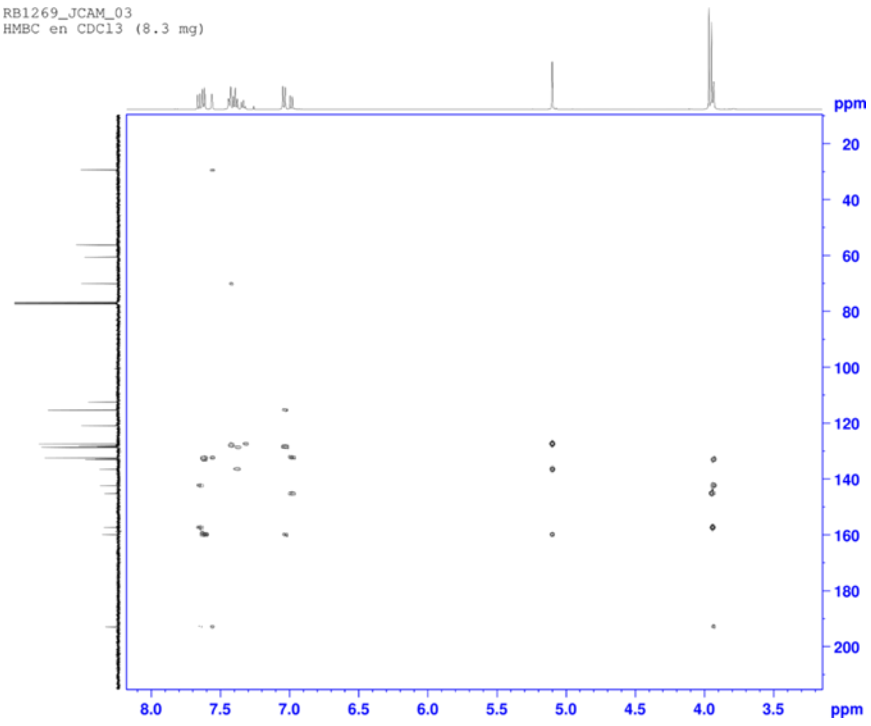

S58. Comp. 24

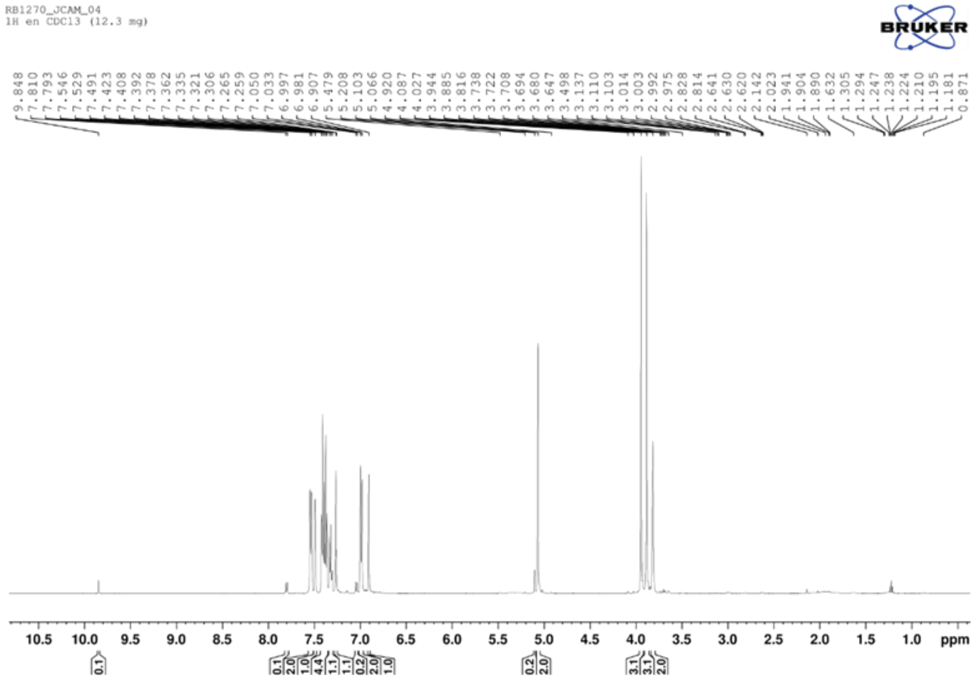

S59. Comp. 24

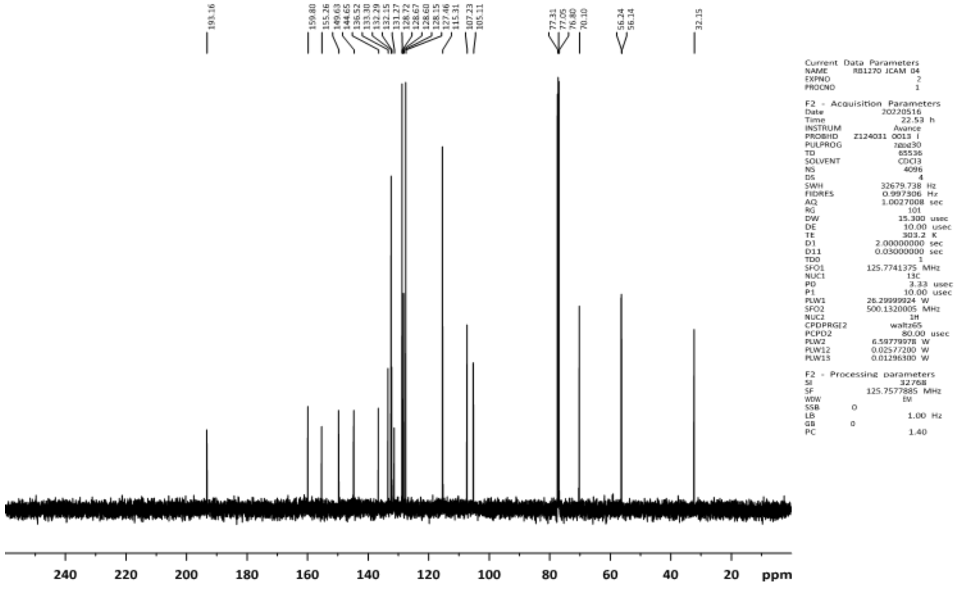

S60. Comp. 24

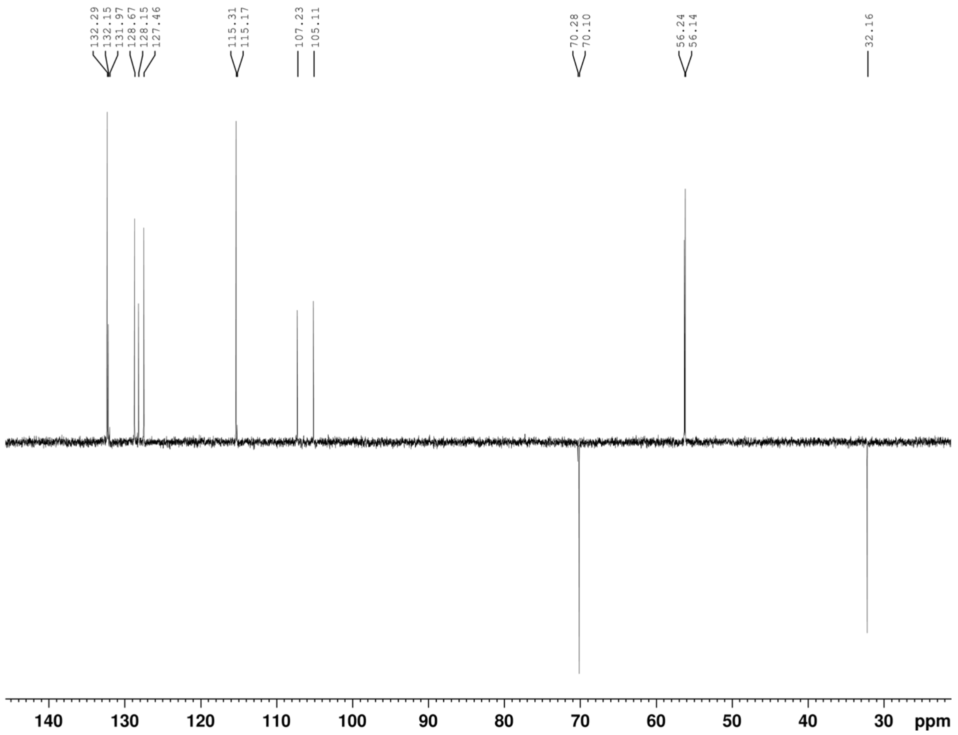

S61. Comp. 24

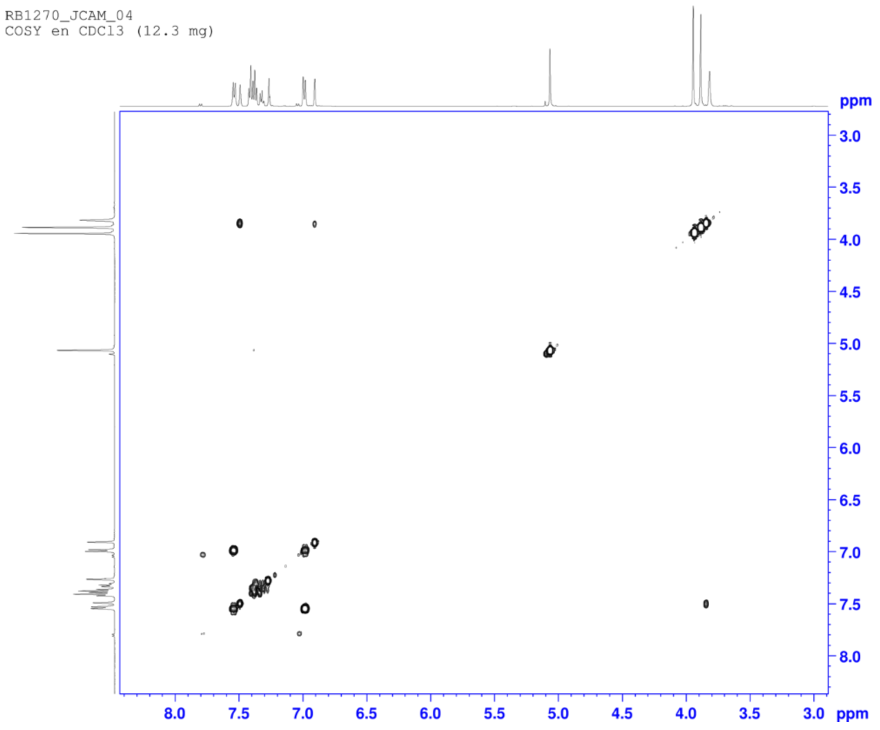

S62. Comp. 24

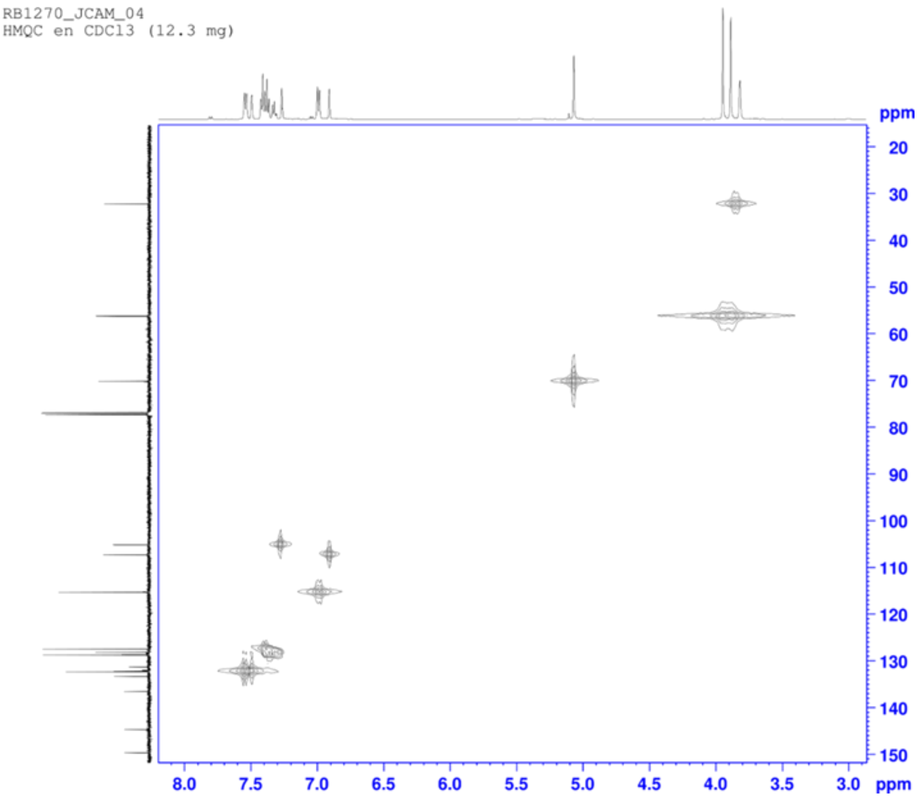

S63. Comp. 24

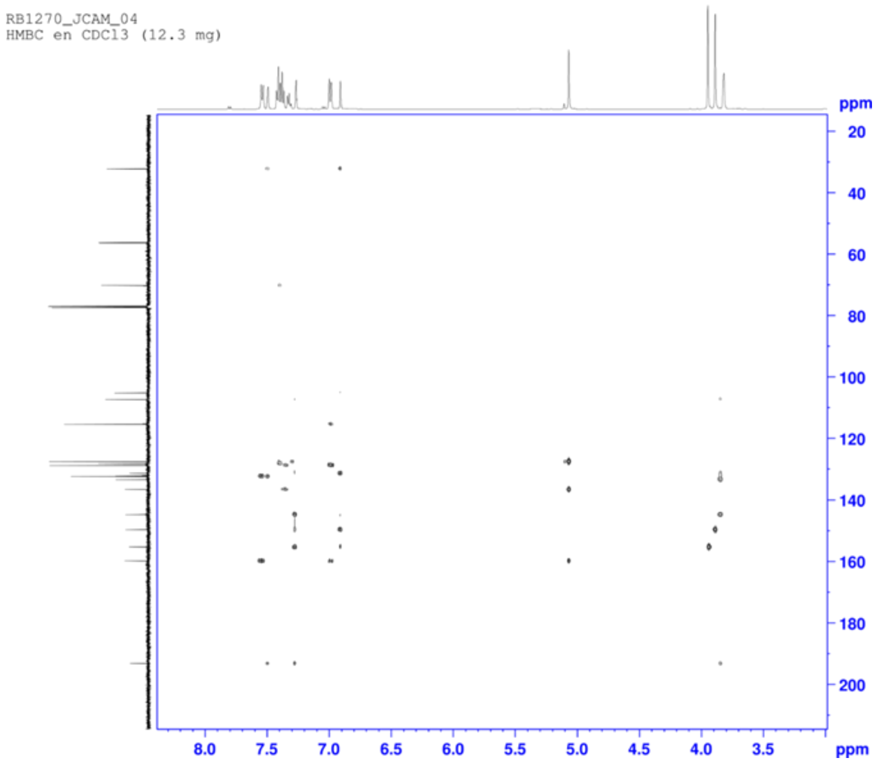

S64. Comp. 25

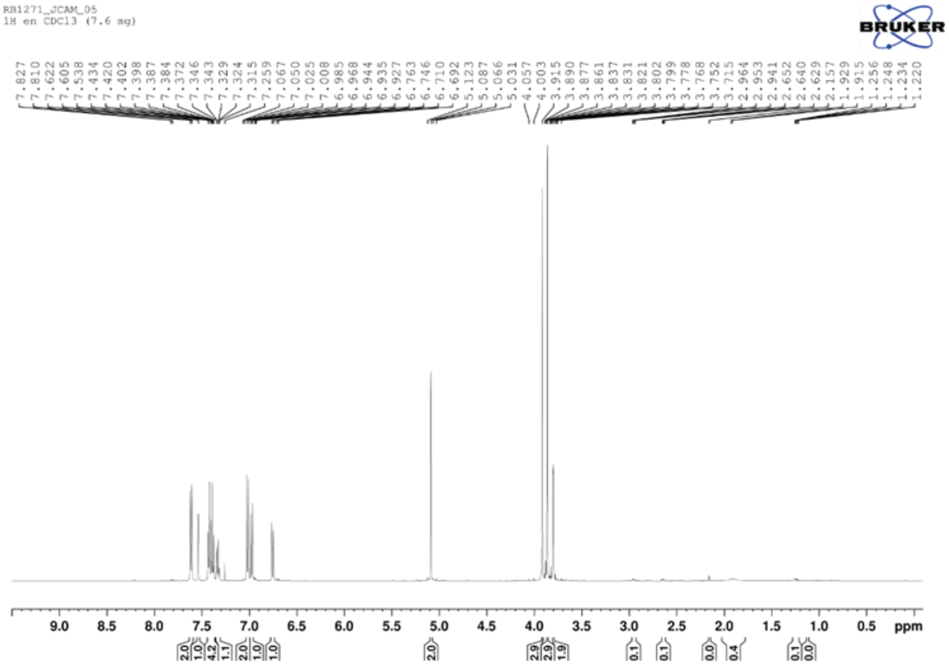

S65. Comp. 25

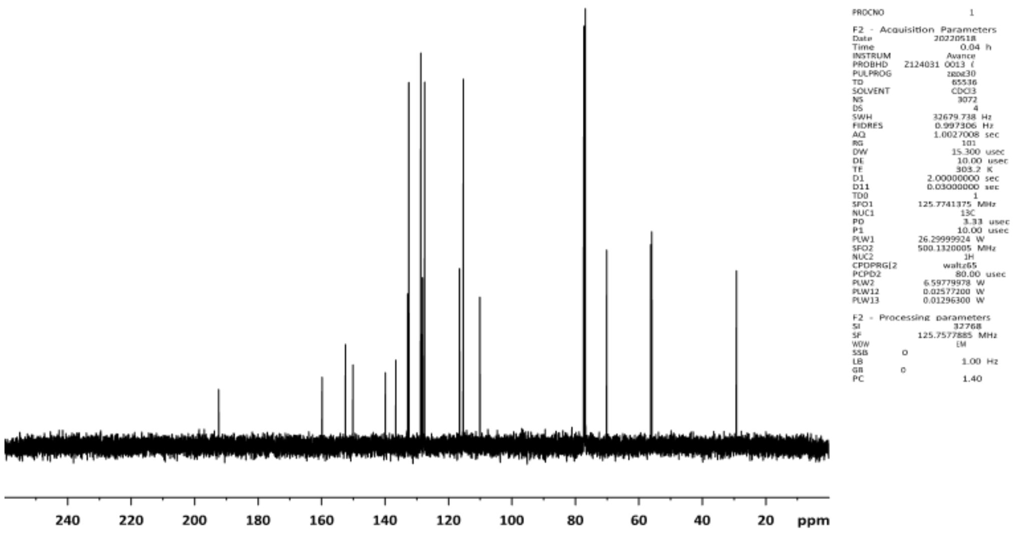

S66. Comp. 25

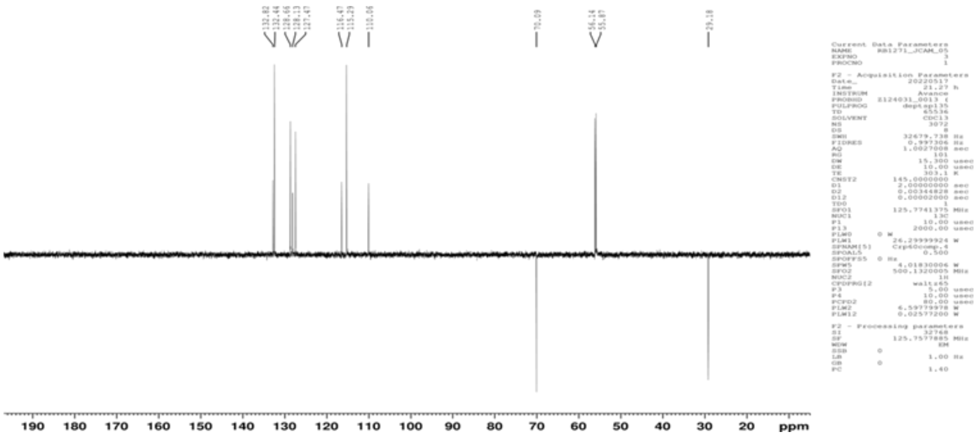

S67. Comp. 25

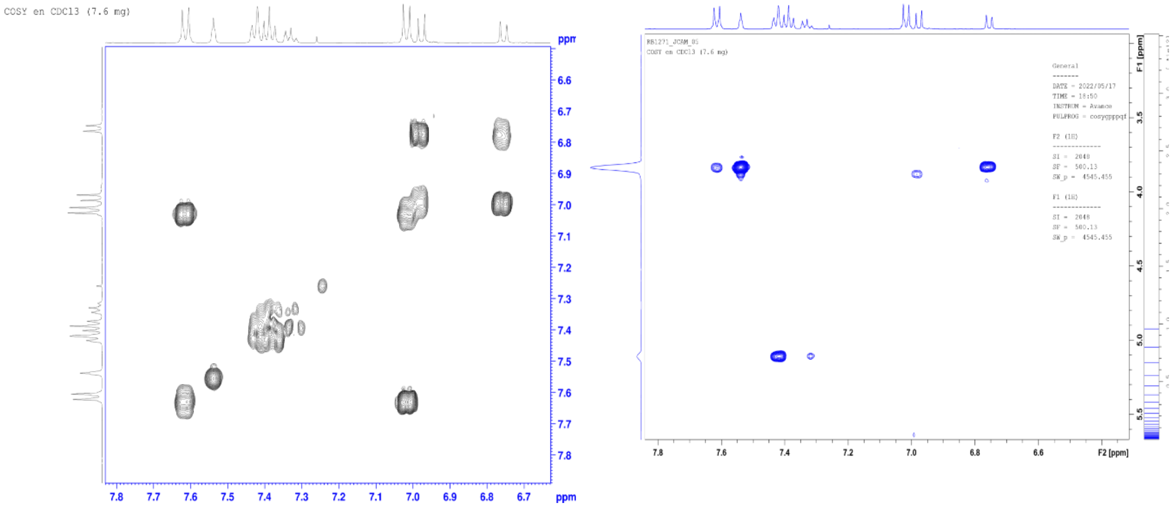

S68. Comp. 25

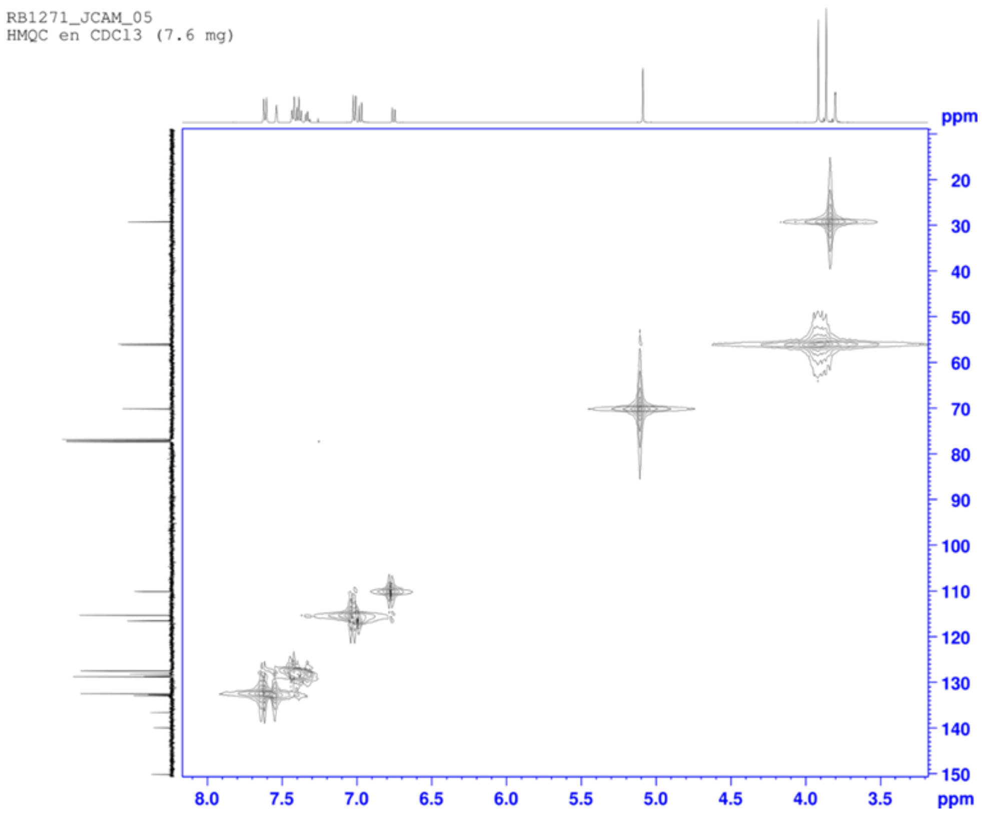

S69. Comp. 25

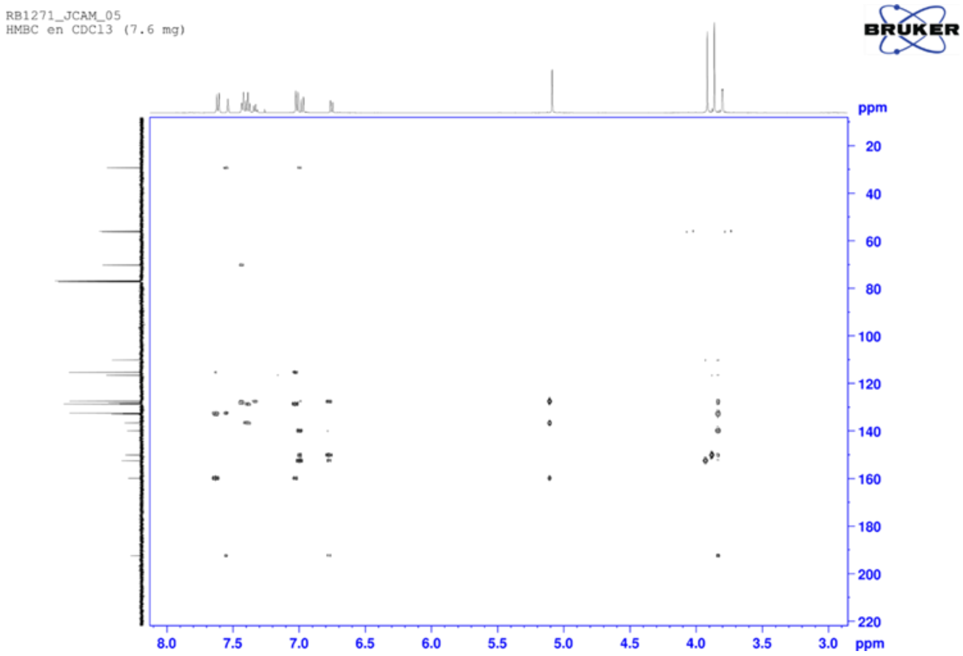

S70. Comp. 27

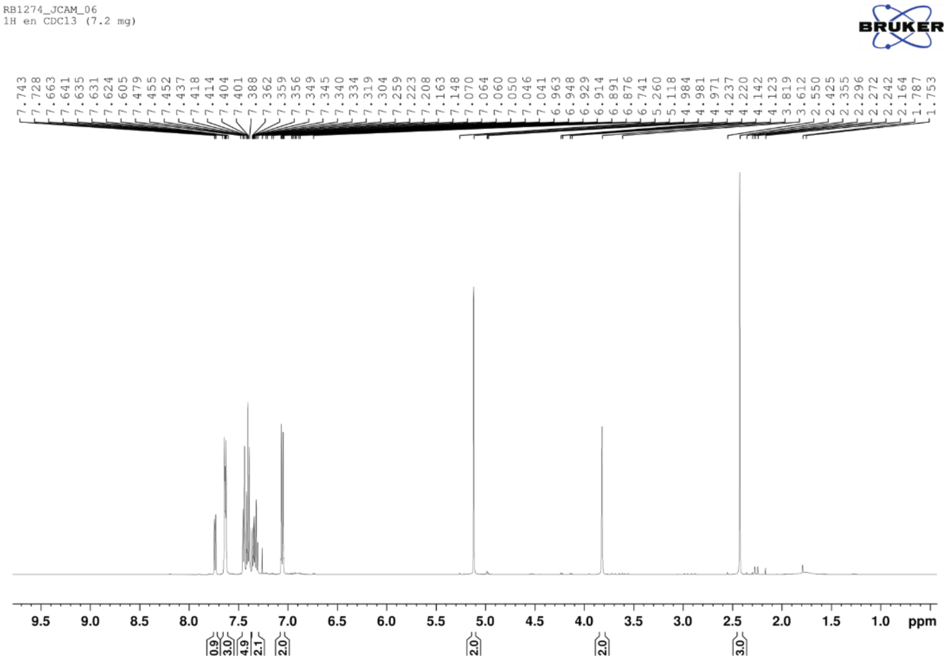

S71. Comp. 27

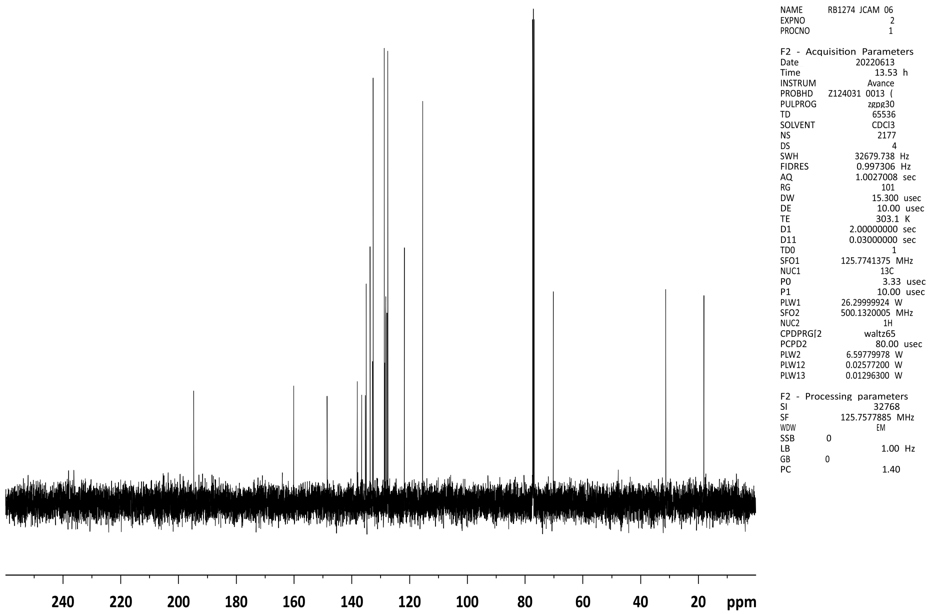

S72. Comp. 27

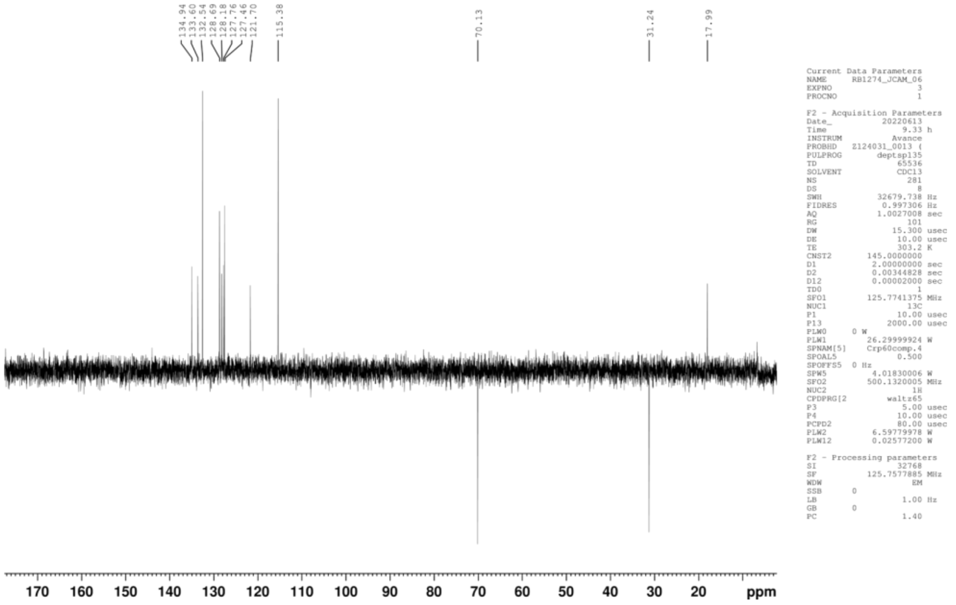

S73. Comp. 27

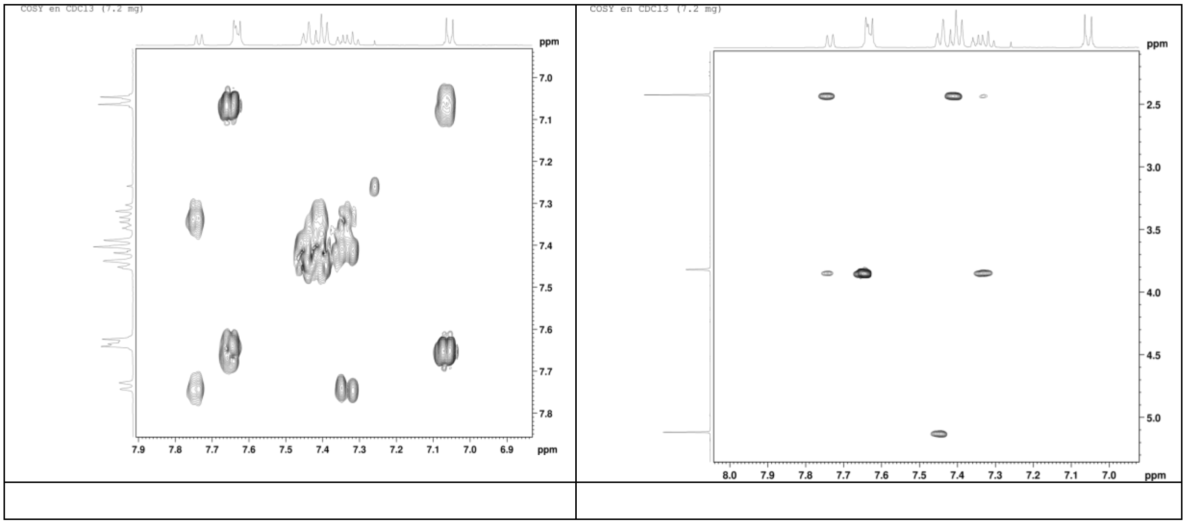

S74. Comp. 27

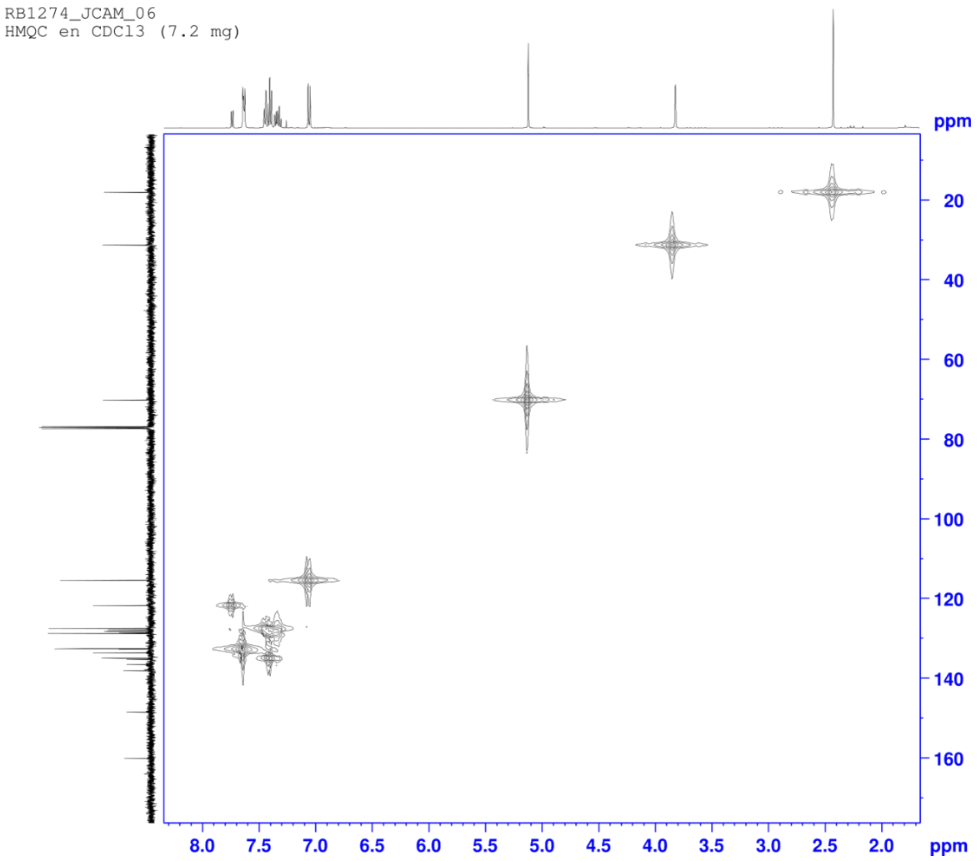

S75. Comp. 27

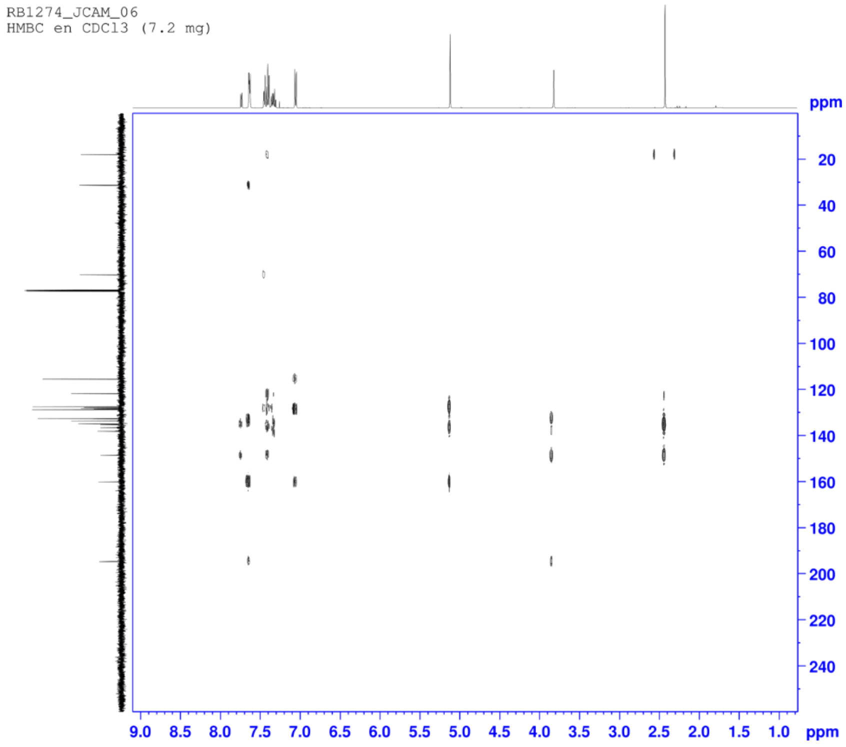

S76. Comp. 30

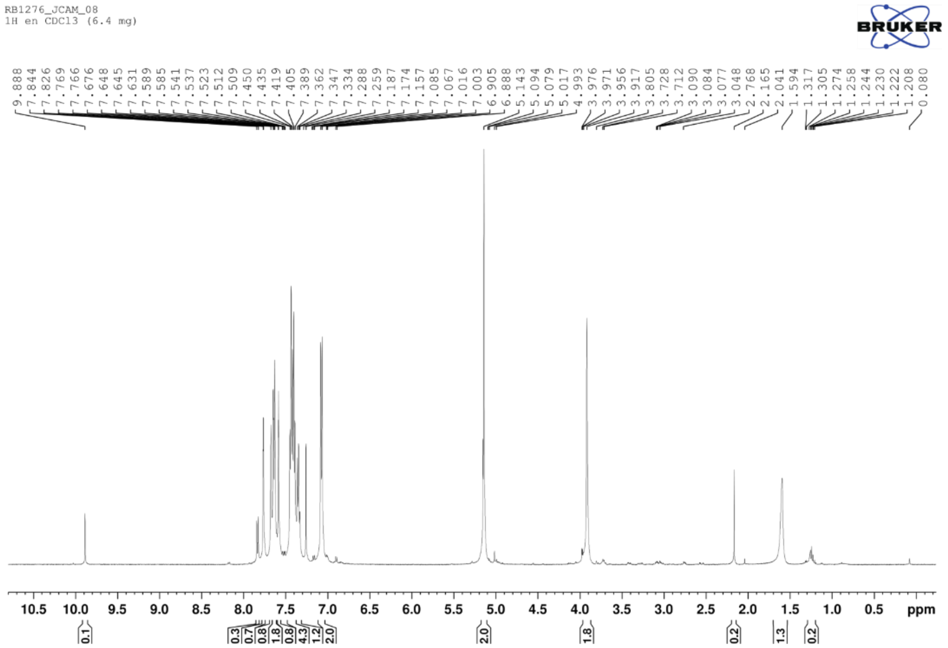

S77. Comp. 30

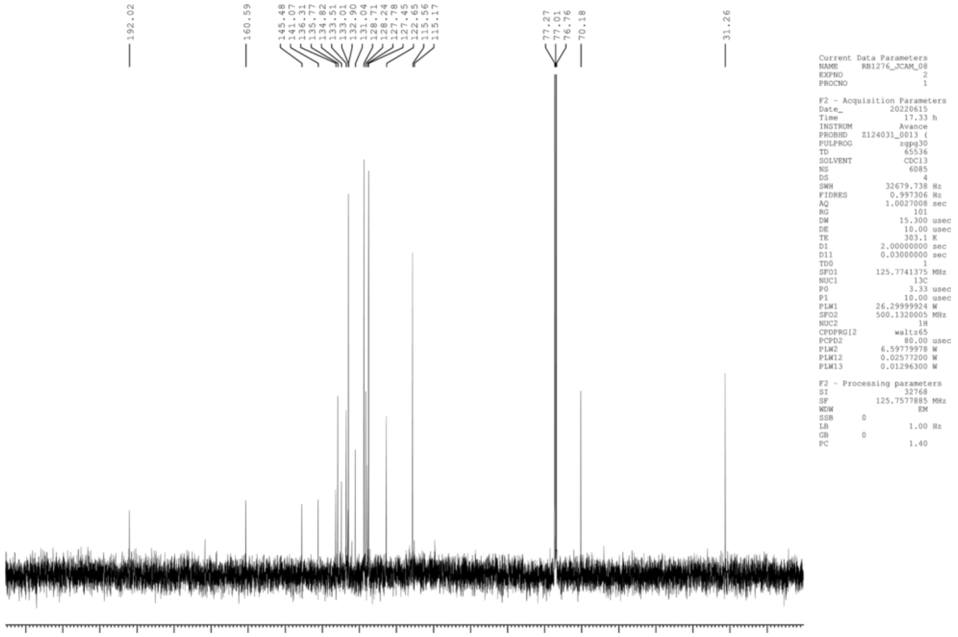

S78. Comp.31

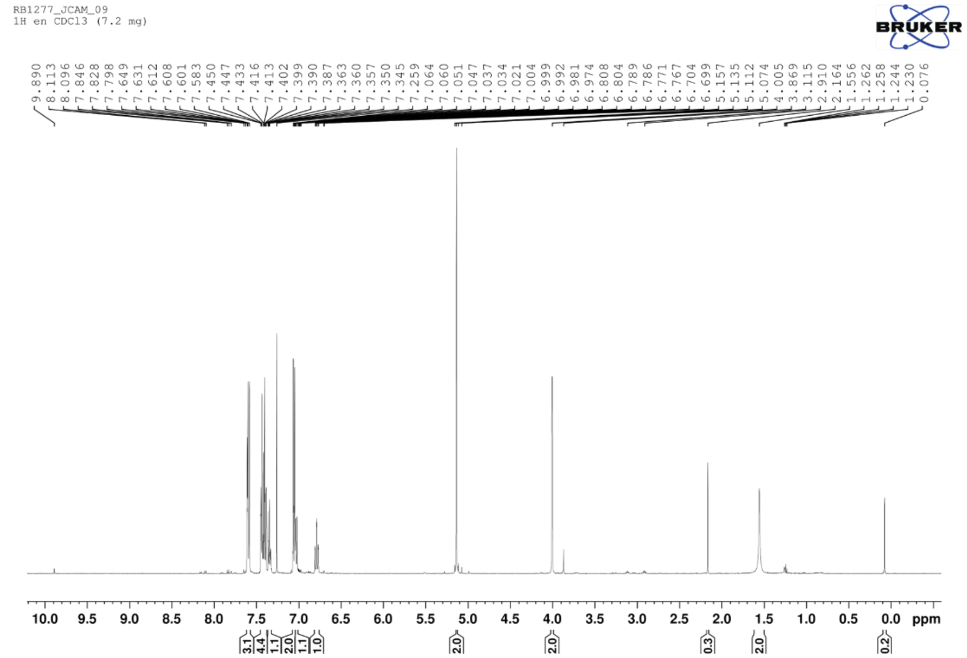

S79. Comp. 31

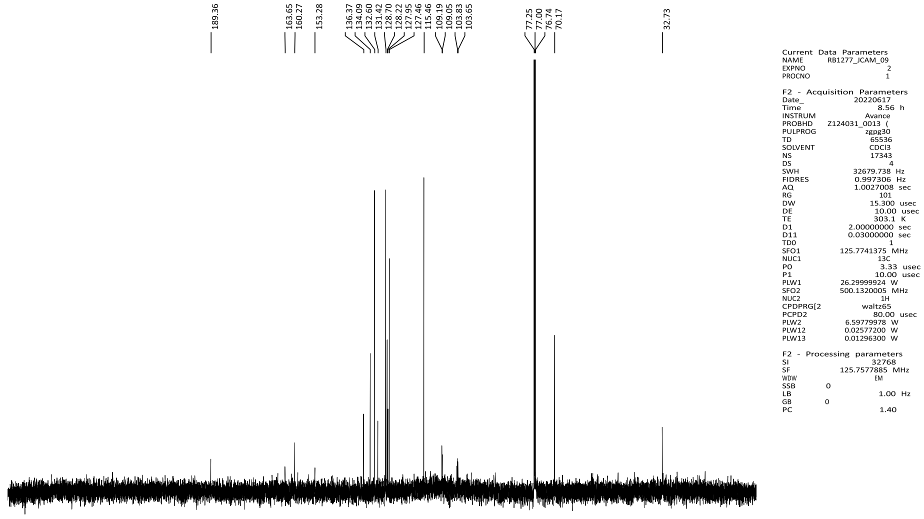

S80. Comp. 31

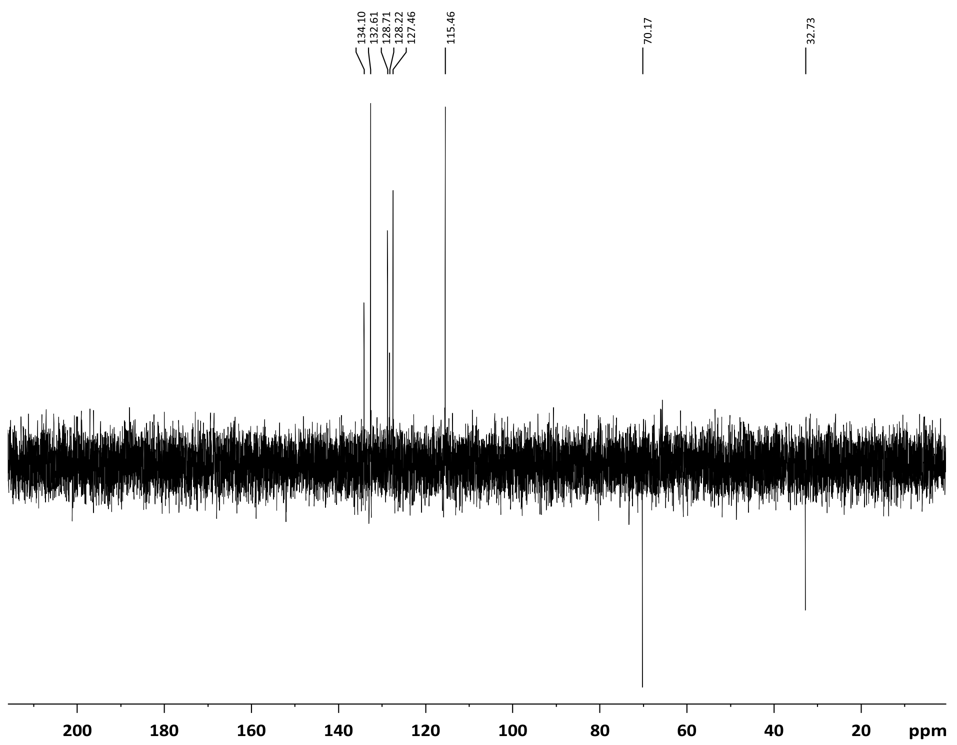

S81. Comp. 31

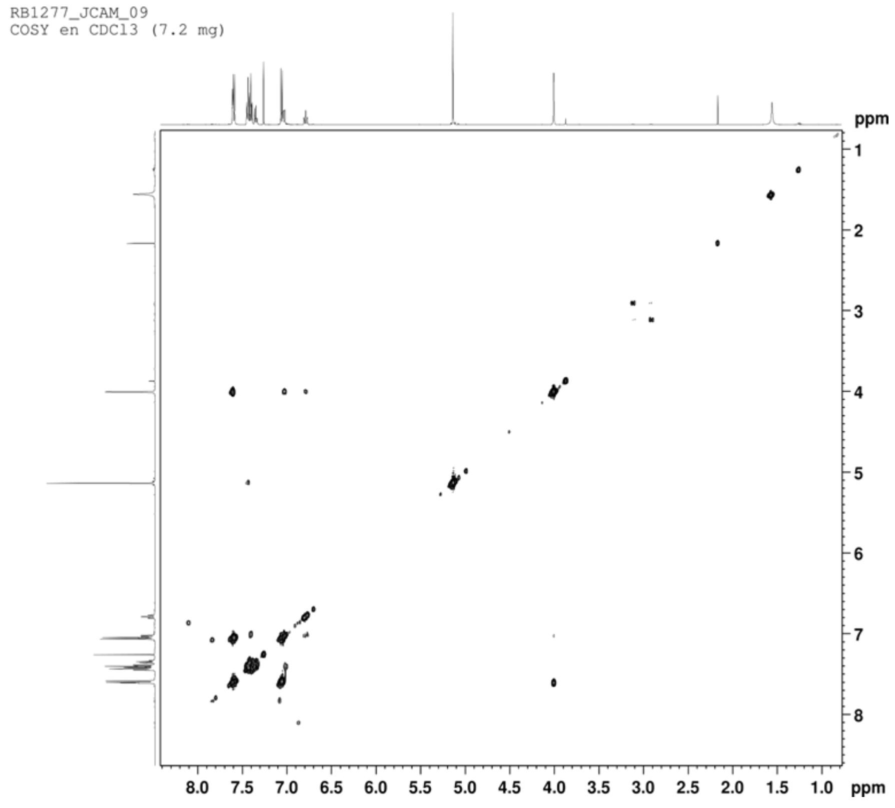

S82. Comp. 31

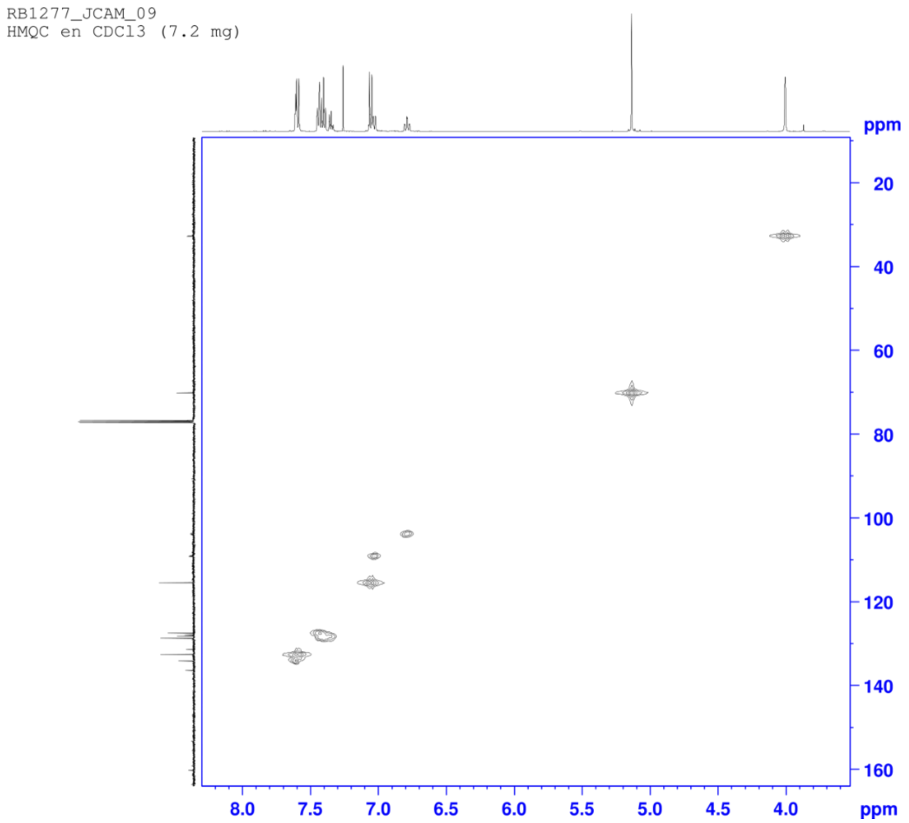

S83. Comp. 31

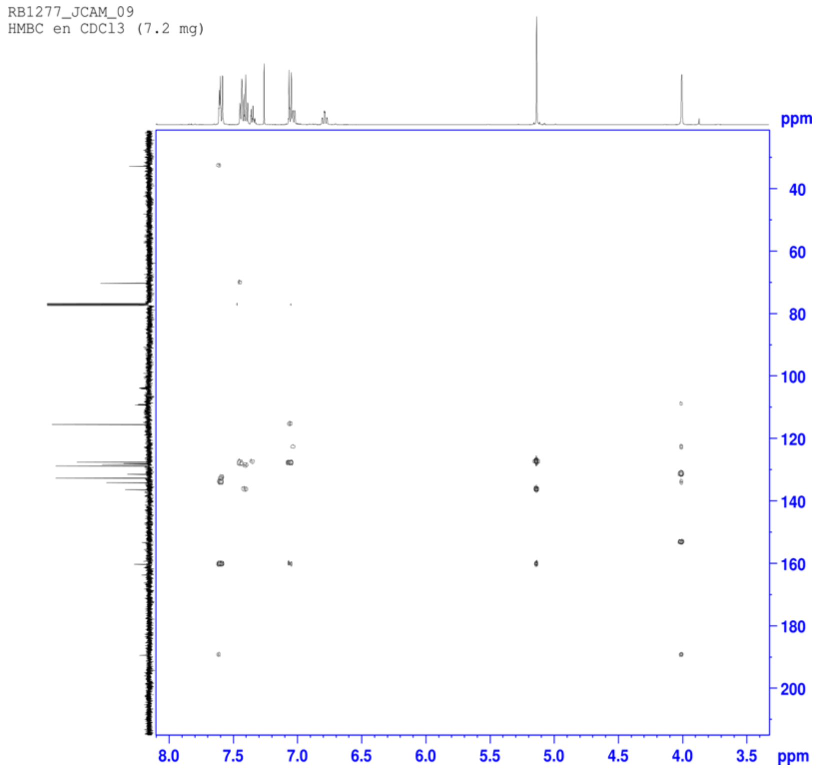

S84. Comp. 32

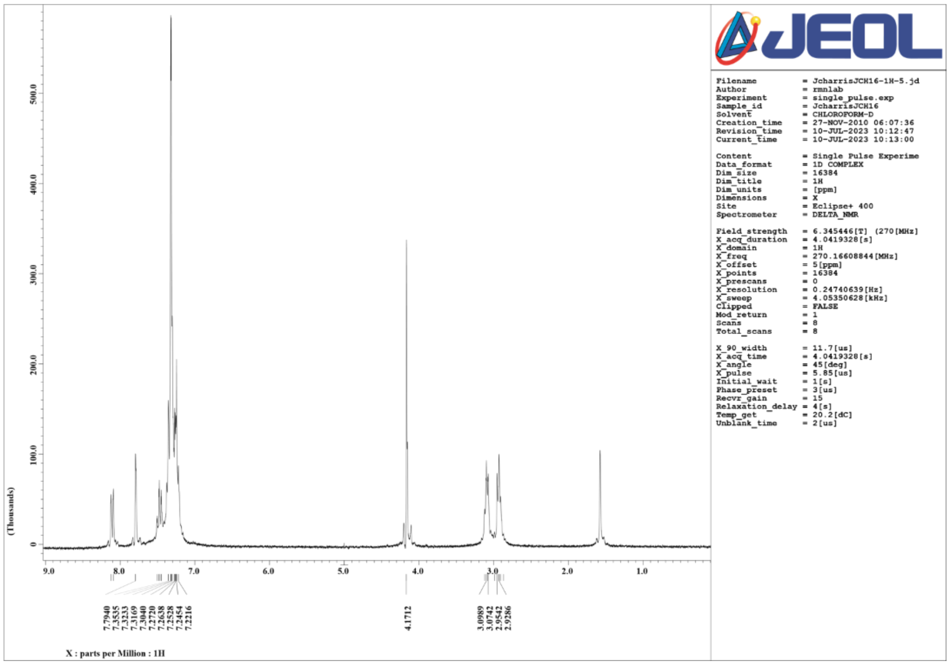

S85. Comp. 32

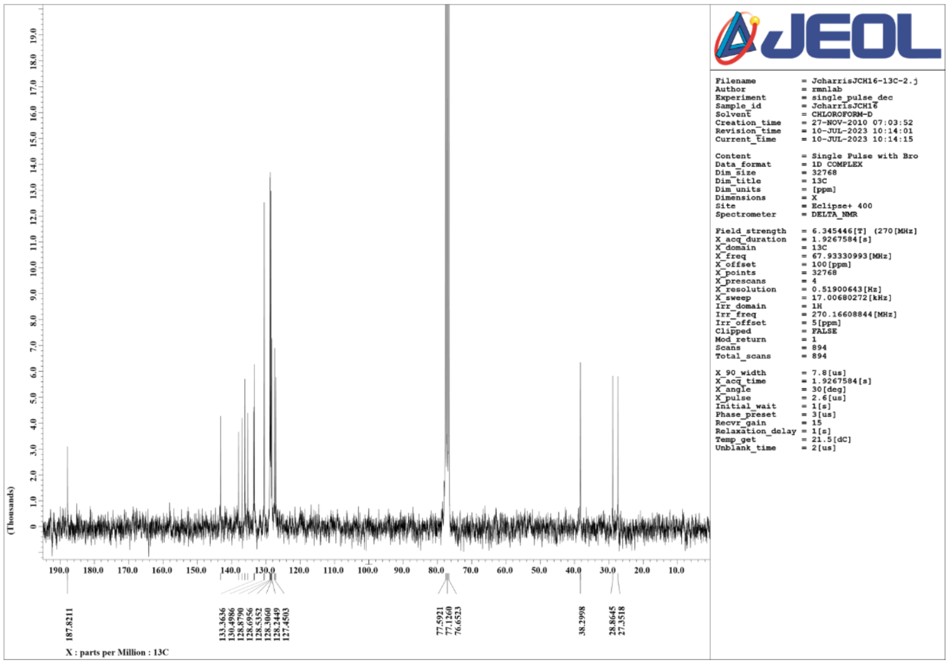

S86. Comp. 32

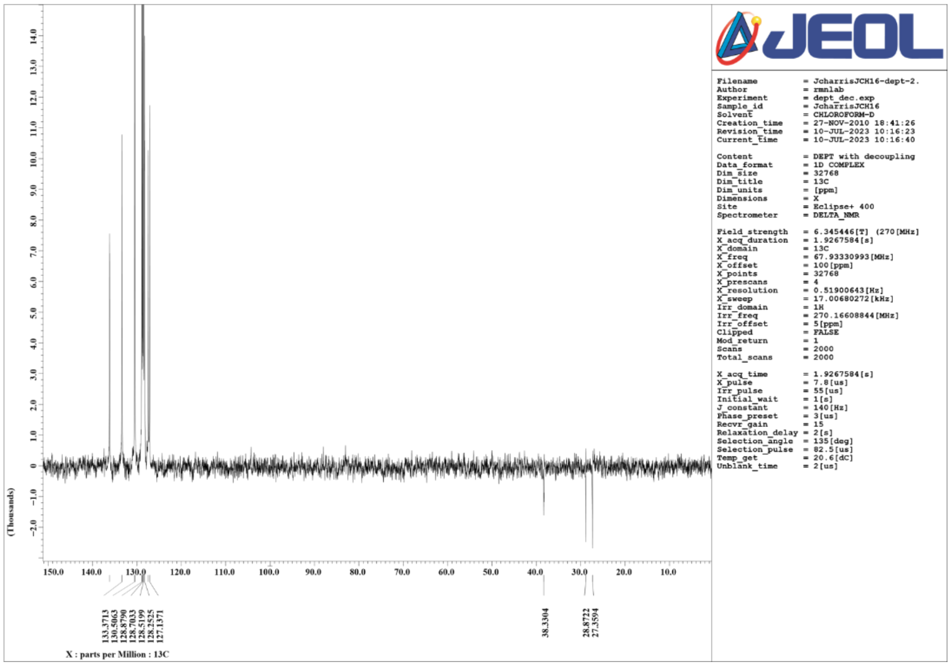

S87. Comp. 32

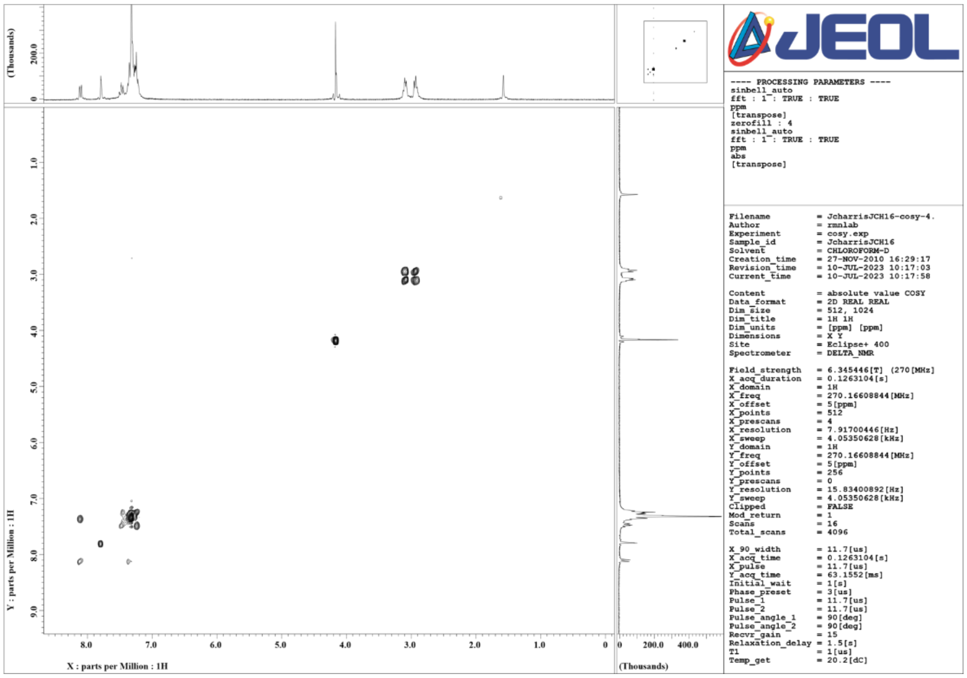

S88. Comp. 32

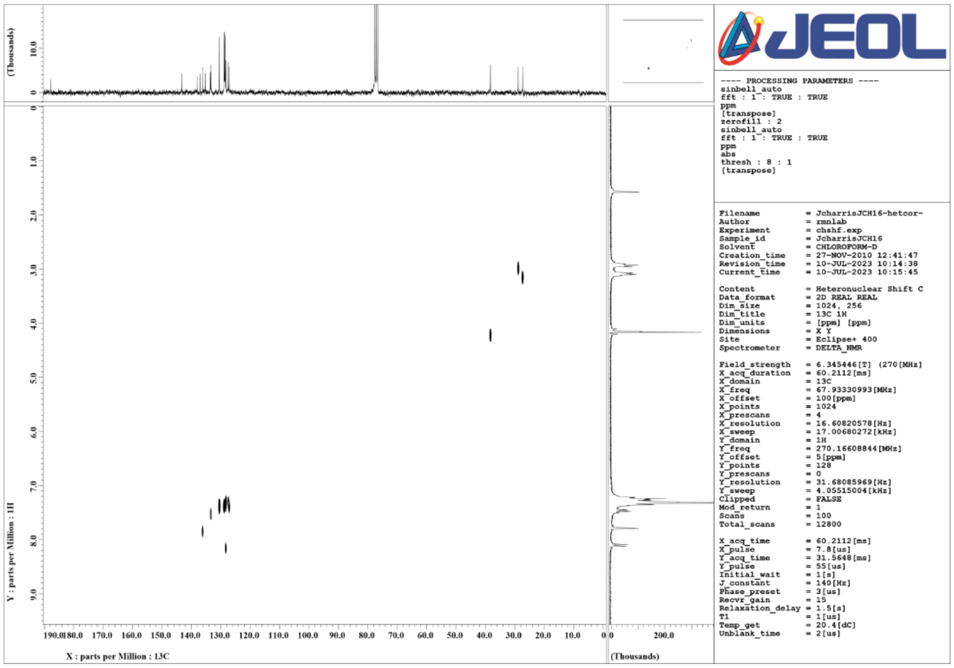
(Thousands)
10.0
0
Y : parts per Million : 1H
1.0
2.0
3.0
4.0
5.0
6.0
7.0
8.0
9.0
190.0 180.0 170.0 160.0 150.0 140.0 130.0 120.0 110.0 100.0 90.0 80.0 70.0 60.0 50.0 40.0 30.0 20.0 10.0 0
X : parts per Million : 13C
0 200.0
(Thousands)
---- PROCESSING PARAMETERS ----
sinbell_auto
fft : 1 : TRUE : TRUE
ppm
[transpose]
zerofill : 2
sinbell_auto
fft : 1 : TRUE : TRUE
ppm
abs
thresh : 8 : 1
[transpose]
Filename = JcharrisJCH16-hetcor-
Author = rmnlab
Experiment = chshf.exp
Sample_id = JcharrisJCH16
Solvent = CHLOROFORM-D
Creation_time = 27-NOV-2010 12:41:47
Revision_time = 10-JUL-2023 10:14:38
Current_time = 10-JUL-2023 10:15:45
Content = Heteronuclear Shift C
Data_format = 2D REAL REAL
Dim_size = 1024, 256
Dim_title = 13C 1H
Dim_units = [ppm] [ppm]
Dimensions = X Y
Site = Eclipse+ 400
Spectrometer = DELTA_NMR
Field_strength = 6.345446[T] (270[MHz]
X_acq_duration = 60.2112[ms]
X_domain = 13C
X_freq = 67.93330993[MHz]
X_offset = 100[ppm]
X_points = 1024
X_prescans = 4
X_resolution = 16.60820578[Hz]
X_sweep = 17.00680272[kHz]
Y_domain = 1H
Y_freq = 270.16608844[MHz]
Y_offset = 5[ppm]
Y_points = 128
Y_prescans = 0
Y_resolution = 31.68085969[Hz]
Y_sweep = 4.05515004[kHz]
Clipped = FALSE
Mod_return = 1
Scans = 100
Total_scans = 12800
X_acq_time = 60.2112[ms]
X_pulse = 7.8[us]
Y_acq_time = 31.5648[ms]
Y_pulse = 55[us]
Initial_wait = 1[s]
J_constant = 140[Hz]
Phase_preset = 3[us]
Recvr_gain = 15
Relaxation_delay = 1.5[s]
T1 = 1[us]
Temp_get = 20.4[dC]
Unblank_time = 2[us]

S89. Comp. 33

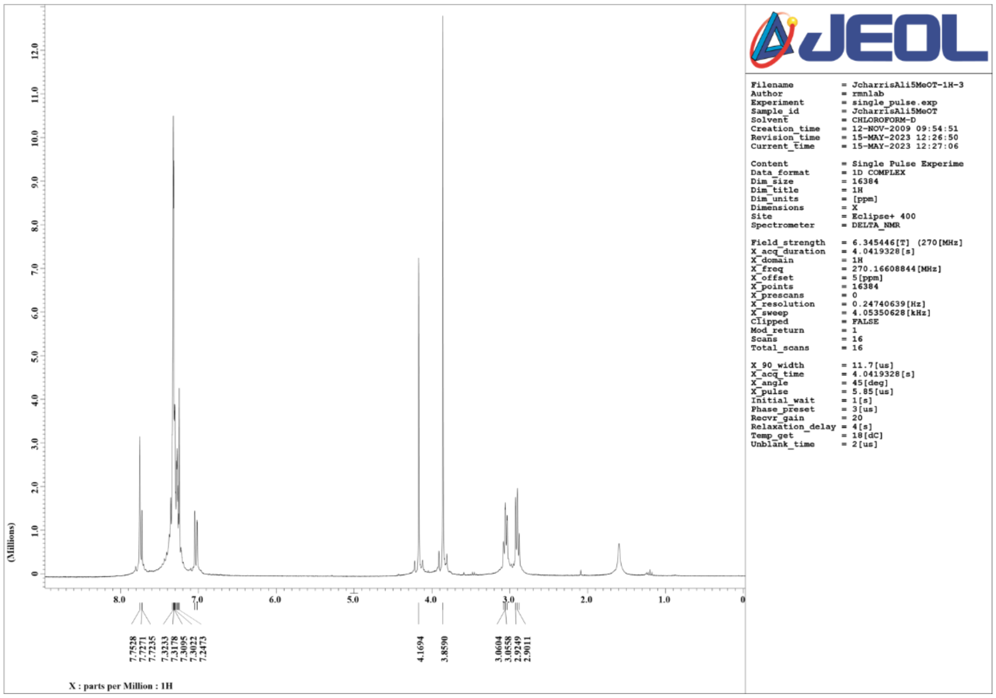

S90. Comp. 33

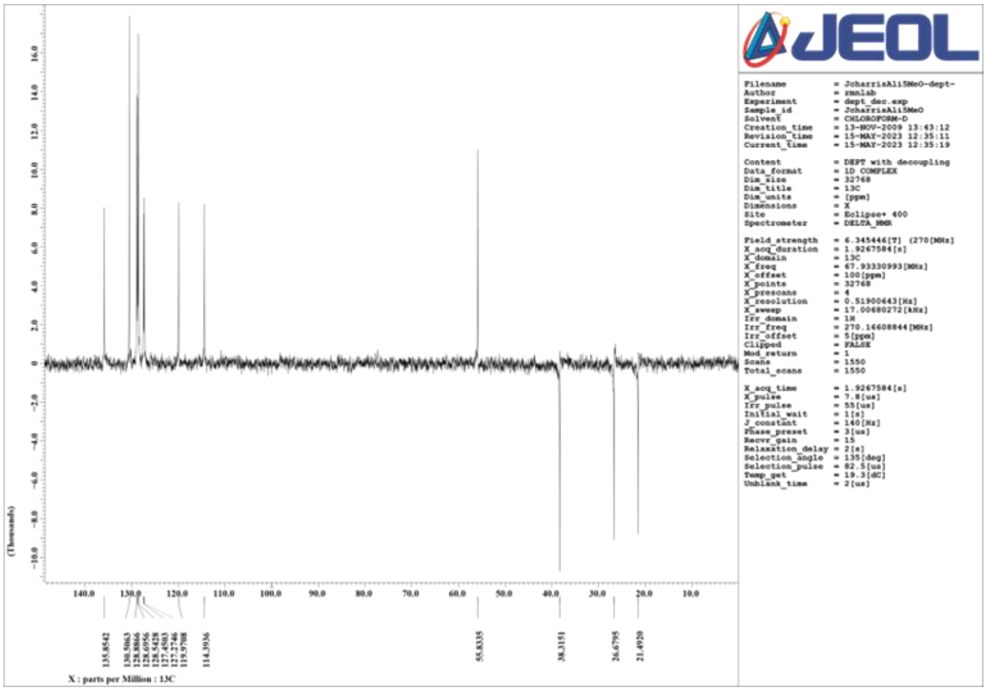

S91. Comp. 34

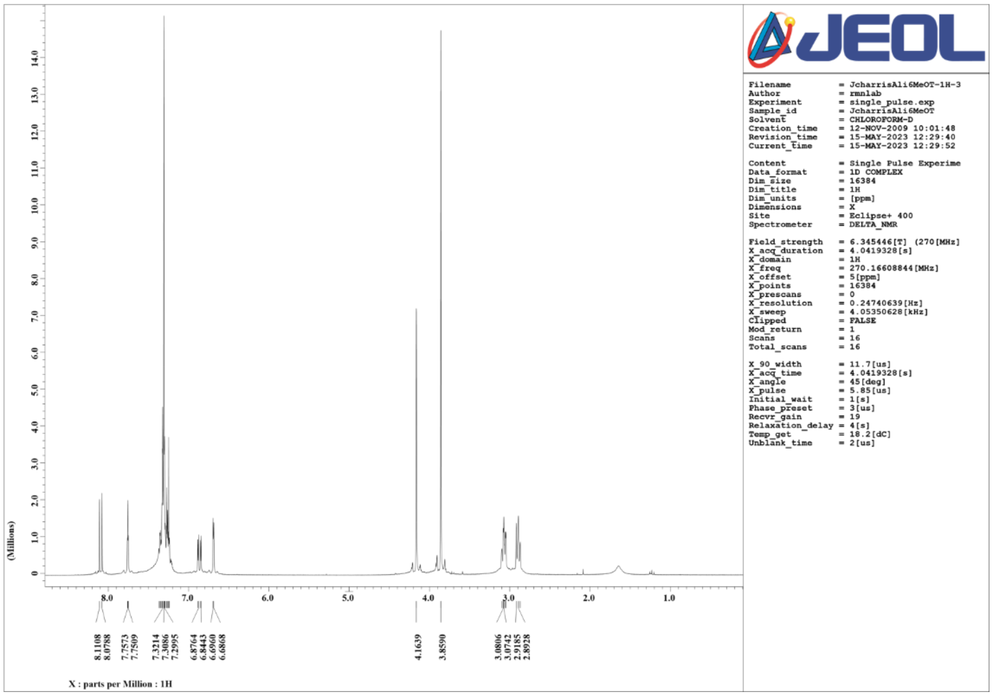

S92. Comp. 34

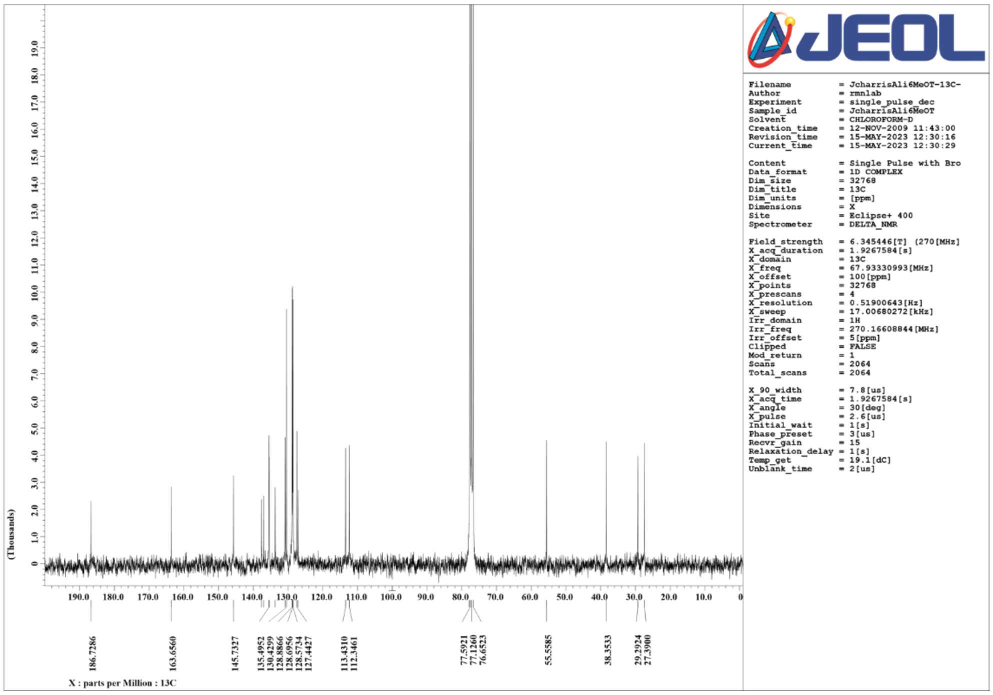

S93. Comp. 34

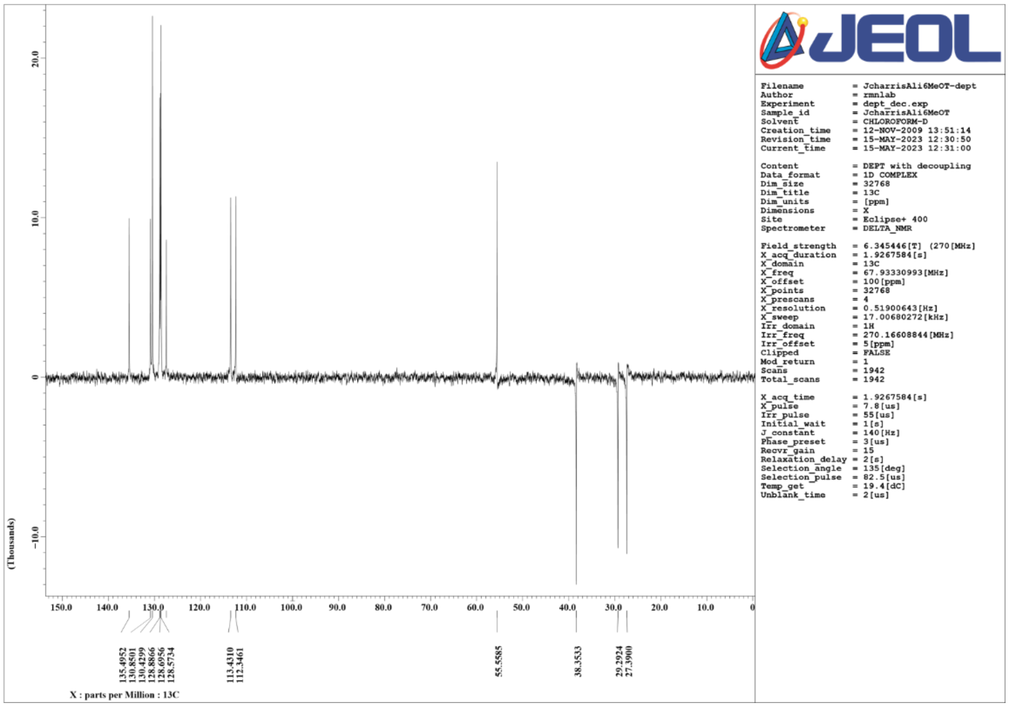

S94. Comp. 34

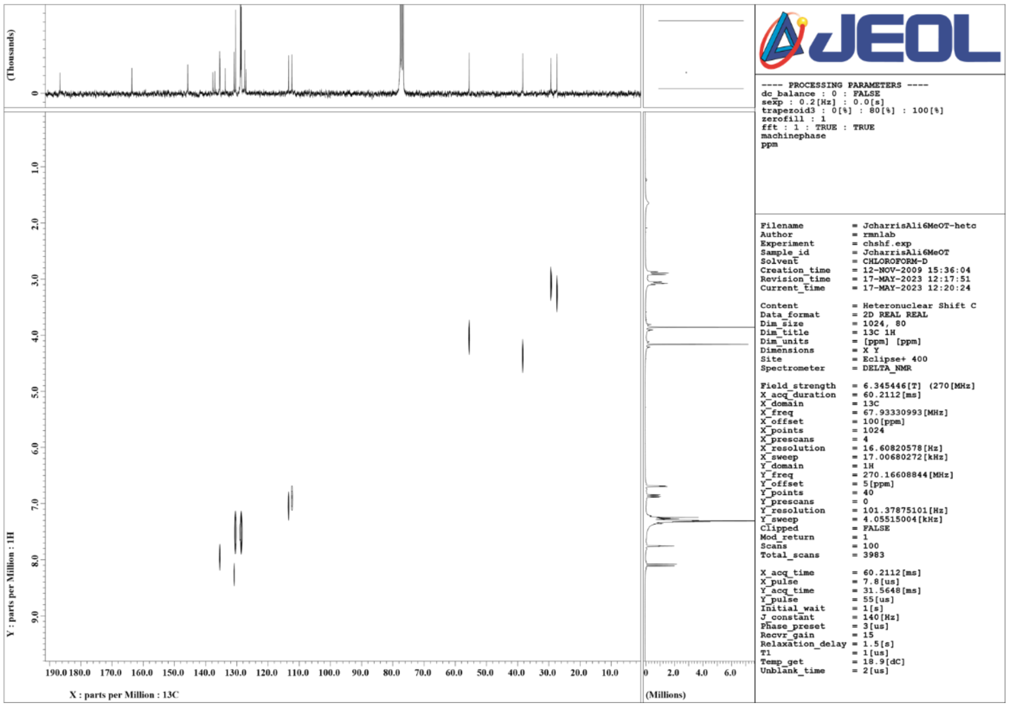

S95. Comp. 35

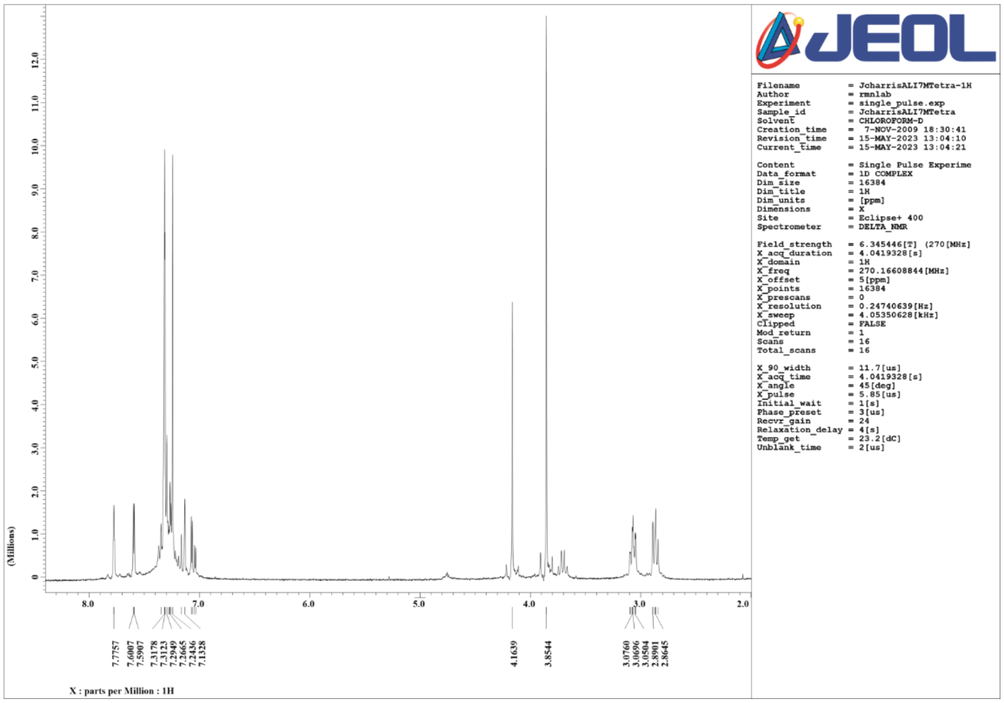

S96. Comp. 35

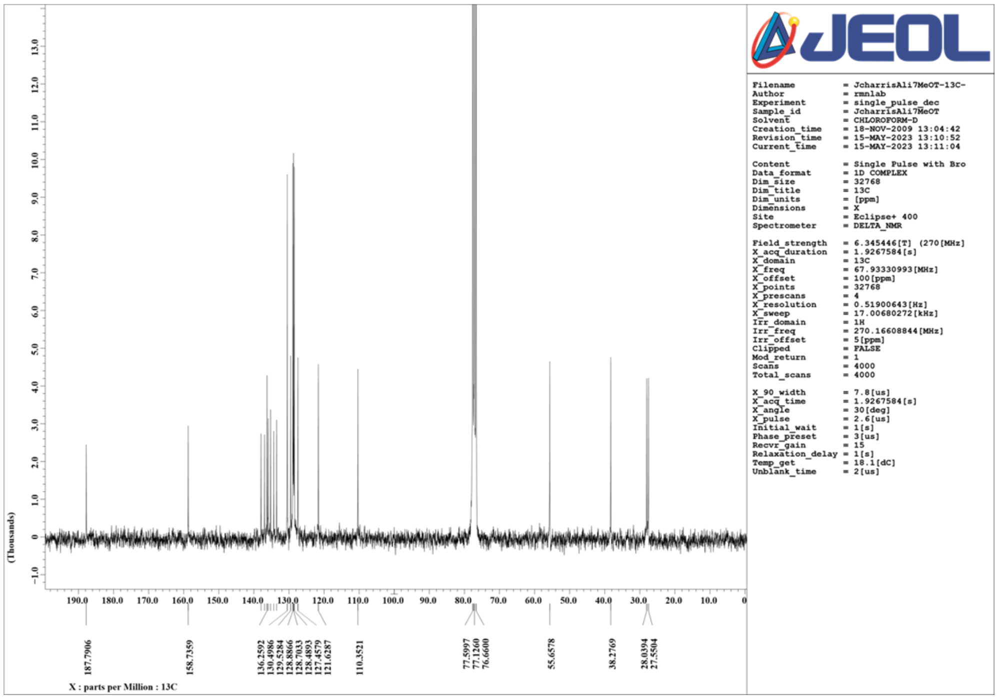

S97. Comp. 35

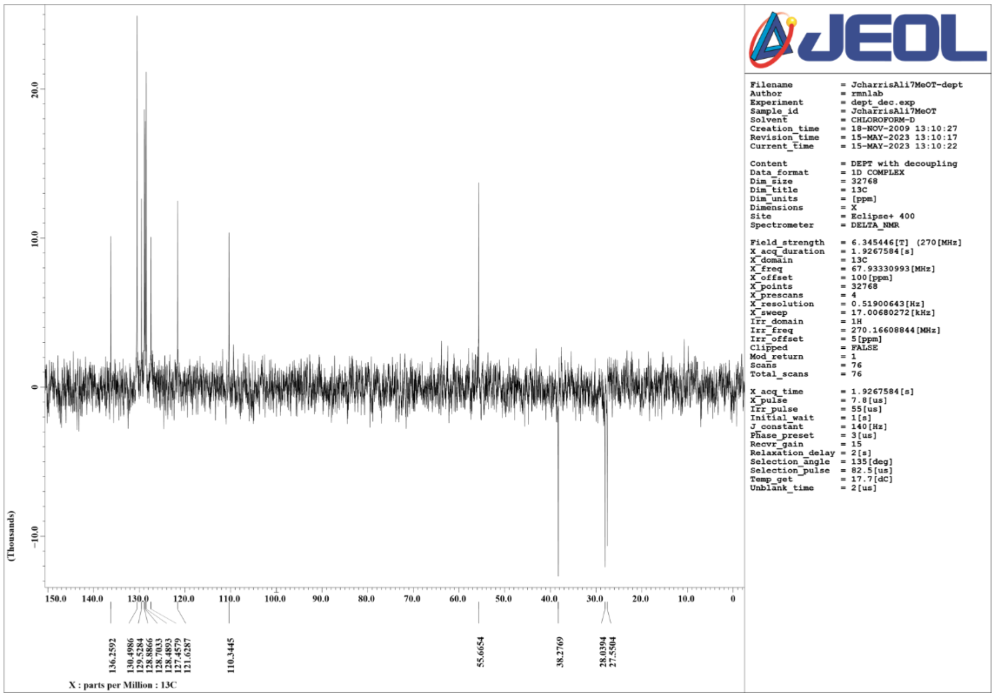

S98. Comp. 35

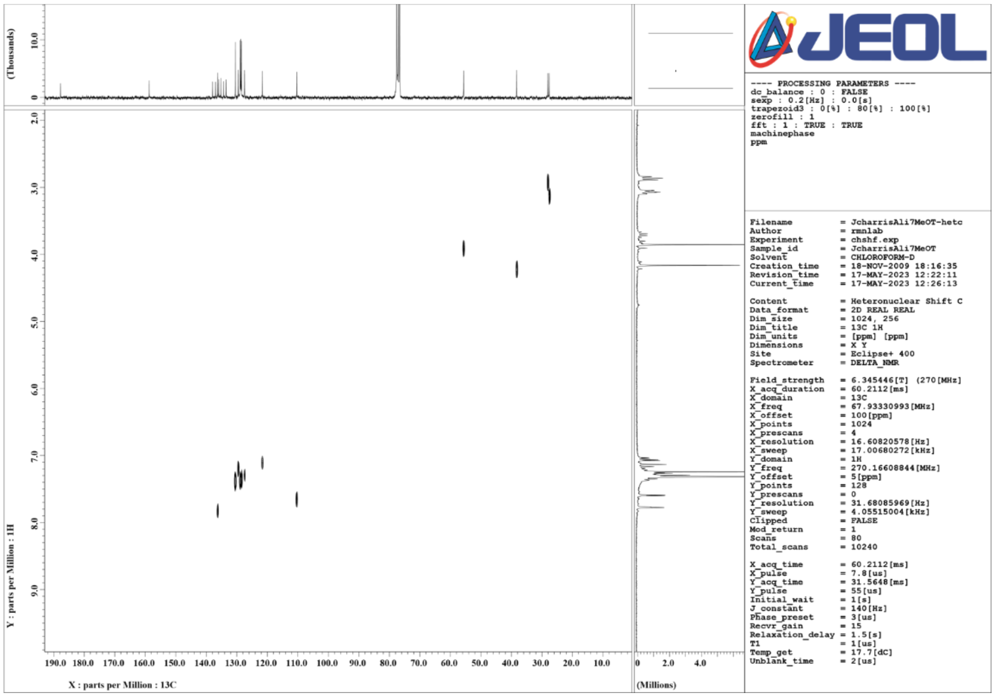

S99. Comp. 38

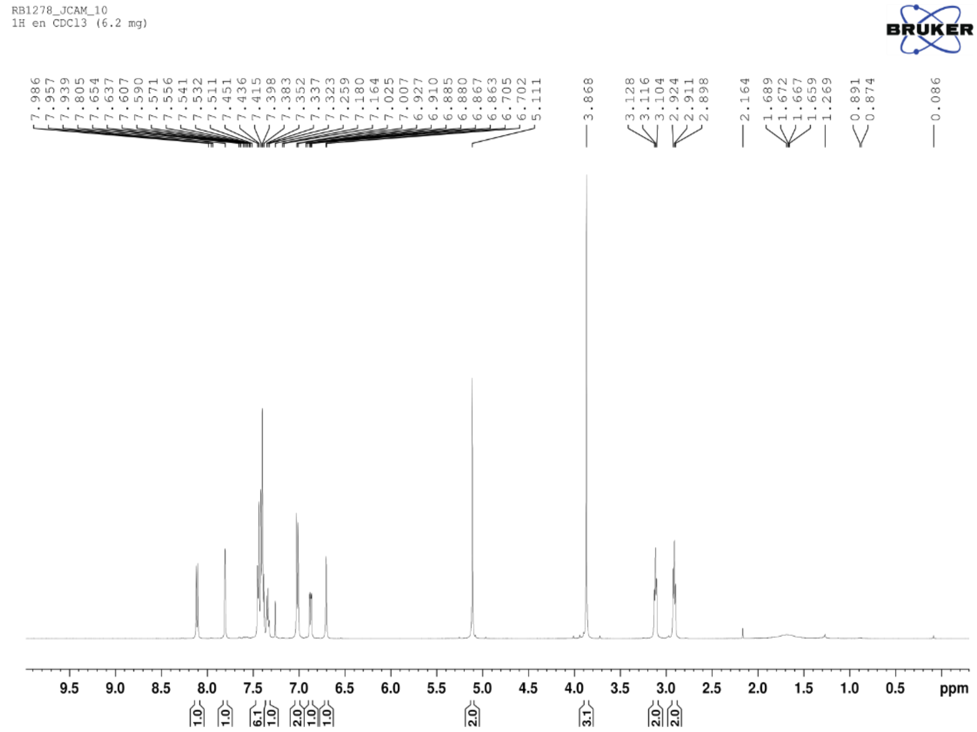

S100. Comp. 38

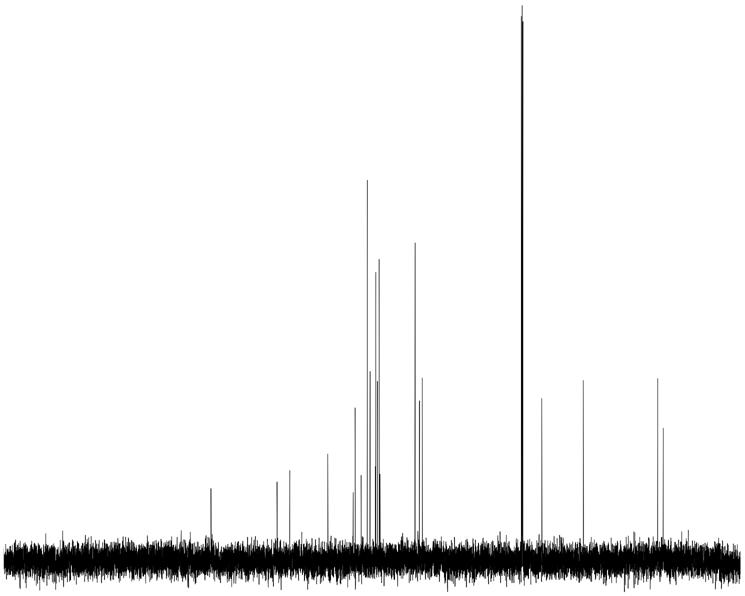

F2 - Acquisition Parameters

Date	20220711
Time	11.05 h
INSTRUM	Avance
PROBHD	Z124031 0013 (
PULPROG	zgpg30
TD	65536
SOLVENT	CDCl3
NS	1686
DS	4
SWH	32679.738 Hz
FIDRES	0.997306 Hz
AQ	1.0027008 sec
RG	101
DW	15.300 usec
DE	10.00 usec
TE	298.2 K
D1	2.00000000 sec
D11	0.03000000 sec
TD0	1
SFO1	125.7741375 MHz
NUC1	13C
P0	3.33 usec
P1	10.00 usec
PLW1	26.29999924 W
SFO2	500.1320005 MHz
NUC2	1H
CPDPRG[2	waltz65
PCPD2	80.00 usec
PLW2	6.59779978 W
PLW12	0.02577200 W
PLW13	0.01296300 W

F2 - Processing parameters

SI	32768
SF	125.7577885 MHz
WDW	EM
SSB	0
LB	1.00 Hz
GB	0
PC	1.40

S101. Comp. 38

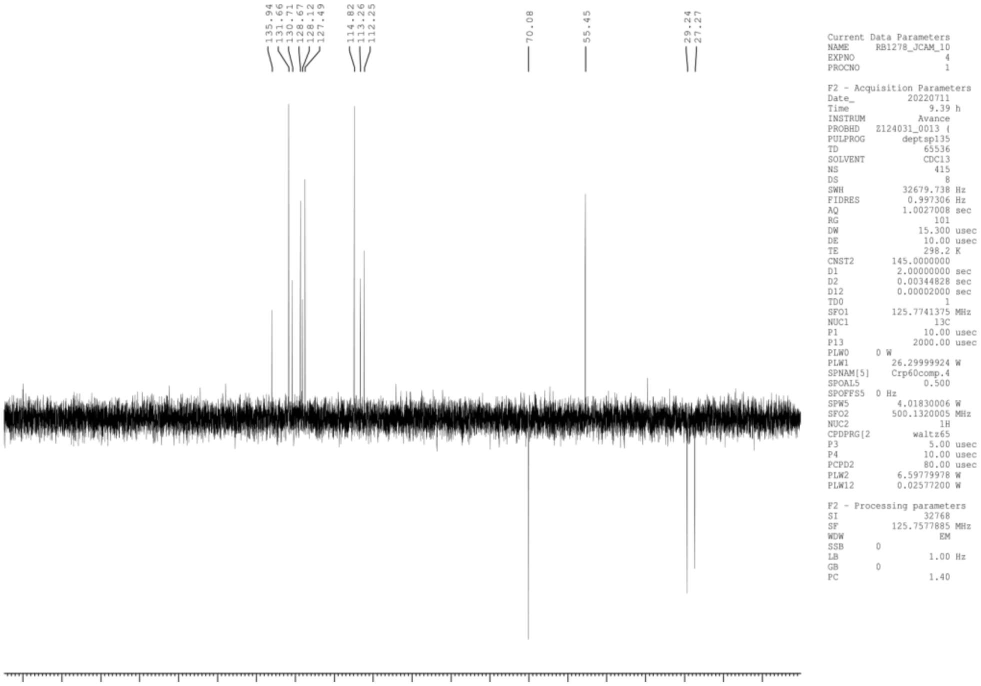

S102. Comp. 38

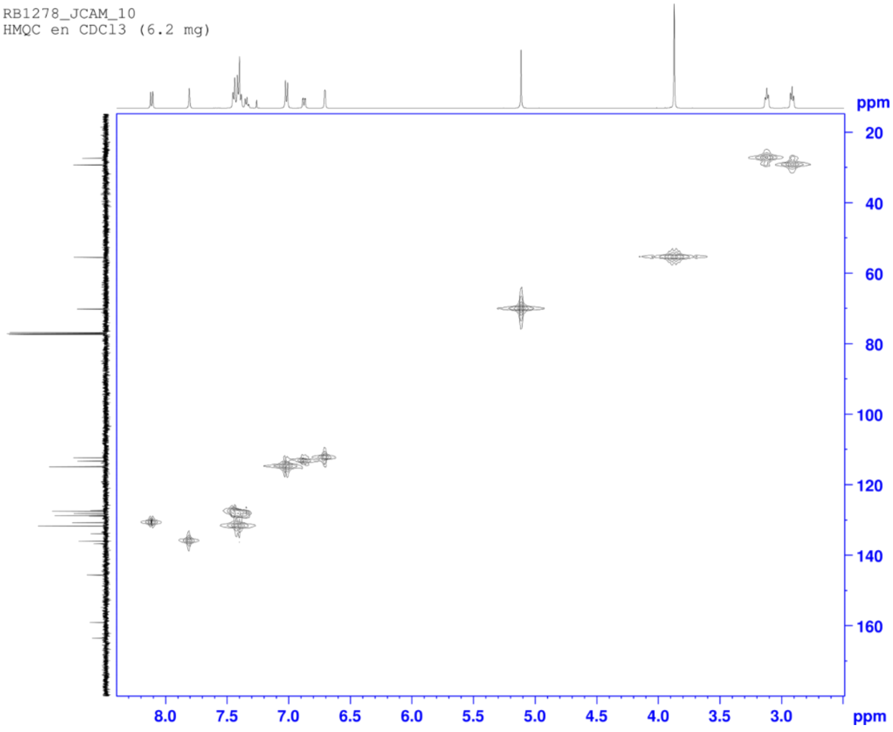

S103. Comp. 38

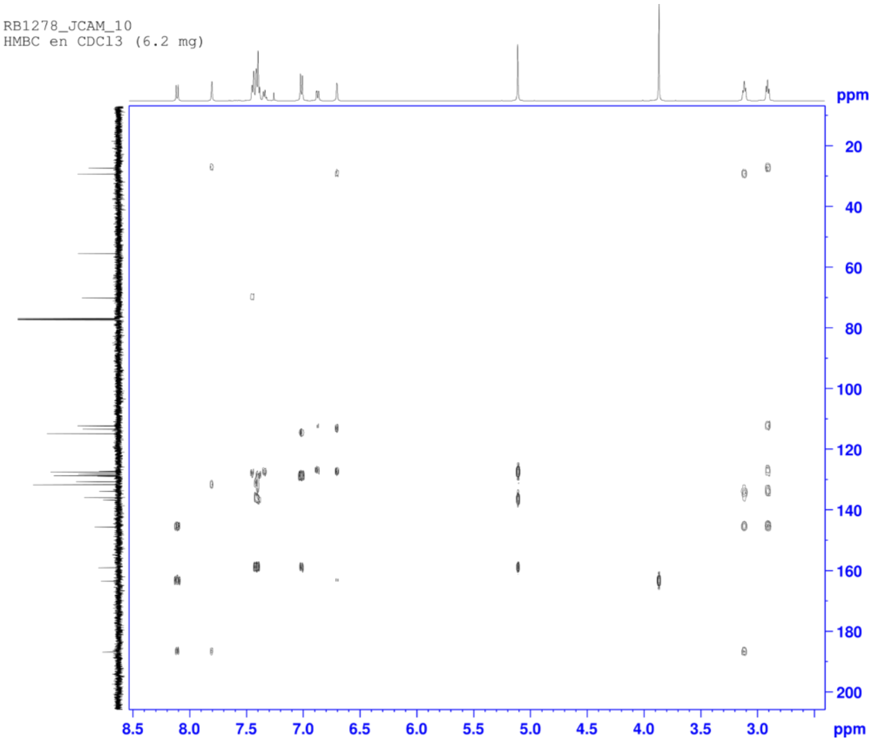

RB1278_JCAM_10
HMBC en CDCl3 (6.2 mg)
ppm
20
40
60
80
100
120
140
160
180
200
8.5
8.0
7.5
7.0
6.5
6.0
5.5
5.0
4.5
4.0
3.5
3.0
ppm

S104. Comp. 39

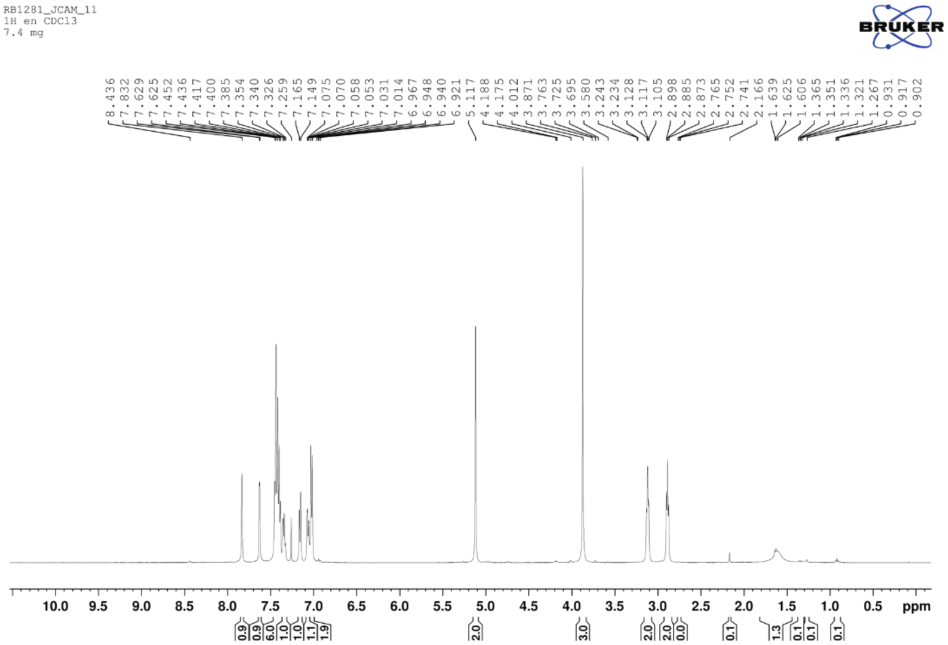

S105. Comp. 39

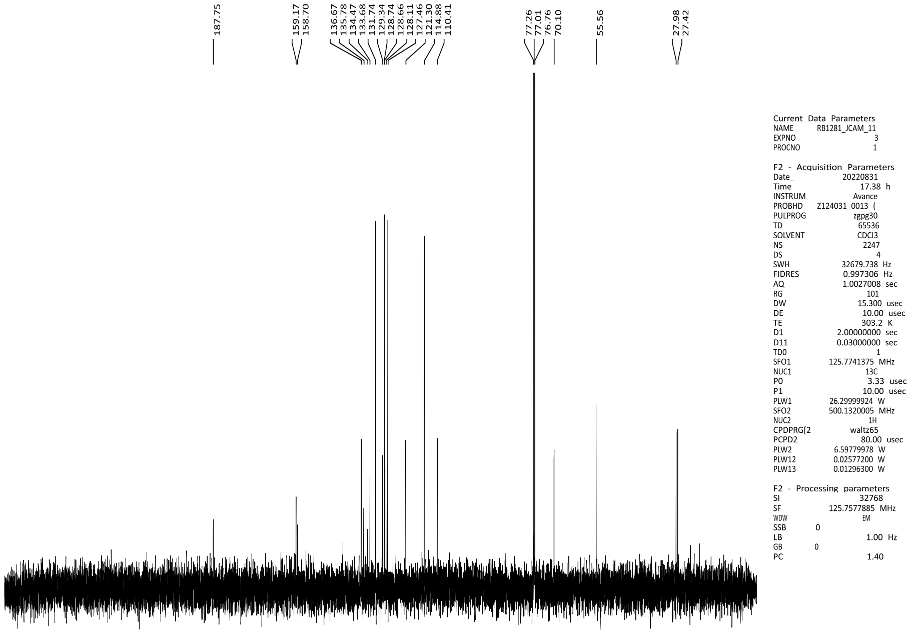

S106. Comp. 39

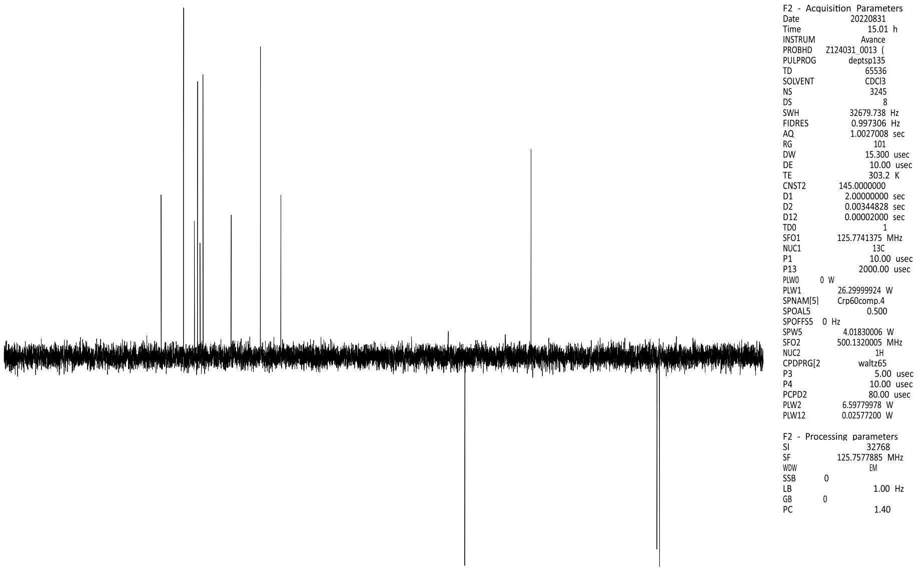

S107. Comp. 39

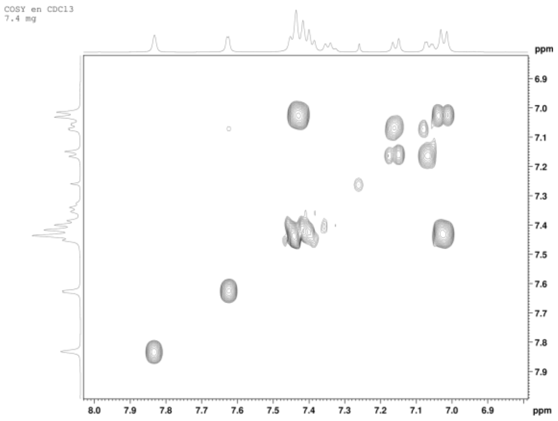

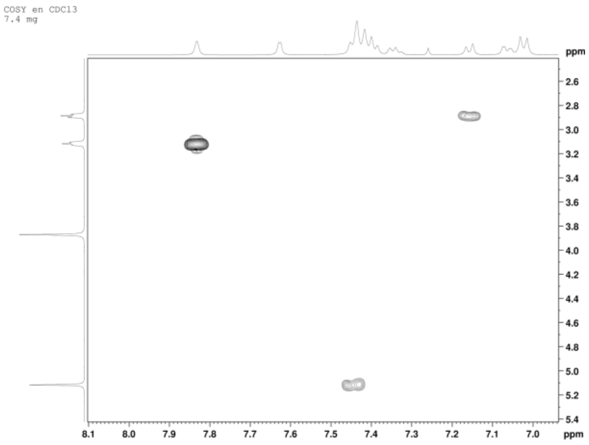

S108. Comp. 39

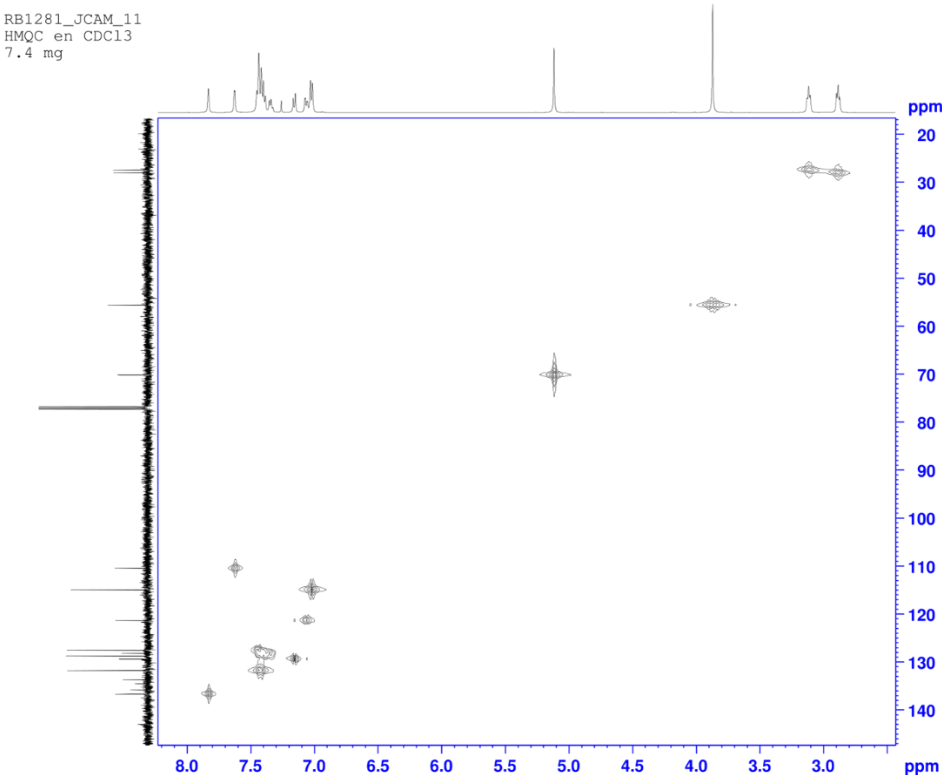

S109. Comp. 39

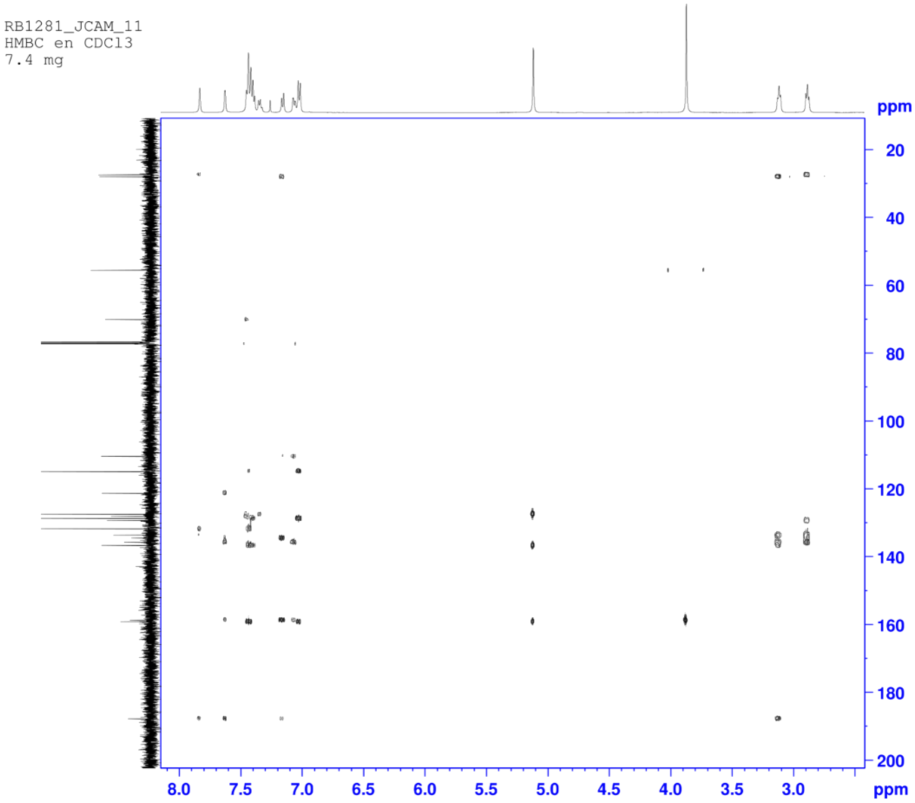

Supplement: Supplementary file 1 [file molecules-28-05569-s001.zip › molecules-2487499-supplementary.pdf]
